# Self-management interventions for adolescents living with HIV: a systematic review

**DOI:** 10.1186/s12879-021-06072-0

**Published:** 2021-05-07

**Authors:** Talitha Crowley, Anke Rohwer

**Affiliations:** 1 Department of Nursing and Midwifery, Faculty of Medicine and Health Sciences, Stellenbosch University, Cape Town, South Africa; 2 Centre for Evidence-based Health Care, Division of Epidemiology and Biostatistics, Department of Global Health, Faculty of Medicine and Health Sciences, Stellenbosch University, Cape Town, South Africa

**Keywords:** Self-management, HIV/AIDS, Adolescents, Systematic review, Protocol

## Abstract

**Background:**

Self-management interventions aim to enable people living with chronic conditions to increase control over their condition in order to achieve optimal health and may be pertinent for young people with chronic illnesses such as HIV. Our aim was to evaluate the effectiveness of self-management interventions for improving health-related outcomes of adolescents living with HIV (ALHIV) and identify the components that are most effective, particularly in low-resource settings with a high HIV burden.

**Methods:**

We considered randomised controlled trials (RCTs), cluster RCTs, non-randomised controlled trials (non-RCTs) and controlled before-after (CBA) studies. We did a comprehensive search up to 1 August 2019. Two authors independently screened titles, abstracts and full texts, extracted data and assessed the risk of bias. We synthesised results in a meta-analysis where studies were sufficiently homogenous. In case of substantial heterogeneity, we synthesised results narratively. We assessed the certainty of evidence using GRADE and presented our findings as summaries in tabulated form.

**Results:**

We included 14 studies, comprising 12 RCTs and two non-RCTs. Most studies were conducted in the United States, one in Thailand and four in Africa. Interventions were diverse, addressing a variety of self-management domains and including a combination of individual, group, face-to-face, cell phone or information communication technology mediated approaches. Delivery agents varied from trained counsellors to healthcare workers and peers. Self-management interventions compared to usual care for ALHIV made little to no difference to most health-related outcomes, but the evidence is very uncertain. Self-management interventions may increase adherence and decrease HIV viral load, but the evidence is very uncertain. We could not identify any particular components of interventions that were more effective for improving certain outcomes.

**Conclusion:**

Existing evidence on the effectiveness of self-management interventions for improving health-related outcomes of ALHIV is very uncertain. Self-management interventions for ALHIV should take into account the individual, social and health system contexts. Intervention components need to be aligned to the desired outcomes.

**Systematic review registration:**

PROSPERO CRD42019126313.

**Supplementary Information:**

The online version contains supplementary material available at 10.1186/s12879-021-06072-0.

## Background

HIV affects 1,740,000 adolescents between the ages of 10 and 19 globally with the highest burden in sub-Saharan Africa [1]. Adolescence is a developmental stage that includes many physical, cognitive and social changes that may be adversely affected by living with a chronic illness [2, 3]. Adolescents living with HIV (ALHIV) may have acquired HIV perinatally, through mother-to-child-transmission or behaviourally through, for example, sexual transmission [4]. Although effective prevention of mother-to-child-transmission strategies have led to fewer children acquiring HIV perinatally, new HIV infections continue to rise amongst adolescents, with 170,000 new infections occurring in 2019 [1]. Globally, adolescent treatment outcomes are poor compared to those of adults, while AIDS is the leading cause of death amongst adolescents in Africa [5].

ALHIV are faced with the dual challenge of having to live with a life-long chronic condition and adhere to treatment, while being confronted with developmental challenges and HIV-related stigma [6]. Supporting them through this vulnerable phase to ensure they make a safe and productive transition to adulthood requires a differentiated care approach – a type of patient-centred approach where HIV care and services are adapted to suit the needs of certain groups [7]. One such approach is self-management support. Self-management has been defined as the “day to day management of chronic conditions by individuals over the course of an illness” [8] (p e26). Self-management support may be particularly important for adolescents, as they can gain skills for lifelong management of their chronic illness. Furthermore, the participative approach to care is likely to appeal to them [9].

Different theories and frameworks to describe the concept of self-management exist. However, key similarities include a focus on the development of self-management abilities and behaviours to manage a chronic condition and achieve health-related outcomes [10–13]. Table 1 illustrates the self-management abilities and self-management behaviours described in the various general chronic disease and HIV-specific self-management theories or frameworks. Self-management interventions usually focus on improving self-management abilities as these are the most amenable to change, empowering people living with a chronic condition to increase control over their condition to achieve optimal health [11].

**Table 1:** Self-management abilities and behaviours as depicted in different frameworks or reviews

Framework	Self-management abilities or processes	Self-management behaviours
Corbin & Strauss (1988) [14] Sattoe et al. (2015) [9]	• Medical management • Behavioural management • Emotional management	Not described
Lorig & Holman (2004) [15]	• Problem solving • Decision making • Utilising resources • Partnering with healthcare providers • Taking action and improving self-efficacy	Not described
Ryan & Sawin (2009) [16] Sawin (2017) [11]	• Enhancing knowledge and beliefs (self-efficacy, outcome expectancy, goal congruence) • Regulating skills and abilities (goal-setting, self-monitoring, reflective thinking, decision making, planning, action, self-evaluation, emotional control) • Social facilitation (influence, support, collaboration)	• Engaging in treatment / treatment adherence • Symptom monitoring
Schilling et al. (2009) [17]	• Collaborating with parents – frequency of parental involvement • Problem solving – adjusting regimen themselves and knowing blood values • Goals – endorsing potential goals	• Performing key care activities • Communicating with parents, healthcare workers, friends
Modi et al. (2012) [10]	• Determining healthcare needs • Seeking disease and treatment related information • Communicating with the medical team	• Taking medication • Attending appointments • Self-monitoring symptoms • Lifestyle modifications • Behavioural compliance with parental instructions • Self-care
Bernardin et al. (2013) [18]	• Self-care skills • Interpersonal skills (communication, relationships, safer sex practices, disclosure) • Technical knowledge (HIV and ART) • Cognitive skills (goal setting, problem solving, decision making, coping skills) • Positive attitudes (self-efficacy, positivity, etc.) • Planning for future roles	• Health and illness management • Use of health services
Grey et al. (2014) [19]	• Illness needs (learning, taking ownership of health needs, performing health promotion activities) • Activating resources (health care, psychological, spiritual, social, community) • Living with a chronic illness (processing emotions, adjusting, integrating illness into daily life, meaning making)	• Acquiring information, monitoring and managing symptoms, taking action to prevent complications, goal setting, decision making, problem solving, planning, evaluating, etc. • Communicating effectively, making decisions collaboratively, seeking support of family and friends, etc. • Dealing with shock and blame, making sense of illness, dealing with stigma, creating a sense of purpose, etc.
Mehraeen et al. (2018) [20]	• Self-management skills not explicitly described	• Medication regimen adherence • Safe sexual behaviour • Physical activity improvement • Symptom management • Attending appointments • Communication with healthcare providers

For the purpose of this review, we chose to focus on interventions that 1) increase ALHIV’s knowledge and beliefs about their disease; 2) improve self-regulation skills and abilities; and 3) assist ALHIV to utilise resources, also referred to as social facilitation. These self-management domains are described in the *Individual and Family Self-Management Theory (IFSMT)* [16] and provide a framework to classify interventions. The *IFSMT* integrates a socio-ecological approach with cognitive theory and takes the individual, social and physical environment into account when explaining self-management [11]. Processing skills, including self-efficacy and knowledge, self-regulation (goal-setting, self-monitoring, emotional-control, etc.), and social facilitation are interrelated processes that are needed to implement self-management behaviours (e.g. taking treatment and attending appointments) [11]. The self-management domains described in the *IFSMT* have been associated with better adherence, health-related quality of life and viral suppression amongst ALHIV [21]. The assumption is that addressing multiple self-management domains will lead to a larger effect on behavioural and health outcomes.

Self-management interventions may differ slightly based on the context and the individual needs of the target group [15, 22]. They may be focused on the adolescent or involve both the adolescent and family as self-management takes place in the context of individual and environmental risk and protective factors [11, 16]. Furthermore, one can classify interventions based on the abilities they are targeting (Table 1).

Effects of self-management interventions on behavioural and health outcomes have been measured in various ways. In their scoping review on self-management interventions for people living with HIV, Bernardin, Toews, Restall and Vuangphan (2013) identified the following key outcomes: well-being and quality of life, health and illness management, and health services use [18]. Sattoe et al. (2015) developed a framework for selecting outcome measures for chronic disease self-management interventions according to whether the interventions target medical, emotional or role management [9]. These outcomes include, but are not limited to, disease knowledge, illness-related self-efficacy, problem-solving, social participation, psychosocial functioning, support by others, coping, and health-related quality of life [9]. A recent systematic review on interventions to improve self-management of adults living with HIV focused on the outcomes as outlined in the *IFSMT*, including physical health, psychosocial outcomes and behavioural outcomes [23].

**Fig. 1:**
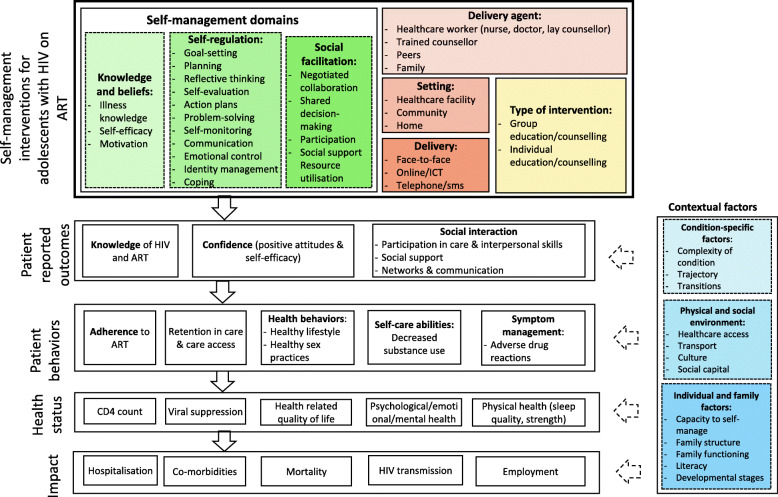
Logic Model

We developed a logic model, informed by existing literature and author expertise using the *IFSMT* [16] as an organising framework (Fig. 1) to depict the components of self-management interventions (according to the self-management domains), the pathway from the intervention to the outcomes, as well as how the intervention interacts with implementation and context variables. It thus helped us to unpack the complexity related to the intervention, the outcomes, and the contextual factors relevant to this review [24].

Although self-management interventions are a promising strategy for improving outcomes in adolescents living with chronic conditions, evidence of effectiveness is lacking. While existing systematic reviews have investigated the effects of self-management interventions on health outcomes, few have specifically focused on ALHIV in settings with scarce resources. Two reviews focused on young people with any chronic condition [9, 25], but not specifically on adolescents. Reviews that focused on HIV-specific self-management interventions [23, 26–29] included mostly adults or excluded studies conducted in Africa [26–30]. Furthermore, there is insufficient evidence of effective components of self-management interventions to inform the development of interventions for ALHIV, particularly in low-resource settings and for interventions focusing on improving social support, managing risk behaviours, and enhancing quality of life [9, 18]. Only one review identified components of self-management interventions that appear to improve specific outcomes across chronic conditions [25]. However, included studies were too heterogeneous to make confident conclusions about the effectiveness of various intervention components. It is, therefore, still not clear which self-management interventions could optimise the health outcomes of ALHIV. Due to their developmental phase, self-management interventions for this group may differ from that of adults [9].

The aim of this systematic review was to determine the effectiveness of self-management interventions to improve health-related outcomes of ALHIV and identify the intervention components that are the most effective, particularly in low-resource settings with a high HIV burden.

### Objectives

The specific objectives were to:

- Assess the effectiveness of self-management interventions on improving health-related outcomes of ALHIV on ART.
- Describe various self-management interventions and their components.
- Determine which interventions may be relevant in low-resource settings with high HIV burden.

## Methods

### Study design

We conducted a systematic review of self-management interventions for ALHIV on ART and reported it according to the PRISMA reporting guidelines [31] (See Additional file 1). Our protocol was registered with the International Prospective Register of Systematic Reviews (PROSPERO) on 23 February 2019 (Reference no. CRD42019126313).

### Eligibility criteria

Studies were eligible for inclusion if they met the following eligibility criteria:

#### Types of studies

We included randomised controlled trials (RCTs), cluster RCTs, non-randomised controlled trials (non-RCTs) and controlled before-after (CBA) studies. We only considered cluster RCTs and CBAs with at least two intervention and two control sites [32].

#### Types of participants

We included adolescents aged 10 to 19, according to the definition of the World Health Organisation (WHO) [2], with a diagnosis of HIV and on ART. We also included studies on young people (10 to 24 years) to account for overlap in the definition of adolescents, young people and youth [33]. Interventions that targeted adolescents and family members as well as studies conducted in low-, middle- and high-income countries were included.

#### Types of interventions

A self-management intervention was defined as any educational strategy to encourage individuals to manage their disease [18]. For the purpose of this review, interventions had to have an educational component that addressed one or more of the following self-management domains as per our logic model (Fig. 1):

1. Knowledge and beliefs: illness knowledge, self-efficacy, motivation.
2. Self-regulation skills and abilities: goal setting, planning, reflective thinking, self-evaluation, action plans, problem-solving, self-monitoring, communication, emotional control, identity management.
3. Social facilitation/utilisation of resources: negotiated collaboration, shared decision-making and participation.

We did not consider interventions that focused on illness knowledge only. Although knowledge is necessary for self-efficacy, knowledge alone does not explain behaviour change [11].

We considered any type of educational intervention, including group education or counselling, and individual education or counselling delivered in any setting (healthcare facility, community, home) by any type of healthcare worker, peers or family members. We included both face-to-face and online information communication technology (ICT) delivery of interventions. Multi-faceted interventions that included components such as short-text-messaging (SMS) reminders or peer support were included if they had an educational component.

*Types of comparisons:* We considered the following comparisons:

1. Self-management interventions addressing one to two self-management domains versus control (no intervention, standard care, other interventions with no self-management component or wait list).
2. Self-management interventions addressing all three self-management domains versus control (no intervention, standard care, other interventions with no self-management component or wait list).
3. Self-management interventions versus other interventions with a different self-management component.

#### Types of outcomes

We included studies reporting on either primary or secondary outcomes. As per our logic model (Fig. 1), we considered the following groups of outcomes: Patient-reported outcomes; behavioural outcomes; measures of health status; and impact outcomes. We included outcomes measured at any point in time following the intervention.

Primary outcomes (as defined by study authors)

1. Patient-reported outcomes: knowledge and understanding of illness (HIV and ART), confidence (positive attitude, self-efficacy, empowerment); motivation; perceived social support; participation in care; interpersonal skills; networks and communication.
2. Patient behaviours: adherence to medication; health/risk behaviours; self-care abilities (decreased substance use); symptom management (e.g. handling adverse effects of drugs).
3. Health status: viral suppression.
4. Health status: CD4 count

Secondary outcomes (as defined by study authors)

1. Health status: health-related quality of life; mental/psychological health; emotional health; physical health.
2. Patient behaviours: clinic attendance/utilisation; retention in care.
3. Impact: Hospitalisation; co-morbidities; all-cause mortality; HIV transmission; employment.

### Information sources and search strategy

An information specialist performed the search on the following electronic databases: MEDLINE PubMed, EMBASE (Ovid), CENTRAL (Cochrane), Africa-Wide (EBSCOhost), CINAHL (EBSCOhost), Web of Science Core Collection: SCI-EXPANDED, CPCI-S, SSCI (Clarivate Analytics), and LILACS (Virtual Health Library). We searched ClinicalTrials.gov (www.ClinicalTrials.gov) and the World Health Organisation (WHO) trials portal (www.who.int/ictrp/en/) to identify unpublished and ongoing studies. In addition, we searched grey literature such as university thesis/dissertation databases and conference abstracts, such as the International AIDS Conference and the Conference on Retroviruses and Opportunistic Infections (CROI). Databases were searched from their inception to 1 August 2019 and there was no restriction on language of publication. To complement the electronic search, we screened reference lists of included studies and relevant systematic reviews. Specialists in the field and authors of the included studies were contacted to identify additional unpublished studies.

We included search terms related to HIV/AIDS, ART, adolescents and self-management, their synonyms, and Medical Subject Headings (MeSH). Additional file 2 contains the full search strategy for all the databases.

### Selection of studies and data extraction

Two review authors used Covidence software to independently screen titles and abstracts to identify potentially eligible studies. We obtained full texts of these studies and independently assessed them to determine eligibility. Disagreements were resolved through discussion. We classified studies as included, excluded with reasons, and ongoing. Authors of studies were contacted in case of missing information.

Two authors independently extracted data using a pre-specified, pre-piloted data extraction form in Covidence. We extracted data on the study design, characteristics of participants, type and description of intervention, outcomes, setting and funding sources. We used a standardised form adapted from the 12-item Template for Intervention Description and Replication (TIDier) checklist [34] to describe components of self-management interventions. This assisted to record important aspects of the intervention such as the theoretical foundation, whether it was tailored for adolescents and the context, the person(s) delivering the intervention and their training, the setting, the specific self-management components addressed, materials used, and procedures followed. We resolved disagreements through discussion.

Two authors independently assessed the risk of bias according to the criteria outlined in the Cochrane Effective Practice and Organisation of Care (EPOC) guidelines [32]. For each study, we assessed the following domains as having high, low or unclear risk of bias: random sequence generation, allocation concealment, baseline outcome measurements, baseline characteristics, incomplete outcome data, blinding, protections against contamination, selective outcome reporting and other risks of bias. We resolved discrepancies through discussion.

### Data analysis and synthesis

One author entered data extracted from individual studies into Review Manager (2014) for analysis and a second author checked the data entry. For dichotomous data, we reported risk ratios or odds ratios with 95% confidence intervals (CIs) to summarise effects. For continuous data, we reported mean differences (MDs) and 95%CIs where studies used the same scale to measure outcomes. To summarise effects, we reported standardised mean differences (SMDs) and 95%CIs where studies used different scales to measure outcomes. We used adjusted measures where studies reported these.

In the case of missing data, we contacted study authors to obtain the data and sent reminders if no response was received. Where authors did not respond or did not provide the data requested, data were reported as missing. We did not impute any data.

We expected high levels of heterogeneity and explored clinical heterogeneity linked to the participants, intervention, setting, outcome measurement and study design, and described these study characteristics in table format. Statistical heterogeneity was assessed using I^2^, Tau^2^ and Chi^2^ statistics. We considered heterogeneity to be significant if Tau^2^ was more than one or if the *p*-value of the Chi^2^ test was less than 0.1. We considered an I^2^ statistic of more than 30% as substantial heterogeneity [35]. Since we did not have more than 10 studies in the meta-analyses, we were not able to explore reporting biases with funnel plots.

Statistical analyses were performed using Review Manager. We used fixed-effect meta-analysis to pool data that was sufficiently homogenous. Where we considered heterogeneity to be high, we did not pool data, but rather presented findings per study in a narrative synthesis. We used forest plots to report data for each outcome, showing either the pooled data for outcomes where meta-analysis was possible or data for each study where we did not pool data.

We had planned to conduct subgroup analysis on type of intervention, delivery agent, age groups and setting. We also planned to carry out sensitivity analyses on primary outcomes to examine the effect of studies with high risk of selection and attrition bias, to examine the effect of imputed data, and to examine the effect of studies that did not stratify results according to required age ranges for adolescents. However, since we only performed meta-analysis for a few outcomes and included few studies, we did not perform subgroup or sensitivity analyses.

### Certainty of the evidence

We assessed the certainty of evidence using GRADE (Grades of Recommendation, Assessment, Development and Evaluation) [36] for the following outcomes: confidence, adherence, risk behaviour, viral load, and mental health (depression). We assessed study limitations, consistency of effect, imprecision, indirectness and publication bias when we considered downgrading the certainty of evidence [37, 38]. For each outcome, we described the certainty of evidence to be very low, low, moderate or high. We used GRADEPro software [39] to generate summaries of the findings in tabulated format.

### Ethical considerations

The systematic review formed part of a larger study with the aim to develop a self-management intervention for ALHIV. This larger study received Health Research Ethics Approval from Stellenbosch University, South Africa (N18/06/064).

## Results

**Fig. 2:**
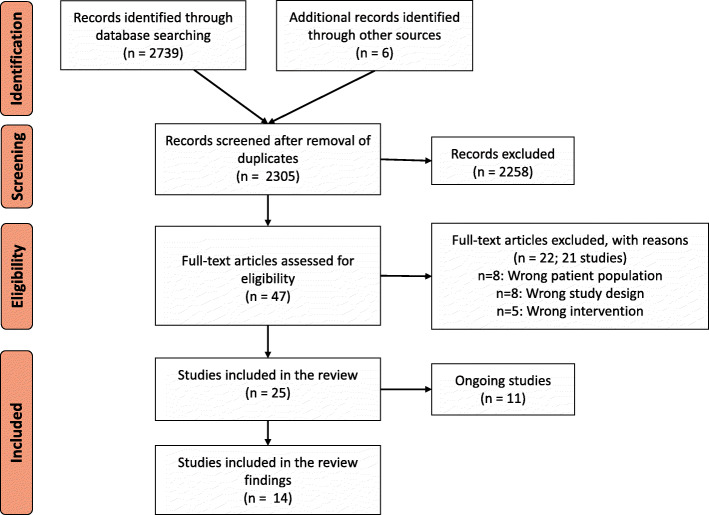
Prisma diagram

We screened titles and abstracts of 2305 studies, and full texts of 47 potentially relevant studies (see Fig. 2). We included 25 studies in this review of which 14 were completed and 11 were ongoing studies (Additional file 3). We excluded 21 studies with reasons provided in Additional file 4.

### Characteristics of included studies

The characteristics of included studies are summarised in Table 2. The majority of studies (*n* = 9) were conducted in the USA, one in Thailand and four in Africa. Settings varied from health facilities to communities in urban and rural areas, and home settings via ICT, phone and gaming platforms. Two studies [47, 50] were non-RCTs, while the rest were RCTs with total sample size varying between *n* = 14 and *n* = 356. Most studies included adolescents and youth of various age groups, with one study [47] focusing on younger children aged 5 to 14. Six of the 14 interventions targeted adolescents or youth with poor adherence or risk behaviours [40, 47, 50, 51, 53, 56]. Studies included both male and female participants, although five studies [48, 49, 54–56] had predominantly male participants (> 75%). One study, the *Vuka Family Programme*, included both adolescents and parents [42], and one study (*Multisystemic Therapy*) included families [50]. Most interventions targeted adolescents on ART, irrespective of the mode of infection (perinatally or behaviourally).

**Table 2:** Summary of characteristics of included studies

ID	Name of intervention	Design	Participant characteristics	Sample size	Participants on ART?	Perinatal or sexual transmission	Country & Setting	Outcomes
Belzer et al. (2014); Sayegh et al. (2018) [40, 41]	Cell Phone Support	RCT^a^ – parallel group	Age 15–24 History of non-adherence (< 90%) 62.2% Male 70.27% Non-Hispanic/ Black/African American	*n* = 37 Intervention = 19 Control (usual care) = 18	Yes	Both - 54% behaviourally infected and 46% perinatally infected	USA High income Urban Community/home	**Confidence** - self-efficacy for adherence **Adherence** **Self-care abilities** - substance use **Viral suppression** **Mental health** - depression **Emotional health** - stress **Psychological health** - problem solving / distraction **Healthcare utilisation**
Bhana et al. 2014) [42]	Vuka Family Programme	RCT – parallel group	Age 10–14 Child and caregiver 51% Female Black South Africans, Zulu Receiving childcare grant: *n* = 45 (82%)	*n* = 65 Intervention = 33 Control (wait list) = 32	Yes	Perinatal	South Africa Middle-income Urban Health facility	**Knowledge** - HIV treatment knowledge **Confidence** - self-identity, self-satisfaction, self-esteem **Social support** - youth and caregiver communication and comfort **Adherence** **Mental health** - strengths and difficulties, child depression
Dow et al. (2018, 2020) [43, 44]	Mental Health Intervention Sauti ya Vijana (SYV; The Voice of Youth)	RCT – parallel group	Age 12–24 50.5% Female	*n* = 93 Intervention = 55 Control (usual care) = 38	Yes	Both (84% perinatal)	Tanzania Low-income Urban Health facility	**Internal stigma**^**b**^ **Adherence** **Viral suppression** **Mental health** - strengths and difficulties, post traumatic stress, depression
Donenberg et al. (2019); Fabri et al. (2015) [45, 46]	Peer-led TI-CBT	RCT – parallel group	Age 14–21	*n* = 356 Intervention = 178 Control (other intervention with no SM components) = 178	Yes	Unclear	Rwanda Low income Urban Health facility	**Adherence** **Health/risk behaviour** - sexual behaviour and drug use **Healthcare utilisation**
Holden et al. (2019) [47]	Stepping Stones	Non-RCT (Historical controls)	Age 5–14 Limited ART adherence/school attendance 53.7% > 10 years 52% Female	*n* = 177 Intervention = 86 Control (usual care) = 91	Yes	Unclear sexual, mostly perinatal	Tanzania Low income Urban Community	**Adherence** **CD4**
Hosek et al. (2018) [48]	ACCEPT (Adolescents Coping, Connecting, Empowering, and Protecting Together)	RCT – parallel group	Age 16–24 Diagnosed with HIV for less than 15 months 68% Gay/lesbian 80.6% Male 51.5% Currently in school 83.5% African American	*n* = 103 Intervention = 57 Control (other intervention with SM components) = 46	Unclear (71.8% taking ART)	Sexual	USA High income Urban Heath facility	**Stigma**^**c**^ **Social support** **Networks and communication** - engagement with healthcare provider **Adherence** **Viral suppression** **CD4** **Health-related quality of life** **Mental Health** - psychological distress **Healthcare utilisation**
Jeffries et al. (2016) [49]	UCare4Life	RCT – parallel group	Age 15–24 Own a phone with text-messaging capability 85% Age 21–24 86% Male 76% Black or African American 68% Diagnosed less than 3 years ago	*n* = 136 Intervention = 91 Control (usual care) = 45	Unclear	Unclear	USA High income Urban Home/ICT	**Adherence** **Self-care abilities** - binge drinking **Viral suppression**
Letourneau et al. (2013) [50]	Multisystemic therapy	Non-RCT – parallel group	Age 9–17 Poor adherence/risky behaviour 65% Female 91% African American	*n* = 34 Intervention = 20 Control (other intervention with SM components) = 14	Yes	Perinatal (33/34)	USA High income Urban Community/ICT	**Adherence** **Viral suppression** **CD4**
Mimiaga et al. (2019) [51]	Positive STEPS	RCT – parallel group	Age 16–24 Self-report adherence difficulty	*n* = 14 Intervention =7 Control (usual care) = 7	Yes	Sexual (82% behaviourally infected)	USA High income Urban Community/ICT/ Health Facility	**Confidence** - adherence readiness, medication taking, self-efficacy **Social support** **Interpersonal skill**s **Adherence**
Naar-King et al. (2006) [52]	Healthy Choices	RCT – parallel group	Age 16–25 51% Male 88% African American 58% Heterosexual	*n* = 62 Intervention = 31 Control (wait list) = 33	Unclear – 1/3 on ART	Sexual (91%)	USA High income Urban Health facility	**Health/risk behaviour** - sexual risk behaviour **Self-care abilities** - illicit drug and alcohol use **Viral suppression**
Naar-King et al. (2009) [53]	Healthy Choices	RCT – parallel group	Age 16–24 At least 1 of 3 HIV risk behaviours 56.6% Heterosexual 52.7% Male 83.3% African American	*n* = 186 Intervention =94 Control (usual care) = 92	Unclear – 34.4% on ART at baseline	Unclear	USA High income Urban Health facility	**Viral suppression**
Rongkavilit et al. (2014) [54]	Healthy Choices	RCT – parallel group	Age 16–25 Mean age 21.7 80% Male 41.8% HIV diagnoses in last 6 months	*n* = 110 Intervention = 55 Control (other intervention with no SM components) = 55	Unclear – 45.5% diagnosed in past 6 months	Yes, 70% MSM	Thailand Middle income Urban Health facility	**Confidence** - self-efficacy for health promotion and risk reduction **Adherence** **Health/risk behaviour** - Consistent condom use **Self-care abilities** - alcohol and substance use **Viral suppression** **Mental health** **Emotional health** - anxiety
Webb et al. (2017) [55]	Mindfulness-based stress reduction (MBSR)	RCT – parallel group	Age 14–22 CD4 count > 200 Mean age 18.7 32.2% Female	*n* = 93 Intervention = 48 Control (Other intervention with no SM components) = 45	Unclear	Unclear	USA High income Urban Health facility	**Mindfulness**^**d**^ **Adherence** **Viral suppression** **CD4** s **Health-related quality of life** **Mental health** - coping **Emotional health** - perceived stress **Psychological health** – problem solving / distraction
Whiteley et al. (2018) [56]	iPhone game (BattleViro)	RCT – parallel group	14–26 Detectable viral load 74% Non-heterosexual Mean age 22.4 78.7% Male 96.7% Black, African American or Haitian	*n* = 61 Intervention = 32 Control (other intervention with no SM components) = 29	Yes	Sexual	USA High income Urban Community/ICT/Home	**Knowledge** - HIV treatment, ART knowledge **Confidence** - motivation, self-efficacy **Social support** **Adherence** **Health / risk behaviour** - Sexual risk behaviour **Viral suppression** **Mental health** - psychological distress

Key: *HCW* Healthcare worker, *ICT* Information Communications Technology

^a^RCT randomised controlled trial

^b^Not an outcome of this review, but included for completeness

^c^Not an outcome of this review, but included for completeness

^d^Mindfulness not an outcome of this review, but included for completeness under Confidence

Primary outcomes were mostly health status outcomes such as viral suppression (*n* = 9) or behaviour outcomes such as adherence (*n* = 12). Seven studies also included mental health as an outcome. No studies assessed impact.

### Summary of interventions

Details of the included interventions are summarised in Tables 3 and 4. Interventions were mostly health facility based (*n* = 9) and delivered either completely face-to-face (*n* = 10) or had a face-to-face component (*n* = 1). Four interventions used platforms such as ICT, telephone, SMS or gaming. Interventions varied from cell phone support, culturally tailored text messages, indigenous leader outreach models, multisystemic therapy, cognitive behavioural therapy, motivational interviewing and mindfulness. Some interventions were brief (4 sessions over 2 months) while one intervention, *Stepping Stones*, comprised up to 29 sessions over a period of 8 months [47]. Three studies used the same intervention, *Healthy Choices,* as a pilot and larger study in the USA that was later adapted for Thailand [52–54]. Half of the interventions used trained counsellors to deliver the intervention. Six interventions addressed all three self-management domains and only one intervention addressed one domain. The domain most often targeted, was *self-regulation*, followed by *knowledge and beliefs*. Table 4 provides an overview of the domains and specific abilities targeted in the completed studies. The abilities the most often targeted were: illness knowledge (8 studies), self-efficacy (8 studies), motivation (7 studies), goal-setting (7 studies), action plans (6 studies), emotional control (6 studies), and negotiated collaboration (6 studies).

**Table 3:** Summary of interventions

ID	Intervention name	Intervention type	Description of intervention	When and how much	Delivery method	Delivery agent
**Completed studies**						
Belzer et al. (2014); Sayegh et al. (2018) [40, 41]	Cell Phone Support	Individual	*Standardised script*: closed and open-ended questions regarding medication review, barriers to taking medication, problem-solving support, referrals and scheduling.	Telephone calls (3-5 min) once or twice a day for 24 weeks	Telephone/SMS^a^	Trained adherence counsellor/HCW^b^
Bhana et al. (2014) [42]	Vuka Family Programme (based on CHAMP)	Group	*Culturally tailored cartoon* storyline used to convey information, accommodate unique needs, family processes (communication, supervision, monitoring & support), mental health, risk behaviour & adherence.	Six sessions over a 3-month period (2 Saturdays a month)	Face-to-face	HCW (lay counsellor supervised by psychiatrist)
Dow et al. (2018, 2020) [43, 44]	Mental Health Intervention Sauti ya Vijana (SYV; The Voice of Youth)	Individual/Group	It incorporates principles of *cognitive behavioural therapy, interpersonal psychotherapy*, and *motivational interviewing*. Includes relaxation, coping with stress, relationships, values, goals etc.	Ten group sessions and 2 individual sessions, 2 jointly with caregivers, each lasting 90 min (3 times a month for a period of 4 months)	Face-to-face	Peers (young adult group leaders)
Donenberg et al. (2019); Fabri et al. (2015) [45, 46]	Peer-led Trauma Informed Cognitive Behavioural Therapy	Group	*Indigenous leader outreach model*: a) psychosocial health education b) relaxation training c) cognitive restructuring d) adherence barriers e) caregiver psychological education.	Six 2-h sessions over 2 months (Sundays); booster session after 12-month assessment	Face-to-face	Peers (indigenous youth leaders)
Holden et al. (2019) [47]	Stepping Stones	Group	A *holistic and transformative approach* includes 3 types of change: psychological (changes in understandings of the self), convictional (revision of belief systems), and behavioural (changes in actions). Gendered and child’s rights focused framework.	A session every morning and every afternoon each weekday. Each community participated in a block of sessions covering Part 1 (sessions 1–15), then, in the next school holidays, a second block for Part 2 (sessions 16–29) (8 months).	Face-to-face	Volunteer facilitators (counsellors)
Hosek et al. (2018) [48]	ACCEPT	Individual/Group	*Disability-stress-coping model* and incorporates information and skills-building activities guided by both social cognitive theory and the information-motivation-behavioural skills model. Focused on youth newly diagnosed with HIV.	Three individual sessions, 6 group sessions of 2 h, occurring weekly (10 weeks)	Face-to-face	HCW & Peer
Jeffries et al. (2016) [49]	UCare4Life	Individual	*Culturally-appropriate text messages* in domains such as treatment and appointment adherence, HIV basics, clinical visits, and risk reduction	Mean of 12 texts per week for 3 months	ICT^c^/SMS	ICT
Letourneau et al. (2013) [50]	Multisystemic therapy (MST)	Individual/Family	Therapists drew upon a menu of evidence-based intervention techniques that included *cognitive-behavioural therapy, parent training, behavioural family systems therapy and communication* skills training.	Families were seen for a mean of 2.2 visits per week across a mean of 6 months	Face-to-face/ICT	Trained counsellor/therapist
Mimiaga et al. (2019) [51]	Positive STEPS (based on ‘Life Steps’)	Individual	*Behavioural technology-based intervention*: Step 1: 2-way personalised text messages; Step 2: adolescent-specific adherence counselling & video vignettes.	Five 1-h sessions delivered over 8 weeks	Face-to-face	Trained counsellor (master’s level)
Naar-King et al. (2006) [52]	Healthy Choices	Individual	*Motivational enhancement* for 2 targeted risk behaviours, combining MI with CBT.	Four sessions (60 min) over 10 weeks	Face-to-face	Trained counsellor
Naar-King et al. (2009) [53]	Healthy Choices	Individual	*Motivational interviewing* for 2 targeted risk behaviours, enhancing intrinsic motivation for change.	Four sessions (60 min) over 10 weeks	Face-to-face	Trained counsellor
Rongkavilit et al. (2014) [54]	Healthy Choices	Individual	*Motivational interviewing* for 3 targeted risk behaviours (sexual risk and either alcohol use or medication adherence). Exploring barriers, change plans.	Four sessions (60 min) over 12 weeks	Face-to-face	Trained counsellor
Webb et al. (2017) [55]	Mindfulness-based stress reduction (MBSR)	Individual	Components: (1) didactic material on topics related to *mindfulness* (2) experiential practice of various mindfulness techniques during group sessions (e.g. meditations, yoga); and (3) discussions on the application of mindfulness to everyday life.	Nine sessions, duration not reported	Face-to-face	Trained counsellor
Whiteley et al. (2018) [56]	iPhone game (BattleViro)	Individual	Multi-level *gaming* intervention for youth living with HIV guided by the Information Motivation and Behavioural Skills (IMB) model. Youth battle HIV and engage with healthcare providers.	Game available for 14 weeks. Twice weekly game-related text messages guided by monitoring device data for first 8 weeks.	ICT/Game	ICT/Game
**Ongoing studies**						
Agwu & Trent (2020) [57]	Tech2Check - technology-enhanced community health nursing intervention	Individual	Field visits by a Community Health Nurse trained in disease intervention protocols, including clinical assessment, case management, counseling, and a behavioural intervention coupled with text messaging support for medication and self-care reminders.	Not stated	Face-to-face/text messaging	HCW
Amico et al. (2019) [58]	TERA (Triggered Escalating Real-Time Adherence)	Individual	Remote ‘face-to-face’ coaching with the assigned adherence coach; 1-way, discrete SMS text message; 2-way interactive outreach SMS from the coach if the electronic dose monitoring (EDM) bottle remains unopened after 1.5 h post dose time; incorporation of dosing data collected via the electronic dose monitoring into follow-up visits to facilitate problem-solving.	Coaching baseline, week 4 and week 12; continuous EDM with SMS outreach (12-week intervention)	Face-to-face/ ICT	Trained counsellors (TERA coaches)
Belzer et al. (2018) [59]	Text message/Cell Phone support (SMART)/Scale-it-Up Programme	Individual	Adherence facilitators that assess if the participant has taken their ART for the day, encourage adherence and engage the participant in brief problem-solving around identified barriers.	Call once a day for 3 months, Mon-Fri	Telephone	Trained counsellors (AFs)
Donenbeg & Dow (2016) [60]	IMPAACT Trauma Informed (TI) Cognitive Behavioural Therapy (CBT) (Group-Based Intervention to Improve Mental Health and Adherence Among Youth Living with HIV in Low-Resource Settings)	Group	Group-based psychosocial health education, cognitive restructuring, and mastery of trauma; identifying and problem-solving barriers to adherence; relaxation training.	Adolescents: Six 2-h TI-CBT group sessions led by IYL during weeks 1–6 and one 2-h booster TI-CBT group session at 6 months; Caregivers: Two 2-h group sessions led by adult study staff during weeks 1–6 and one 2-h booster group session at 6 months; Mixed-gender groups	Face-to-face	Peers (trained indigenous youth peer leaders)
Horvath et al. (2019) [61]	YouThrive	Individual	1) Social support component: interface for participants to interact asynchronously through message posting; 2) ART and HIV related content presented as ‘Thrive tips’; 3) Medication adherence and mood self-monitoring: ‘My check-in’ feature; 4) Goal setting and monitoring: interface called ‘My Journey’; 5) weekly SMS to encourage youth to visit website; 6) Game mechanics: YT uses points that accumulate.	Access to website for 5 months, 3 thrive tips per day, weekly SMS engagement message	ICT	ICT – moderated by trained research staff
Mimiaga et al. (2018) [62]	Positive STEPS	Individual	Step 1) Low-intensity, daily, personalised, two-way text messages; Step 2) Each session incorporates adolescent-specific adherence counseling, digital video vignettes focused on adherence problems and challenges.	Step 1: 12 months; Step 2: five sessions of 50 min (duration of intervention unclear)	ICT/Face-to-face	Trained counsellor (master’s level)
Outlaw & Naar (2020) [63]	Motivational Enhancement System for Adherence (MESA)	Individual	Two computer-based sessions: 1) decisional balance exercise, confidence modules and goal setting, activities to boost self-efficacy. Personal feedback immune status and HIV knowledge. 2) Adherence behaviour over previous month, with actual adherence feedback, adherence behaviour over previous month and consequences of that behaviour.	2 brief sessions one month apart	ICT	Computer-delivered
Arnold et al. (2019) [64]	Stepped Care Intervention	Individual	Level 1) Enhanced Care plus automated messaging and monitoring intervention (AMMI). Level 2) Secure, private online/social media peer-support intervention. Level 3) Participants who fail to achieve viral suppression at levels 1 or 2 of the intervention will be assigned to a coaching intervention.	Level 1 text messages: 1–5 text messages per day for 24 months; Level 2 not reported; Level 3 not reported	ICT/face-to-face/phone	Trained counsellors (coaches)
Sam-Agudu et al. (2017) [65]	Adolescent Coordinated Transition	Group	Altering paediatric-adult visits; monthly peer-led organised support group with curriculum content; a case management team consisting of a physician, a nurse, and a trained patient advocate.	4 times during pre-transfer (at 3, 6, 9, and 12 months); 3 times after transfer to adult clinic (at 15, 18 and 21 months) (total 36 months)	Face-to-face	HCW & Peer
Sibinga (2018) [66]	Mindfulness-based stress reduction (MBSR)	Group	1) Material related to mindfulness, meditation, yoga, mind-body connection; 2) Experiential practice of mindful meditation; 3) Group discussions focused on problem-solving related to barriers to effective practice.	2-h sessions every week for 8 weeks and one 3-h session in week 9	Face-to-face	Trained counsellor (MBSR instructor)
Subramanian et al. (2019) [67]	Integrated Care Delivery of HIV Prevention and Treatment (SHIELD)	Group	SHIELD: Educational modules on HIV prevention and treatment, general wellness, SRH, communication skills etc.; youth clubs.	Modules: a three-session, six-module program; Youth clubs: meet twice per month for 12 months; Modules for family members: 2 sessions, 4-module programme	Face-to-face	Peers for youth clubs; Unclear who will facilitate educational sessions

^a^Short text messaging

^b^Healthcare worker

^c^Information Communication Technology

**Table 4:** Self-management components and abilities targeted by interventions

Study ID	Intervention name	Intervention aim	Self-management domains addressed	Self-management abilities targeted
Belzer et al. (2014); Sayegh et al. (2018) [40, 41]	Cell Phone Support	To provide participating youth living with HIV with a consistent, accessible and supportive relationship in which problem-solving solutions to adherence barriers along with tangible assistance and informational advice.	***Self-regulation***	Problem-solving
			***Social facilitation***	Negotiated collaboration
Bhana et al. 2014) [42]	Vuka Family Programme (based on CHAMP)	To deliver critical information to facilitate discussions and problem-solving within and between families in multi-family groups.	***Knowledge and beliefs***	Illness knowledge
			***Self-regulation***	Problem solving Communication Identity management
Dow et al. (2018, 2020) [43, 44]	Mental Health Intervention Sauti ya Vijana (SYV; The Voice of Youth)	To improve treatment adherence, reduce mental health symptoms and increase youth resilience.	***Knowledge and beliefs***	Illness-knowledge Self-efficacy Motivation
			***Self-regulation***	Coping Goal setting Emotional control Self-evaluation Identity management Social support
			***Social facilitation***	Negotiated collaboration
Donenberg et al. (2019); Fabri et al. (2015) [45, 46]	Peer-led Trauma Informed Cognitive Behavioural Therapy	To increase ART adherence by reducing depression, trauma, and gender-based violence (GBV).	***Knowledge and beliefs***	Illness knowledge
			***Self-regulation***	Problem solving Coping Emotional control Identity management
Holden et al. (2019) [47]	Stepping Stones	To build resilience among children with HIV.	***Knowledge and beliefs***	Illness knowledge Self-efficacy Motivation
			***Self-regulation***	Goal setting Action plans Assertiveness Emotional control Self-evaluation
			***Social facilitation***	Negotiated collaboration Social support
Hosek et al. (2018) [48]	ACCEPT	To assist young adults newly diagnosed with HIV to engage in the healthcare system in order to improve medical, psychological and public health outcomes.	***Knowledge and beliefs***	Illness knowledge
			***Self-regulation***	Decision-making Action plans Coping Goal setting Emotional control
			***Social facilitation***	Social support Shared-decision-making
Jeffries et al. (2016) [49]	UCare4Life	To increase retention in care and HIV medication adherence*.*	***Knowledge and beliefs***	Illness knowledge Self-efficacy
			***Self-regulation***	Self-monitoring - reminders
			***Social facilitation***	Participation
Letourneau et al. (2013) [50]	Multisystemic therapy (MST)	To address medication adherence problems in children with HIV.	***Self-regulation***	Communication
			***Social facilitation***	Negotiated collaboration
Mimiaga et al. (2019) [51]	Positive STEPS (based on ‘Life Steps’)	To address adolescent-specific barriers to HIV medication adherence among heterosexual and Lesbian-Gay-Bisexual (LGB), perinatally and behaviourally infected youth.	***Knowledge and beliefs***	Illness knowledge Self-efficacy Motivation
			***Self-regulation***	Goal setting Action plans Problem solving Emotional control Coping
			***Social facilitation***	Social support Negotiated collaboration Participation
Naar-King et al. (2006) [52]	Healthy Choices	To move people along the stages of change (motivation for change), help them to review costs and benefits (decisional balance), and improve self–efficacy.	***Knowledge and beliefs***	Self-efficacy Motivation
			***Self-regulation***	Goal setting Planning Action plans Self-monitoring Reflective thinking
			***Social facilitation***	Resource utilisation
Naar-King et al. (2009) [53]	Healthy Choices	To move people along the stages of change, help them to review costs and benefits (decisional balance), and improve self-efficacy; to improve viral load (viral suppression).	***Knowledge and beliefs***	Self-efficacy Motivation
			***Self-regulation***	Goal setting Planning Action plans Self-monitoring Reflective thinking
Rongkavilit et al. (2014) [54]	Healthy Choices	To increase motivation for healthy behaviours – specifically risk behaviours.	***Knowledge and beliefs***	Self-efficacy Motivation
			***Self-regulation***	Goal setting Planning Action plans Self-monitoring Reflective thinking
Webb et al. (2017) [55]	Mindfulness-based stress reduction (MBSR)	To increase mindfulness and other elements of self-regulation as well as improved HIV disease management; to enhance present-focused awareness, reducing preoccupation with the past and the future.	***Self-regulation***	Problem-solving Emotional control Coping
Whiteley et al. (2018) [56]	iPhone game (BattleViro)	To empower youth to improve adherence by increasing information, motivation and behavioural skills.	***Knowledge and beliefs***	Illness knowledge Self-efficacy Motivation
			***Social facilitation***	Negotiated collaboration Social support

The theories mostly used to develop the interventions included social influence theories such as Social Cognitive Theory, Cognitive Behaviour Theory (CBT), Ecological Systems Theory and Information, and Motivation and Behaviour Skills (IMBS).

In Africa, the four completed studies as well as the ongoing studies used predominantly group education and counselling delivered by lay workers or peers with no ICT/phone interventions.

### Risk of bias of included studies

**Fig. 3:**
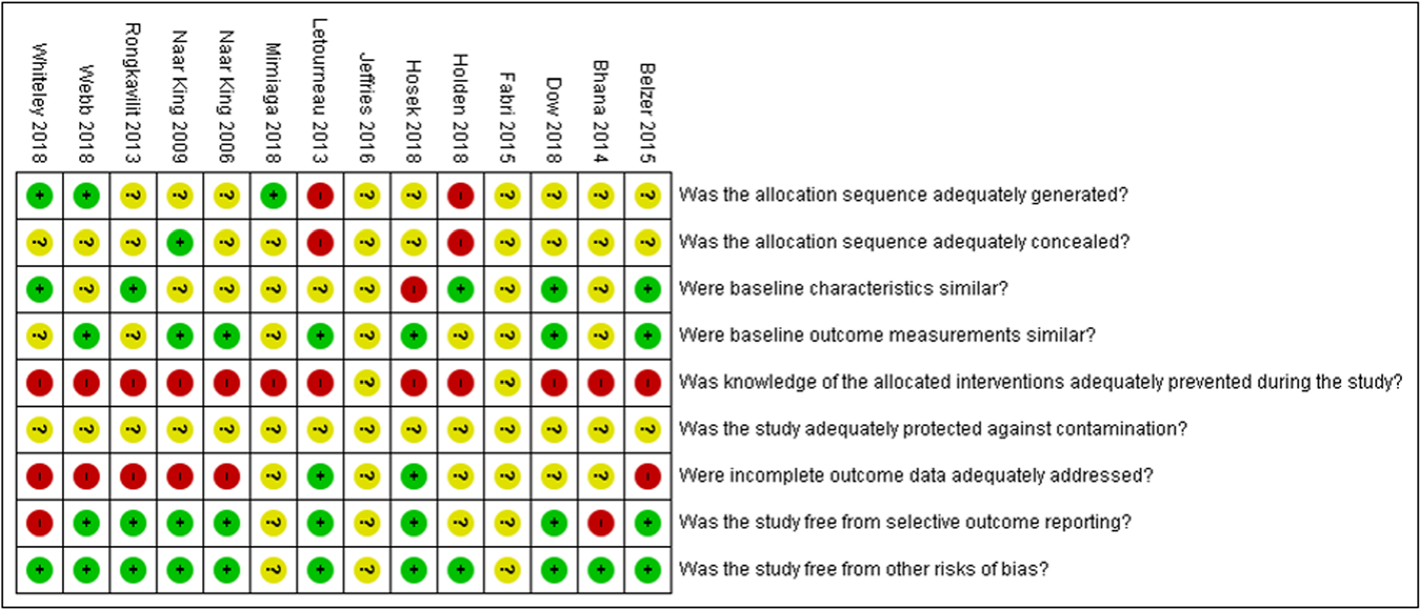
Summary of risk of bias

Overall, risk of bias across domains was moderate to high across studies and is summarised in Fig. 3. Additional file 5 contains the detailed risk of bias judgements per study. We were not able to access the full study report for two studies [46, 49] and assessed all domains as having an unclear risk of bias due to missing information. We judged two non-RCTs [47, 50] to have a high risk of selection bias. The remaining studies did not report adequately on sequence generation and allocation concealment and were judged to be of unclear risk of bias. All studies had a high risk of performance bias, as the nature of the interventions did not allow blinding of participants and personnel and most outcomes were measured subjectively. We judged the risk of attrition bias to be low for two studies [47, 50] and high for six studies [40, 41, 52–56] due to high rates of loss-to-follow-up. The risk of attrition bias was unclear for the remaining studies.

### Effects of self-management interventions on outcomes

#### Comparison 1: self-management interventions addressing one to two self-management domains vs control

We included seven studies in this comparison [40, 42, 45, 46, 53–56]. One study, *Peer-led Trauma Informed Cognitive Behavioral Therapy* [45, 46], did not publish any outcome data in available articles and authors could not provide any data when contacted. Forest plots containing data for all outcomes are available in Additional file 6. The summary of findings and GRADE certainty of evidence ratings are presented in Table 5.

**Table 5:** Summary of Findings comparison 1

Summary of findings: Self-management interventions compared to control in adolescents living with HIV					
Patient or population: Adolescents living with HIV; Setting: Low-, middle-, and high-income countries; Intervention: Self-management interventions with 1–2 components; Comparison: Usual care					
Outcome	Follow-up	Pooled effect (95%CI)	No. of participants (studies)	Certainty of evidence (GRADE)	Comments
**Confidence**	3 months	MD 0.35 (0.01 to 0.69)	33 (1 trial)	⨁◯◯◯ VERY LOW ^a,b,c^	HIV self-management interventions compared to usual care for adolescents living with HIV may increase confidence at 3-month follow-up and may make little or no difference to confidence at 4-, 6-, 9- and 12-month follow-ups, but the evidence is very uncertain.
	4 months	MD 0.00 (−0.26 to 0.26)	96 (1 trial)		
		MD 0.35 (−2.12 to 2.82)	61 (1 trial)		
	6 months	MD 0.14 (−0.32 to 0.60)	31 (1 trial)		
	9 months	MD 0.10 (−0.17 to 0.37)	91 (1 trial)		
	12 months	MD 0.21 (−0.22 to 0.64)	31 (1 trial)		
**Adherence (self-reported)**	3 months	SMD 0.19 (−0.09 to 0.48)	198 (3 trials)	⨁◯◯◯ VERY LOW ^a,b,c^	HIV self-management interventions compared to usual care for adolescents living with HIV may make little or no difference to self-reported adherence at 3-, 6- and 9-month follow-ups, and may increase adherence at 12-month follow-up, but the evidence is very uncertain.
	6 months	SMD 0.71 (−0.02 to 1.44)	31 (1 trial)		
	9 months	SMD 0.11 (−0.30 to 0.52)	91 (1 RCT)		
	12 months	SMD 1.16 (0.39 to 1.93)	31 (1 trial)		
**Adherence (Electronic pill monitoring)**	4 months	SMD 0.29 (− 0.21 to 0.8)	61 (1 trial)	⨁◯◯◯ VERY LOW ^a,b,c^	HIV self-management interventions compared to usual care for adolescents living with HIV may make little or no difference to adherence at 4-month follow-up, but the evidence is very uncertain.
**Sexual risk behaviour**	4 months	MD 0.4 (−0.76 to 1.56)	96 (1 trial)	⨁◯◯◯ VERY LOW ^a,b,c^	HIV self-management interventions compared to usual care for adolescents living with HIV may make little or no difference to sexual risk behaviour at 4- and 9-month follow-ups, but the evidence is very uncertain.
	9 months	MD −0.90 (−2.39 to 0.59)	91 (1 trial)		
**Viral load (log 10)**	4 months	MD −0.12 (− 0.45 to 0.2)	157 (2 trials)	⨁⨁◯◯ LOW ^a,b^	HIV self-management interventions compared to usual care for adolescents living with HIV may make little or no difference to viral load at 4- and 9-month follow-ups. At 6- and 12-month follow-ups, HIV self-management interventions compared to usual care may decrease viral load, but the evidence is very uncertain.
	6 months	MD −1.70 (−2.65 to − 0.75)	30 (1 trial)	⨁◯◯◯ VERY LOW ^a,b,c^	
	9 months	MD −0.02 (− 0.30 to 0.26)	237 (2 trials)	⨁⨁◯◯ LOW ^a,b^	
	12 months	MD −1.00 (− 1.89 to −0.11)	31 (1 trial)	⨁◯◯◯ VERY LOW ^a,b,c^	
**Depression**	3 months	SMD −0.27 (− 0.56 to 0.01)	194 (3 trials)	⨁◯◯◯ VERY LOW ^a,b,c^	HIV self-management interventions compared to usual care for adolescents living with HIV may make little or no difference to depression at 3-, 6-, 9- and 12-month follow-ups, but the evidence is very uncertain.
	6 months	SMD −0.57 (−1.29 to 0.15)	31 (1 trial)		
	9 months	SMD −0.12 (− 0.48 to 0.25)	117 (2 trials)		
	12 months	SMD −0.26 (− 0.97 to 0.45)	31 (1 trial)		

*CI* Confidence interval, *MD* Mean difference, *SMD* Standardised mean difference

GRADE Working Group: Grades of evidence

High certainty: We are very confident that the true effect lies close to that of the estimate of the effect

Moderate certainty: We are moderately confident in the effect estimate: The true effect is likely to be close to the estimate of the effect, but there is a possibility that it is substantially different

Low certainty: Our confidence in the effect estimate is limited: The true effect may be substantially different from the estimate of the effect

Very low certainty: We have very little confidence in the effect estimate: The true effect is likely to be substantially different from the estimate of effect

Footnotes: Explanation of GRADE certainty of evidence

^a^ Downgraded by 1 for serious concerns about risk of bias in at least one domain

^b^ Downgraded by 1 for indirectness, as studies did not only include adolescents (age 10 to 19)

^c^ Downgraded by 1 for serious concerns about imprecision with wide 95%CI intervals and small sample sizes

### Patient reported outcomes

#### Knowledge and understanding of illness

Two studies found little to no difference between groups at three [42] and four [56] months follow-up.

#### Confidence (self-efficacy for taking ART)

One study, *Cell Phone Support* [40, 41], found a small increase in self-efficacy for health promotion and risk reduction (MD 0.35 95% CI (0.01 to 0.69), *n* = 33, very low certainty evidence) in the group receiving the self-management intervention compared to the control group at the three-month follow-up. At the four-month follow-up, two studies [54, 56] found little to no difference between groups (very low certainty evidence). At the six [40, 41], nine [54] and 12-month [40, 41] follow-ups, studies found little to no difference between groups (very low certainty evidence). One study [42] did not report data for this outcome.

#### Motivation for taking ART

Studies found little to no difference between groups at three [40, 41], four [54], six [40, 41], nine [40, 41, 54], and 12-month [40, 41] follow-ups.

#### Mindfulness

One study, *Mindfulness-Based Stress Reduction* [55], found a slight increase in mindfulness scores in the group receiving the self-management intervention compared to the control group (MD 0.65, 95%CI 0.06 to 1.24, *n* = 71) at the three-month follow-up.

#### Social support

One study, the *Vuka Family Programme* [42], found a slight increase in youth and caregiver communication and comfort scores (MD 0.8, 95%CI 0.31 to 1.28, *n* = 65) among participants receiving the self-management intervention compared to the control group at the three-month follow-up. At the four-month follow-up, one study [56] found little to no difference between groups offering social support for adherence.

None of the included studies reported on participation in care, interpersonal skills or networks and communication.

### Patient behaviours

#### Adherence to ART

The pooled effect of three studies included in the meta-analysis [42, 55, 56] showed little to no difference in self-reported adherence between groups (SMD 0.19, 95%CI − 0.09 to 0.48; *n* = 198, 3 studies, very low certainty evidence) at the three to four-month follow-up. One study [56] also used electronic pill monitoring to measure adherence at the three-month follow-up and found little to no difference between groups (SMD 0.29, 95%CI − 0.231 to 0.80, *n* = 61, very low certainty evidence). Two studies found little to no difference between groups at six [40, 41] and nine-month [54] follow-ups (very low certainty evidence). One study, *Cell Phone Support* [40, 41], found a large increase in adherence scores in the group receiving the self-management intervention at the 12-month follow-up (SMD 1.16, 95%CI 0.39 to 1.93, *n* = 33, very low certainty evidence).

#### Sexual risk behaviour

One study [54] found little to no difference between groups at the four and nine-month follow-up (very low certainty evidence).

#### Self-care abilities (substance use)

Studies found little to no difference between groups at the three [40, 41], four [54], six [40, 41] and nine-month [40, 41, 54] follow-ups. One study, *Cell Phone Support* [40, 41], found a decrease in substance use among participants receiving the self-management intervention at the 12-month follow-up (MD -5.38, 95%CI − 10.16 to − 0.60, *n* = 32) compared to the control group.

#### Healthcare utilisation

One study [40, 41] found little to no difference between groups that made healthcare visits over 12 weeks prior to assessments done at three, six, nine and 12 months.

None of the included studies reported on symptom management or retention in care.

### Health status

#### Viral suppression

One study [55] reported on the number of participants with a viral load (log10) of less than 2 at the three-month follow-up and found little to no difference between groups (very low certainty evidence). The pooled effect of two studies [54, 56] showed little to no difference in viral load (log10) between groups (MD -0.12, 95%CI − 0.42 to 0.20, *n* = 157, low certainty evidence) at the four-month follow-up. One study, *Cell Phone Support* [40, 41], found a decrease in the viral load (log10) among participants receiving the self-management intervention, compared to the control group, at the six-month follow-up (MD -1.70, 95%CI − 2.65 to − 0.75, *n* = 30, very low certainty evidence). The pooled effect of two studies [53, 54] found little to no difference in viral load (log10) between groups at the nine-month follow-up (MD -0.02, 95%CI − 0.30 to 0.26, *n* = 237, low certainty evidence). One study, *Cell Phone Support* [40, 41], found a decrease in viral load (log10) among participants receiving the self-management intervention compared to the control group at the 12-month follow-up (MD -1.00, 95%CI − 1.89 to − 0.11, *n* = 31, very low certainty evidence).

#### CD4 count

One study [40, 41] found little to no difference between groups at the three-month follow-up.

#### Quality of life

One study, *Mindfulness-Based Stress Reduction* [55], found a slight increase in life satisfaction scores among participants receiving the self-management intervention compared to the control group (MD 0.57, 95%CI 0.01 to 1.13, *n* = 72) at the three-month follow-up, but found little to no difference for illness burden and illness anxiety.

#### Emotional health

The pooled effect for two studies [37, 48, 53] showed little to no difference between groups for perceived stress at the three-month follow-up (MD -0.27, 95%CI − 0.66 to 0.11, *n* = 105). One study, *Cell Phone Support* [40, 41], found little to no difference between groups at six and nine months, and found a slight decrease in perceived stress among participants who received the self-management intervention compared to the control group at the 12-month follow-up (MD -1.90, 95%CI − 3.53 to − 0.27, *n* = 31). One study [54] reported on anxiety and found little to no difference between groups at the four and nine-month follow-ups.

#### Mental health

The pooled effect of three studies [40–42, 54] showed little to no difference in depression scores between groups (SMD -0.27, 95%CI − 0.56 to 0.01, *n* = 194, very low certainty evidence) at the three-month follow-up. There was little to no difference between groups’ depression scores at the six [40, 41], nine [40, 41, 54] and 12-month [40, 41] follow-up (very low certainty evidence).

#### Psychological health

The pooled effect of two studies [40, 41, 55] showed little to no difference between groups for problem-solving (SMD 0.33, 95%CI − 0.05 to 0.72, *n* = 105) at the three-month follow-up. One study [40, 41] found little to no difference between groups for problem-solving at the six, nine and 12-month follow-up. The pooled effect of two studies [40, 41, 55] showed little to no difference between groups for distraction at the three-month follow-up (SMD 0.17, 95%CI − 0.22 to 0.55, *n* = 105). One study [40, 41] found little to no difference between groups for distraction at the six, nine and 12-month follow-ups.

None of the included studies reported on physical health.

### Impact

None of the included studies reported on hospitalisation, co-morbidities, all-cause mortality, HIV transmission or employment.

#### Comparison 2: self-management interventions addressing all three components vs control groups

We included five studies in this comparison [43, 44, 47, 49, 51, 52]. Forest plots containing data for all outcomes are available in Additional file 6. The summary of findings and GRADE certainty of evidence ratings are presented in Table 6.

**Table 6:** Summary of findings comparison 2

Summary of findings: Self-management interventions compared to control in adolescents living with HIV					
Patient or population: Adolescents living with HIV; Setting: Low-, middle-, and high-income countries; Intervention: Self-management interventions with all 3 components; Comparison: Usual care					
Outcome	Follow-up	Pooled effect (95%CI)	No. of participants (studies)	Certainty of evidence (GRADE)	Comments
**Confidence**	6 months	MD 0.80 (−0.12 to 1.72)	93 (1 trial)	⨁◯◯◯ VERY LOW ^a,b,c^	HIV self-management interventions compared to usual care for adolescents living with HIV may make little or no difference to confidence at 6-month follow-up, but the evidence is very uncertain.
**Adherence (self-reported)**	6 months	SMD 0.67 (0.27 to 1.07)	107 (2 trials)	⨁◯◯◯ VERY LOW ^a,b,c^	HIV self-management interventions compared to usual care for adolescents living with HIV may increase self-reported adherence at 6-month follow-up, but the evidence is very uncertain.
**Adherence (more than 95%)**	9 months	RR 1.14 (1.20 to 1.65)	177 (1 trial)	⨁◯◯◯ VERY LOW ^a,b,c^	HIV self-management interventions compared to usual care for adolescents living with HIV may increase the likelihood of achieving over 95% adherence at 9-month follow-up, but the evidence is very uncertain.
**Sexual risk behaviour**	3 months	MD −11.97 (−25.45 to 1.51)	51 (1 trial)	⨁◯◯◯ VERY LOW ^a,b,c^	HIV self-management interventions compared to usual care for adolescents living with HIV may make little or no difference to sexual risk behaviour at 3-month follow-up, but the evidence is very uncertain.
**Viral load (log 10)**	3 months	MD −0.66 (−1.21 to − 0.11)	51 (1 trial)	⨁◯◯◯ VERY LOW ^a,b,c^	HIV self-management interventions compared to usual care for adolescents living with HIV may decrease viral load at 3-month follow-up and may make little to no difference at 6-month follow-up, but the evidence is very uncertain.
	6 months	MD −0.84 (−1.69 to 0.01)	93 (1 trial)		
**Depression**	6 months	MD −0.60 (−2.67 to 1.47)	93 (1 trial)	⨁◯◯◯ VERY LOW ^a,b,c^	HIV self-management interventions compared to usual care for adolescents living with HIV may make little or no difference to depression at 6-month follow-up, but the evidence is very uncertain.

*CI* Confidence interval, *MD* Mean difference, *SMD* Standardised mean difference, *RR* Risk ratio

GRADE Working Group: Grades of evidence

High certainty: We are very confident that the true effect lies close to that of the estimate of the effect.

Moderate certainty: We are moderately confident in the effect estimate: The true effect is likely to be close to the estimate of the effect, but there is a possibility that it is substantially different.

Low certainty: Our confidence in the effect estimate is limited: The true effect may be substantially different from the estimate of the effect

Very low certainty: We have very little confidence in the effect estimate: The true effect is likely to be substantially different from the estimate of effect.

Footnotes: Explanation of GRADE certainty of evidence

^a^ Downgraded by 1 for serious concerns about risk of bias in at least one domain

^b^ Downgraded by 1 for indirectness, as studies did not only include adolescents (age 10 to 19)

^c^ Downgraded by 1 for serious concerns about imprecision with wide 95%CI intervals and small sample sizes

### Patient reported outcomes

#### Confidence

One study, *Sauti ya Vijana* [43, 44], reported on the internal stigma score (negative self-image) and found little to no difference in scores at the six-month follow-up (very low certainty evidence). One study [51] did not report data for this outcome.

One study, *Positive STEPS* [51], measured social support and interpersonal skills but did not report any data for these outcomes. None of the included studies reported on knowledge and understanding of illness, motivation for taking ART, mindfulness, participation in care or networks and communication.

### Patient behaviours

#### Adherence to ART

Two studies, *Sauti ya Vijana* and *Positive STEPS* [43, 44, 51], were included in the meta-analysis and showed an increase in adherence among participants receiving the self-management intervention compared to the control group that formed the baseline at the four or six-month follow-up (SMD 0.67, 95%CI 0.27 to 1.07, *n* = 107, very low certainty evidence). One study [43, 44] also reported ART hair concentration as a measure of adherence and found little to no difference between groups and there was no change from the baseline to the six-month follow-up (very low certainty evidence). One study, *Stepping Stones* [47], reported on the number of participants that had achieved over 95% adherence based on pill counting and self-reporting at the nine-month follow-up. They found that participants receiving the self-management intervention were 41% more likely to have achieved over 95% adherence compared to the control group (risk ratio (RR) 1.41, 95%CI 1.20 to 1.65, *n* = 177, very low certainty evidence). One study measured adherence but did not report data [49].

#### Sexual risk behaviour

One study [52] found little to no difference between groups at three months follow-up.

#### Self-care abilities (substance use)

Naar-King et al. (2006) [52] found little to no difference between groups for alcohol use, as well as for marijuana use. One study, *UCare4Life* [49], did not report any data for this outcome.

None of the included studies reported on symptom management, retention in care or healthcare utilisation.

### Health status

#### Viral suppression

One study, *Healthy Choices* [52], found a decrease in viral load (log10) among participants receiving the self-management intervention compared to the control group at the three-month follow-up (MD -0.66, 95%CI − 1.21 to − 0.11, very low certainty evidence). Dow (2018, 2020) [43, 44] found little to no difference in viral load (log10) between groups at the six-month follow-up (very low certainty evidence). One study [49] did not report any data for this outcome.

#### CD4 count

One study, *Stepping Stones* [47], found an increase in CD4 count among participants receiving the self-management intervention compared to the control group at the nine-month follow-up (MD 156.82, 95%CI 43.48 to 270.16, *n* = 177).

#### Psychological/mental health

One study, *Sauti ya Vijana* [43, 44], found little to no difference between groups for depression and other mental health measures.

None of the included studies reported on quality of life, emotional health or physical health.

### Impact

None of the included studies reported on hospitalisation, co-morbidities, all-cause mortality, HIV transmission or employment.

#### Comparison 3: self-management interventions vs other interventions with self-management components

We included two studies in this comparison [48, 50]. Hosek et al. (2018) (*Project ACCEPT for Newly HIV Diagnosed Youth*) analysed longitudinal data collected at three, six and 12 months post-intervention, and reported longitudinal outcomes associated with the intervention group over time [48]. Letourneau et al. (2013) (*Multisystemic Therapy for Poorly Adherent Youth*) collected data at three, six and 12 months post-intervention and reported the change in outcome slopes between groups over time [50]. Neither of the studies reported means and standard deviations at particular follow-up periods. Both studies had controls that included self-management components. For example, the control for *Project ACCEPT* was health education that included all three self-management components and for *Multisystemic Therapy,* the control (usual care with motivational interviewing) included one self-management component.

### Patient reported outcomes

#### Confidence

*Project ACCEPT* [48] found little to no difference in perceived HIV stigma scores between groups over time.

#### Social support

One study, *Project ACCEPT* [48], found little to no difference between groups over time.

#### Networks and communication

One study, *Project ACCEPT* [48], found little to no difference in engagement with healthcare providers between groups over time.

None of the included studies reported on knowledge and understanding of illness, motivation for taking ART, mindfulness, participation in care or interpersonal skills.

### Patient behaviours

#### Adherence to ART

*Project ACCEPT* [48] found a greater likelihood of using HIV medications over time in the intervention group compared to the control group (OR 2.33, 95%CI 1.29 to 4.21). However, they found little to no difference between groups over time in terms of the self-reported adherence questionnaire. *Multisystemic Therapy* [50] found little to no difference in the rate of change in ART adherence between groups.

#### Healthcare utilisation

*Project ACCEPT* [48] found little to no difference between groups over time in terms of appointment adherence and number of medical visits.

None of the included studies reported on sexual risk behaviour, self-care abilities (substance use), symptom management or retention in care.

### Health status

#### Viral suppression

*Project ACCEPT* and *Multisystemic Therapy* [48, 50] found a decrease in viral load over time in the intervention group compared to the control group.

#### CD4 count

Both studies [48, 50] found little to no difference in CD4 count over time between groups.

#### Quality of life

*Project ACCEPT* [48] found little to no difference between groups over time.

Mental/psychological health: One study, *Project ACCEPT* [48], found little to no difference in psychological distress between groups over time.

None of the included studies reported on emotional or physical health.

### Impact

None of the included studies reported on hospitalisation, co-morbidities, all-cause mortality, HIV transmission or employment.

## Discussion

This systematic review evaluated the effectiveness of self-management interventions for improving health-related outcomes of ALHIV and aimed to identify intervention components that are effective, particularly in low-resource settings with a high HIV burden.

We included 14 studies in this review. Although we planned to include adolescents aged 10–19, most studies included young people up to 24 years and only one study reported stratified data. Interventions were heterogeneous, although the self-management components as depicted in the logic model (Fig. 1) could be identified. Most of the interventions addressed at least two self-management domains, with self-regulation the most often targeted. Interventions were primarily delivered by trained counsellors via face-to-face individual education/counselling sessions in healthcare settings. Intervention duration was between two and 8 months and the longest follow-up was 12 months. Few studies (*n* = 4) were conducted in low-resource settings, although we identified three ongoing studies that are being conducted in Africa. Interventions in a low-resource setting such as Africa (*Vuka Family Programme*; *Sauti Ya Vijana*, *Peer-led Trauma Informed CBT*, and *Stepping Stones*) predominantly used peers or lay healthcare workers as delivery agents and used group education/counselling, which may be more relevant in low-resource high HIV burden settings.

We generally found little to no difference in patient reported, behavioural and health outcomes across time, irrespective of the number of components addressed or the comparison. However, positive trends in the expected direction were observed. Variations in the definitions and imprecise measurement of patient-reported outcomes may have contributed to studies not showing an effect between groups. Furthermore, outcomes such as self-efficacy require continuous counselling [23] and follow-up periods might have been inadequate. We found small effects for adherence and viral suppression at the six, nine and 12-month follow-ups.

Although we observed clinical heterogeneity – linked to interventions, participants and outcome measurement – findings were strikingly consistent across studies. We downgraded the evidence to very low certainty for most of the key outcomes due to imprecision (wide confidence intervals and small sample sizes); indirectness as most studies did not specifically include adolescents aged 10–19; and study limitations due to concerns about risk of bias across studies.

We also did not find any specific trends with regards to the number of self-management components (domains) addressed, types of interventions (e.g. individual vs group), the delivery method (e.g. face-to-face vs ICT) or the delivery agent (healthcare worker, peer or trained counsellor) that appeared to be more effective for certain outcomes. For example, *Cell Phone Support* increased adherence and viral suppression and reduced substance use and perceived stress. The peer-delivered mental health intervention, *Sauti ya Vijana* [43, 44]; *Positive Steps*, an individual technology-based intervention [51]; and *Stepping Stones*, a group-based intervention [47], all reported increased adherence in the intervention groups compared to the control groups. The *Healthy Choices* intervention [52] found a decrease in viral load and *Sauti ya Vijana* [43, 44] reported an increase in CD4. Our findings suggest that the V*uka Family Programme* [42] was more effective than the *iPhone Game* [56] for increasing social support. However, the perception of support may differ as the *Vuka Family Programme* focused on pre-adolescents whereas the *iPhone Game* targeted older adolescents. Studies that specifically focused on addressing psychological and patient-reported outcomes, for example *Mindfulness-Based Stress Reduction* [55], may be more appropriate to improve outcomes such as mindfulness and quality of life. Another explanation for not identifying specific effective components across studies may be that many interventions used combinations of delivery methods and adjusted the intervention to the context. It, therefore, appears that interventions for ALHIV should be tailored to the individual (specifically at the developmental stage), social and health system contexts, and the specific self-management abilities and outcomes targeted.

To our knowledge, this is the first systematic review on the effectiveness of self-management interventions for ALHIV. Existing systematic reviews evaluating a variety of self-management interventions focussing on adults living with HIV reported improvements in most self-management outcomes including physical, psychosocial, health knowledge and behavioural outcomes [26, 27]. Abera et al. (2020) found that a combination of self-management interventions including skills training, phone counselling using manuals and technology-assisted interventions (phone and web-based) generally improved outcomes, especially adherence, quality of life and symptom management. Peer-based skills interventions were found to likely improve psychological outcomes and quality of life, but less so for behaviour and physical outcomes [23].

Other reviews specifically focused on the effectiveness of self-management interventions using m-health or ICT. Cooper et al. (2017) found that m-health interventions for self-management were predominantly delivered through SMS and that it affected adherence, viral load, mental health and social support [68], whereas Tufts et al. (2015) reported that m-health interventions for African-American women were mostly still exploratory and focused on adherence only [28]. In their review on communication technologies in self-management, Zhang and Li (2017) recommended that more research is needed to explore ICT interventions amongst people from low socio-economic backgrounds and low-resource settings [29]. Similarly, our findings indicate that *Cell Phone Support* [40, 41], SMS reminders from *UCare4Life* [49] and *Positive Steps* (that used SMS as the first step) [51] were m-health/ICT interventions used most often. All these studies were conducted in the USA. Only one study used a gaming platform [56]. Although our review suggests that these interventions may improve some outcomes, there is no evidence of their effectiveness in low-resource settings and the existing evidence is very uncertain. Self-management interventions have also been used and studied in other chronic conditions. One review [25] found that self-management interventions for young people with chronic conditions were effective for medical management (disease knowledge and adherence) if they were provided individually in a clinic or home setting by a mono-disciplinary team. They found conflicting evidence regarding the effect on psychological outcomes and quality of life. Interventions focused on dealing with or coping with a chronic condition (role/emotional-management) and may be effective if provided individually through telemedicine that facilitates peer support [25]. A review by Sattoe et al. (2015) found that self-management support interventions neglected psychosocial challenges experienced by chronically ill young people [9]. Although many of the interventions in our review targeted adherence or viral suppression, they addressed multiple self-management domains. Self-regulation was addressed most frequently, while social facilitation was addressed least frequently. Self-regulation, especially coping with a stigmatised condition such as HIV, is an important component of HIV self-management for adolescents. Social facilitation and active participation in care was shown to correlate with improved health-related quality of life and adherence amongst ALHIV in South Africa [21].

We followed rigorous methods to conduct our systematic review. We used a logic model to identify and unpack various aspects of the interventions and outcomes as well as used this to pre-specify the eligibility criteria for our review. Although we included different types of self-management interventions, we classified the interventions according to the domains of the *IFSMT*, which may limit the application to other frameworks. Various strategies and behaviour change interventions can be used to enhance self-management abilities. For example, the Behaviour Change Taxonomy (BCT) uses 16 clusters to characterise interventions based on their content [69]. The *IFSMT* domain of knowledge and beliefs can be addressed by using the techniques of shaping knowledge, natural consequences and self-belief. Self-regulation can be enhanced by several BCT taxonomy components: goals and planning, feedback and monitoring, comparison of outcomes, regulation, and identity. Social facilitation can be improved by social support, comparison of behaviour, and antecedents.

Our search of the literature was comprehensive and included multiple electronic databases, trial registries and grey literature. We did not have any language restrictions, although we only found studies published in English. We assessed certainty of evidence using the GRADE approach; few of the previous systematic reviews provided a grading of the evidence. Studies included in our review were heterogenous in terms of participants, interventions, and outcomes. We were, therefore, not able to explore the impact of the intervention delivery method, agent and participant characteristics. Furthermore, most studies included participants beyond 19 years of age (young people) and did not stratify data according to age groups. This precluded subgroup analysis. We noted that some studies selected participants based on high-risk behaviour or non-adherence. It may be that self-management interventions have a greater effect if implemented amongst high-risk groups or those newly diagnosed with HIV [26].

Our review findings may be particularly important for researchers who are in the process of designing self-management interventions. Currently the evidence is too uncertain to make any recommendations for programme components that may be effective. Our review focused on assessing the effectiveness of self-management interventions and did not address questions linked to ALHIV’s perceptions and experiences of these interventions, costs, and implementation issues.

None of the included studies reported on cost-effectiveness or impact outcomes that may be used to influence policy on a larger scale. Aantjes et al. (2014) previously found that self-management intervention models have low applicability in sub-Saharan Africa as most interventions are led by health-professionals whereas peer-led models may be more sustainable in low-resource settings [70].

## Conclusion

Existing evidence on the effectiveness of self-management interventions compared to control groups for improving health-related outcomes of ALHIV is very uncertain. We, therefore, do not know whether self-management interventions for ALHIV lead to better or worse behaviour and health outcomes or whether they make no difference at all. Despite this, there is a need to support ALHIV to cope with and manage a life-long condition. Implementation of self-management interventions should take into consideration the individual, social and healthcare contexts. Interventions delivered by peers or lay healthcare workers may be more feasible and sustainable in low-resource settings with a high HIV burden.

Further rigorous studies are needed to evaluate the effectiveness of self-management interventions among ALHIV living in Africa, which has the greatest burden of HIV/AIDS. This includes research on the use of cell-phone and ICT-based interventions. Furthermore, the science of self-management would benefit if studies used a taxonomy or logic models to match intervention outcomes with intervention components, including impact outcomes such as hospitalisations, mortality, and employment, so that comparable results can be provided. Randomised controlled trials with larger sample sizes that follow participants over longer periods may improve the certainty of the evidence. A qualitative synthesis of ALHIV’s experiences of various self-management interventions will be useful to evaluate reasons for lack of effectiveness of these on patient-reported and psychological outcomes. This can help to inform the development of future interventions.

## Supplementary Information

**Additional file 1.** Prisma checklist and appendix.

**Additional file 2.** Search histories.

**Additional file 3.** Summary of ongoing studies.

**Additional file 4.** Excluded studies with reasons.

**Additional file 5.** Risk of bias tables.

**Additional file 6.** Forest plots.

## Data Availability

This systematic review is based on existing published and unpublished study reports. All data analysed during this study are included in this published article and its supplementary information files.

## Abbreviations

ALHIV: Adolescents living with HIV
ART: Antiretroviral treatment
CBAs: Controlled before-after studies
CD4: Cluster of differentiation 4
EPOC: Cochrane Effective Practice and Organisation of Care
GRADE: Grades of Recommendation, Assessment, Development and Evaluation
HIV: Human Immunodeficiency Virus
ICT: Information and Communication Technologies
NRCTs: Non-randomised controlled trials
PHIV: People living with HIV
PROSPERO: International Prospective Register of Systematic Reviews
RCTs: Randomised controlled trials
TIDier: Template for Intervention Description and Replication
WHO: World Health Organisation
