# Supplementary material for: Self-management interventions for adolescents living with HIV: a systematic review
Source: BMC Infect Dis. 2021 May 7;21:431. doi: 10.1186/s12879-021-06072-0 (PMC8105944; doi:10.1186/s12879-021-06072-0)

**Search histories**

**Self-management interventions for adolescents living with HIV**

Total: n=2743 (no duplicates removed yet)

Covidence will remove duplicates automatically and show that in the PRISMA diagram

**MEDLINE PubMed**

For the search done on 31 July 2019

Total # records: 1079

| [#16](https://www.ncbi.nlm.nih.gov/pubmed/advanced) | Search **((#15 AND #11))** | [1079](https://www.ncbi.nlm.nih.gov/pubmed/?cmd=HistorySearch&querykey=16) |
| --- | --- | --- |
| [#15](https://www.ncbi.nlm.nih.gov/pubmed/advanced) | Search **((#12 OR #13 OR #14))** | [511302](https://www.ncbi.nlm.nih.gov/pubmed/?cmd=HistorySearch&querykey=15) |
| [#14](https://www.ncbi.nlm.nih.gov/pubmed/advanced) | Search **(("Patient-Centered Care"[Mesh] OR patient-centered OR patient-centred OR patient-focused OR patient-focussed OR “patient centred” or “patient centered” OR “patient focussed” OR “patient focused”))** | [34337](https://www.ncbi.nlm.nih.gov/pubmed/?cmd=HistorySearch&querykey=14) |
| [#13](https://www.ncbi.nlm.nih.gov/pubmed/advanced) | Search **((self-management OR self-manage OR empowerment OR empower or self-care or self-help or self-efficacy OR self-regulate OR "shared decision-making" OR "shared decisionmaking" OR "shared decision making" OR "patient responsibility" OR self-responsibility OR "self responsibility" OR “patient directed” OR patient-directed or goal-setting OR “goal setting” OR “reflective thinking” OR “patient engagement” OR “patient involvement” OR “engaging patients” OR “involving patients” OR self-monitor OR “self monitor” OR self-monitoring OR self-administration OR self-directed OR “consumer involvement” OR “consumer engagement” OR “consumer management”))** | [468587](https://www.ncbi.nlm.nih.gov/pubmed/?cmd=HistorySearch&querykey=13) |
| [#12](https://www.ncbi.nlm.nih.gov/pubmed/advanced) | Search **(("Self-Management"[Mesh] OR "Self Care"[Mesh] OR "Self Efficacy"[Mesh] OR "Patient Participation"[Mesh]))** | [93139](https://www.ncbi.nlm.nih.gov/pubmed/?cmd=HistorySearch&querykey=12) |
| [#11](https://www.ncbi.nlm.nih.gov/pubmed/advanced) | Search **((#7 AND #10))** | [16128](https://www.ncbi.nlm.nih.gov/pubmed/?cmd=HistorySearch&querykey=11) |
| [#10](https://www.ncbi.nlm.nih.gov/pubmed/advanced) | Search **((#8 OR #9))** | [2649790](https://www.ncbi.nlm.nih.gov/pubmed/?cmd=HistorySearch&querykey=10) |
| [#9](https://www.ncbi.nlm.nih.gov/pubmed/advanced) | Search **(("Adolescent"[Mesh] or adolescen*))** | [2039774](https://www.ncbi.nlm.nih.gov/pubmed/?cmd=HistorySearch&querykey=9) |
| [#8](https://www.ncbi.nlm.nih.gov/pubmed/advanced) | Search **(("young adult*" or teenager* or teens or "high school" or "secondary school" or student*))** | [2614405](https://www.ncbi.nlm.nih.gov/pubmed/?cmd=HistorySearch&querykey=8) |
| [#7](https://www.ncbi.nlm.nih.gov/pubmed/advanced) | Search **((#3 AND #6))** | [121752](https://www.ncbi.nlm.nih.gov/pubmed/?cmd=HistorySearch&querykey=7) |
| [#6](https://www.ncbi.nlm.nih.gov/pubmed/advanced) | Search **((#4 OR #5))** | [259608](https://www.ncbi.nlm.nih.gov/pubmed/?cmd=HistorySearch&querykey=6) |
| [#5](https://www.ncbi.nlm.nih.gov/pubmed/advanced) | Search **(((antiretroviral agents [Mesh] OR antiretroviral therapy, highly active [Mesh])))** | [76611](https://www.ncbi.nlm.nih.gov/pubmed/?cmd=HistorySearch&querykey=5) |
| [#4](https://www.ncbi.nlm.nih.gov/pubmed/advanced) | Search **(((Antiretroviral* OR ((anti) AND (retroviral*)) OR ARV* OR ART OR "antiretroviral therapy" OR HAART OR ((highly) AND (active) AND (antiretroviral*) AND (therap*)) OR ((anti) AND (hiv)) OR ((anti) AND (acquired immunodeficiency)) OR ((anti) AND (acquired immuno-deficiency)) OR ((anti) AND (acquired immune-deficiency)) OR ((anti) AND (acquired immun*) AND (deficienc*)))))** | [249854](https://www.ncbi.nlm.nih.gov/pubmed/?cmd=HistorySearch&querykey=4) |
| [#3](https://www.ncbi.nlm.nih.gov/pubmed/advanced) | Search **((#1 OR #2))** | [412090](https://www.ncbi.nlm.nih.gov/pubmed/?cmd=HistorySearch&querykey=3) |
| [#2](https://www.ncbi.nlm.nih.gov/pubmed/advanced) | Search **(((HIV infections [MeSH] OR HIV [MeSH])))** | [304879](https://www.ncbi.nlm.nih.gov/pubmed/?cmd=HistorySearch&querykey=2) |
| [#1](https://www.ncbi.nlm.nih.gov/pubmed/advanced) | Search **(((HIV OR hiv-1 OR hiv-2* OR hiv1 OR hiv2 OR hiv infect* OR human immunodeficiency virus OR human immune deficiency virus OR human immuno-deficiency virus OR human immune-deficiency virus OR ((human immun*) AND (deficiency virus)) OR acquired immunodeficiency syndromes OR acquired immune deficiency syndrome OR acquired immuno-deficiency syndrome OR acquired immune-deficiency syndrome OR ((acquired immun*) AND (deficiency syndrome)) OR HIV/AIDS)))** | [407829](https://www.ncbi.nlm.nih.gov/pubmed/?cmd=HistorySearch&querykey=1) |

**Because this number of search records differ so much from that from the other databases, I conducted the PubMed search again on 1 Aug 2019:**

Number of search records found: n=1083, which is 4 more than the above search, so I used these results

| [#32](https://www.ncbi.nlm.nih.gov/pubmed) | Search **(#31 AND #23 AND #20 AND #8)** | [1083](https://www.ncbi.nlm.nih.gov/pubmed/?cmd=HistorySearch&querykey=32) |
| --- | --- | --- |
| [#31](https://www.ncbi.nlm.nih.gov/pubmed) | Search **(((((((((self-management OR self-manage OR empowerment OR empower or self-care or self-help or self-efficacy OR self-regulate OR "shared decision-making" OR "shared decisionmaking" OR "shared decision making" OR "patient responsibility" OR self-responsibility OR "self responsibility" OR “patient directed” OR patient-directed or goal-setting OR “goal setting” OR “reflective thinking” OR “patient engagement” OR “patient involvement” OR “engaging patients” OR “involving patients” OR self-monitor OR “self monitor” OR self-monitoring OR self-administration OR self-directed OR “consumer involvement” OR “consumer engagement” OR “consumer management”)))) OR ((patient-centered OR patient-centred OR patient-focused OR patient-focussed OR “patient centred” or “patient centered” OR “patient focussed” OR “patient focused”))) OR patient-centered care[mesh]) OR self-management[mesh]) OR self care[mesh]) OR self efficacy[mesh]) OR patient participation[mesh]** | [511405](https://www.ncbi.nlm.nih.gov/pubmed/?cmd=HistorySearch&querykey=31) |
| [#30](https://www.ncbi.nlm.nih.gov/pubmed) | Search **patient participation[mesh]** | [24187](https://www.ncbi.nlm.nih.gov/pubmed/?cmd=HistorySearch&querykey=30) |
| [#29](https://www.ncbi.nlm.nih.gov/pubmed) | Search **self efficacy[mesh]** | [18783](https://www.ncbi.nlm.nih.gov/pubmed/?cmd=HistorySearch&querykey=29) |
| [#28](https://www.ncbi.nlm.nih.gov/pubmed) | Search **self care[mesh]** | [52335](https://www.ncbi.nlm.nih.gov/pubmed/?cmd=HistorySearch&querykey=28) |
| [#27](https://www.ncbi.nlm.nih.gov/pubmed) | Search **self-management[mesh]** | [1307](https://www.ncbi.nlm.nih.gov/pubmed/?cmd=HistorySearch&querykey=27) |
| [#26](https://www.ncbi.nlm.nih.gov/pubmed) | Search **patient-centered care[mesh]** | [18468](https://www.ncbi.nlm.nih.gov/pubmed/?cmd=HistorySearch&querykey=26) |
| [#25](https://www.ncbi.nlm.nih.gov/pubmed) | Search **(patient-centered OR patient-centred OR patient-focused OR patient-focussed OR “patient centred” or “patient centered” OR “patient focussed” OR “patient focused”)** | [33760](https://www.ncbi.nlm.nih.gov/pubmed/?cmd=HistorySearch&querykey=25) |
| [#24](https://www.ncbi.nlm.nih.gov/pubmed) | Search **((self-management OR self-manage OR empowerment OR empower or self-care or self-help or self-efficacy OR self-regulate OR "shared decision-making" OR "shared decisionmaking" OR "shared decision making" OR "patient responsibility" OR self-responsibility OR "self responsibility" OR “patient directed” OR patient-directed or goal-setting OR “goal setting” OR “reflective thinking” OR “patient engagement” OR “patient involvement” OR “engaging patients” OR “involving patients” OR self-monitor OR “self monitor” OR self-monitoring OR self-administration OR self-directed OR “consumer involvement” OR “consumer engagement” OR “consumer management”))** | [468677](https://www.ncbi.nlm.nih.gov/pubmed/?cmd=HistorySearch&querykey=24) |
| [#23](https://www.ncbi.nlm.nih.gov/pubmed) | Search **((("young adult*" or teenager* or teens or "high school" or "secondary school" or student* or adolescen*))) OR Adolescent[mesh]** | [2650155](https://www.ncbi.nlm.nih.gov/pubmed/?cmd=HistorySearch&querykey=23) |
| [#22](https://www.ncbi.nlm.nih.gov/pubmed) | Search **Adolescent[mesh]** | [1947868](https://www.ncbi.nlm.nih.gov/pubmed/?cmd=HistorySearch&querykey=22) |
| [#21](https://www.ncbi.nlm.nih.gov/pubmed) | Search **("young adult*" or teenager* or teens or "high school" or "secondary school" or student* or adolescen*)** | [2650155](https://www.ncbi.nlm.nih.gov/pubmed/?cmd=HistorySearch&querykey=21) |
| [#20](https://www.ncbi.nlm.nih.gov/pubmed) | Search **((((((((((Antiretroviral*) OR ((anti AND retroviral*))) OR ((ARV* OR ART OR "antiretroviral therapy" OR HAART))) OR ((highly AND active AND antiretroviral* AND therap*))) OR ((anti AND hiv))) OR ((anti AND acquired immunodeficiency))) OR ((anti AND acquired immuno-deficiency))) OR ((anti AND acquired immune-deficiency))) OR ((anti AND acquired immun* AND deficienc*))) OR Anti-Retroviral Agents[mesh]) OR Antiretroviral Therapy, Highly Active[mesh]** | [259659](https://www.ncbi.nlm.nih.gov/pubmed/?cmd=HistorySearch&querykey=20) |
| [#19](https://www.ncbi.nlm.nih.gov/pubmed) | Search **Antiretroviral Therapy, Highly Active[mesh]** | [20403](https://www.ncbi.nlm.nih.gov/pubmed/?cmd=HistorySearch&querykey=19) |
| [#18](https://www.ncbi.nlm.nih.gov/pubmed) | Search **Anti-Retroviral Agents[mesh]** | [65237](https://www.ncbi.nlm.nih.gov/pubmed/?cmd=HistorySearch&querykey=18) |
| [#17](https://www.ncbi.nlm.nih.gov/pubmed) | Search **(anti AND acquired immun* AND deficienc*)** | [926](https://www.ncbi.nlm.nih.gov/pubmed/?cmd=HistorySearch&querykey=17) |
| [#16](https://www.ncbi.nlm.nih.gov/pubmed) | Search **(anti AND acquired immune-deficiency)** | [9666](https://www.ncbi.nlm.nih.gov/pubmed/?cmd=HistorySearch&querykey=16) |
| [#15](https://www.ncbi.nlm.nih.gov/pubmed) | Search **(anti AND acquired immuno-deficiency)** | [9195](https://www.ncbi.nlm.nih.gov/pubmed/?cmd=HistorySearch&querykey=15) |
| [#14](https://www.ncbi.nlm.nih.gov/pubmed) | Search **(anti AND acquired immunodeficiency)** | [10580](https://www.ncbi.nlm.nih.gov/pubmed/?cmd=HistorySearch&querykey=14) |
| [#13](https://www.ncbi.nlm.nih.gov/pubmed) | Search **(anti AND hiv)** | [77223](https://www.ncbi.nlm.nih.gov/pubmed/?cmd=HistorySearch&querykey=13) |
| [#12](https://www.ncbi.nlm.nih.gov/pubmed) | Search **(highly AND active AND antiretroviral* AND therap*)** | [11240](https://www.ncbi.nlm.nih.gov/pubmed/?cmd=HistorySearch&querykey=12) |
| [#11](https://www.ncbi.nlm.nih.gov/pubmed) | Search **(ARV* OR ART OR "antiretroviral therapy" OR HAART)** | [188566](https://www.ncbi.nlm.nih.gov/pubmed/?cmd=HistorySearch&querykey=11) |
| [#10](https://www.ncbi.nlm.nih.gov/pubmed) | Search **(anti AND retroviral*)** | [14513](https://www.ncbi.nlm.nih.gov/pubmed/?cmd=HistorySearch&querykey=10) |
| [#9](https://www.ncbi.nlm.nih.gov/pubmed) | Search **Antiretroviral*** | [64617](https://www.ncbi.nlm.nih.gov/pubmed/?cmd=HistorySearch&querykey=9) |
| [#8](https://www.ncbi.nlm.nih.gov/pubmed) | Search **((((((((HIV OR hiv-1 OR hiv-2* OR hiv1 OR hiv2 OR hiv infect* OR human immunodeficiency virus OR human immune deficiency virus OR human immuno-deficiency virus OR human immune-deficiency virus))) OR ((((human immun*) AND (deficiency virus))))) OR ((acquired immunodeficiency syndromes OR acquired immune deficiency syndrome OR acquired immuno-deficiency syndrome OR acquired immune-deficiency syndrome))) OR ((((acquired immun*) AND (deficiency syndrome))))) OR AIDS) OR HIV infections[mesh]) OR hiv[mesh]** | [473969](https://www.ncbi.nlm.nih.gov/pubmed/?cmd=HistorySearch&querykey=8) |
| [#7](https://www.ncbi.nlm.nih.gov/pubmed) | Search **hiv[mesh]** | [95493](https://www.ncbi.nlm.nih.gov/pubmed/?cmd=HistorySearch&querykey=7) |
| [#6](https://www.ncbi.nlm.nih.gov/pubmed) | Search **HIV infections[mesh]** | [272166](https://www.ncbi.nlm.nih.gov/pubmed/?cmd=HistorySearch&querykey=6) |
| [#5](https://www.ncbi.nlm.nih.gov/pubmed) | Search **AIDS** | [272609](https://www.ncbi.nlm.nih.gov/pubmed/?cmd=HistorySearch&querykey=5) |
| [#4](https://www.ncbi.nlm.nih.gov/pubmed) | Search **(((acquired immun*) AND (deficiency syndrome)))** | [7306](https://www.ncbi.nlm.nih.gov/pubmed/?cmd=HistorySearch&querykey=4) |
| [#3](https://www.ncbi.nlm.nih.gov/pubmed) | Search **(acquired immunodeficiency syndromes OR acquired immune deficiency syndrome OR acquired immuno-deficiency syndrome OR acquired immune-deficiency syndrome)** | [91968](https://www.ncbi.nlm.nih.gov/pubmed/?cmd=HistorySearch&querykey=3) |
| [#2](https://www.ncbi.nlm.nih.gov/pubmed) | Search **(((human immun*) AND (deficiency virus)))** | [4512](https://www.ncbi.nlm.nih.gov/pubmed/?cmd=HistorySearch&querykey=2) |
| [#1](https://www.ncbi.nlm.nih.gov/pubmed) | Search **(HIV OR hiv-1 OR hiv-2* OR hiv1 OR hiv2 OR hiv infect* OR human immunodeficiency virus OR human immune deficiency virus OR human immuno-deficiency virus OR human immune-deficiency virus)** | [372445](https://www.ncbi.nlm.nih.gov/pubmed/?cmd=HistorySearch&querykey=1) |

**EMBASE (Ovid)**

Search date: 31 July 2019

# of search records: 219

Database: Embase 1947-Present, updated daily

Search Strategy:

--------------------------------------------------------------------------------

1 *Human immunodeficiency virus/ (49972)

2 *Human immunodeficiency virus infection/ (173424)

3 (human immunodeficiency virus or human immune deficiency virus or human immuno-deficiency virus or human immune-deficiency virus).ab. (79773)

4 (human immunodeficiency virus or human immune deficiency virus or human immuno-deficiency virus or human immune-deficiency virus).ti. (35254)

5 (hiv-1* or hiv-2* or hiv1 or hiv2).ti. or (hiv-1* or hiv-2* or hiv1 or hiv2).ab. (95744)

6 aids.mp. or acquired immune deficiency syndrome/ (235219)

7 (acquired immun* and deficiency syndrome).ti. or (acquired immun* and deficiency syndrome).ab. (6296)

8 1 or 2 or 3 or 4 or 5 or 6 or 7 (456061)

9 highly active antiretroviral therapy/ or antiretroviral therapy/ or antiretrovirals.mp. (50913)

10 (Antiretroviral* or (anti and retroviral*) or ARV* or ART or "antiretroviral therapy" or HAART or (highly and active and antiretroviral* and therap*) or (anti and hiv) or (anti and acquired immunodeficiency) or (anti and acquired immuno-deficiency) or (anti and acquired immune-deficiency) or (anti and acquired immun* and deficienc*)).mp. (301619)

11 9 or 10 (301619)

12 8 and 11 (112514)

13 ("young adult*" or teenager* or teens or "high school" or "secondary school" or student*).mp. [mp=title, abstract, heading word, drug trade name, original title, device manufacturer, drug manufacturer, device trade name, keyword, floating subheading word, candidate term word] (888217)

14 adolescent/ (1588677)

15 13 or 14 (2264216)

16 12 and 15 (8934)

17 self management.mp. or self care/ (61315)

18 self efficacy.mp. or self concept/ (98457)

19 patient participation.mp. or patient participation/ (27229)

20 (empowerment or empower or self-help or self-efficacy or self-regulate or "shared decision-making" or "shared decisionmaking" or "shared decision making").mp. [mp=title, abstract, heading word, drug trade name, original title, device manufacturer, drug manufacturer, device trade name, keyword, floating subheading word, candidate term word] (82403)

21 ("patient responsibility" or self-responsibility or "self responsibility" or "patient directed" or patient-directed or goal-setting or "goal setting" or "reflective thinking").mp. [mp=title, abstract, heading word, drug trade name, original title, device manufacturer, drug manufacturer, device trade name, keyword, floating subheading word, candidate term word] (6488)

22 ("patient engagement" or "patient involvement" or self-monitor or "self monitor" or self-monitoring).mp. [mp=title, abstract, heading word, drug trade name, original title, device manufacturer, drug manufacturer, device trade name, keyword, floating subheading word, candidate term word] (18938)

23 (self-administration or self-directed or "consumer involvement" or "consumer engagement" or "consumer management").mp. [mp=title, abstract, heading word, drug trade name, original title, device manufacturer, drug manufacturer, device trade name, keyword, floating subheading word, candidate term word] (23964)

24 patient centered care.mp. (5909)

25 (patient-focused or patient-focussed or "patient centred" or "patient centered" or "patient focussed" or "patient focused").mp. [mp=title, abstract, heading word, drug trade name, original title, device manufacturer, drug manufacturer, device trade name, keyword, floating subheading word, candidate term word] (30579)

26 17 or 18 or 19 or 20 or 21 or 22 or 23 or 24 or 25 (288978)

27 youth.mp. or juvenile/ (102097)

28 young adult/ (302475)

29 exp juvenile/ (3751778)

30 13 or 14 or 27 or 28 or 29 (4376259)

31 12 and 30 (15241)

32 26 and 31 (219)

**CENTRAL (Cochrane Library)**

Total # records: 56

Date Run: 31/07/2019 17:24:55

ID Search Hits

#1 HIV OR hiv-1 OR hiv-2* OR hiv1 OR hiv2 OR hiv infect* OR human immunodeficiency virus OR human immune deficiency virus OR human immuno-deficiency virus OR human immune-deficiency virus 26056

#2 ((human immun*) AND (deficiency virus)) 1698

#3 acquired immunodeficiency syndromes OR acquired immune deficiency syndrome OR acquired immuno-deficiency syndrome OR acquired immune-deficiency syndrome 2095

#4 ((acquired immun*) AND (deficiency syndrome)) 1745

#5 AIDS 13626

#6 MeSH descriptor: [HIV Infections] explode all trees 11221

#7 MeSH descriptor: [HIV] explode all trees 2970

#8 #1 OR #2 OR #3 OR #4 OR #5 OR #6 OR #7 30874

#9 Antiretroviral* 8616

#10 anti AND retroviral* 1015

#11 ARV* OR ART OR "antiretroviral therapy" OR HAART 23424

#12 highly AND active AND antiretroviral* AND therap* 2380

#13 anti AND hiv 5641

#14 anti AND acquired immunodeficiency 695

#15 anti AND acquired immuno-deficiency 72

#16 anti AND acquired immune-deficiency 638

#17 anti AND acquired immun* AND deficienc* 751

#18 MeSH descriptor: [Anti-Retroviral Agents] explode all trees 4161

#19 MeSH descriptor: [Antiretroviral Therapy, Highly Active] explode all trees 1157

#20 #9 OR #10 OR #11 OR #12 OR #13 OR #14 OR #15 OR #16 OR #17 OR #18 OR #19 28108

#21 #8 AND #20 12362

#22 "young adult*" or teenager* or teens or "high school" or "secondary school" or student* or adolescen* 198305

#23 MeSH descriptor: [Adolescent] explode all trees 100420

#24 #22 OR #23 198306

#25 #21 AND #24 2132

#26 (self-management OR self-manage OR empowerment OR empower or self-care or self-help or self-efficacy OR self-regulate OR "shared decision-making" OR "shared decisionmaking" OR "shared decision making" OR "patient responsibility" OR self-responsibility OR "self responsibility" OR “patient directed” OR patient-directed or goal-setting OR “goal setting” OR “reflective thinking” OR “patient engagement” OR “patient involvement” OR “engaging patients” OR “involving patients” OR self-monitor OR “self monitor” OR self-monitoring OR self-administration OR self-directed OR “consumer involvement” OR “consumer engagement” OR “consumer management”) 34069

#27 patient-centered OR patient-centred OR patient-focused OR patient-focussed OR “patient centred” or “patient centered” OR “patient focussed” OR “patient focused” 3441

#28 MeSH descriptor: [Patient-Centered Care] explode all trees 625

#29 MeSH descriptor: [Self-Management] explode all trees 209

#30 MeSH descriptor: [Self Care] explode all trees 5304

#31 MeSH descriptor: [Self Efficacy] explode all trees 2845

#32 MeSH descriptor: [Patient Participation] explode all trees 1221

#33 #26 OR #27 OR #28 OR #29 OR #30 OR #31 OR #32 37396

#34 #25 AND #33 in Trials 56

**Africa-Wide Information (EBSCOhost)**

Search done on 1 Aug 2019

Total # records: 173

S1 TX ( HIV OR hiv-1 OR hiv-2* OR hiv1 OR hiv2 OR hiv infect* OR human immunodeficiency virus OR human immune deficiency virus OR human immuno-deficiency virus OR human immune-deficiency virus ) OR TX ( ((human immun*) AND (deficiency virus)) ) OR TX ( acquired immunodeficiency syndromes OR acquired immune deficiency syndrome OR acquired immuno-deficiency syndrome OR acquired immune-deficiency syndrome ) OR TX ( ((acquired immun*) AND (deficiency syndrome)) ) OR TX ( AIDS OR HIV/aids ) OR SM hiv infection OR SM hiv [140,235]

S2 TX Antiretroviral* OR TX ( anti AND retroviral* ) OR TX ( ARV* OR ART OR "antiretroviral therapy" OR HAART ) OR TX ( highly AND active AND antiretroviral* AND therap* ) OR TX ( anti AND hiv ) OR TX ( anti AND acquired immunodeficiency ) OR TX ( anti AND acquired immuno-deficiency ) OR TX ( anti AND acquired immune-deficiency ) OR TX ( anti AND acquired immun* AND deficienc* ) OR SM antiretroviral OR SM antiretroviral therapy  [95,345]

S3 TX ( "young adult*" or teenager* or teens or "high school" or "secondary school" or student* or adolescen* ) OR SM adolescents [162,434]

S4 TX ( (self-management OR self-manage OR empowerment OR empower or self-care or self-help or self-efficacy OR self-regulate OR "shared decision-making" OR "shared decisionmaking" OR "shared decision making" OR "patient responsibility" OR self-responsibility OR "self responsibility" OR “patient directed” OR patient-directed or goal-setting OR “goal setting” OR “reflective thinking” OR “patient engagement” OR “patient involvement” OR “engaging patients” OR “involving patients” OR self-monitor OR “self monitor” OR self-monitoring OR self-administration OR self-directed OR “consumer involvement” OR “consumer engagement” OR “consumer management”) ) OR TX ( patient-centered OR patient-centred OR patient-focused OR patient-focussed OR “patient centred” or “patient centered” OR “patient focussed” OR “patient focused” ) OR SM patient centered care OR SM self management OR SM self care OR SM self-efficacy OR SM patient participation [28,992]

S5 S1 AND S2 AND S3 AND S4  [173]

**CINAHL (EBSCOhost)**

Search done on 1 Aug 2019

Total # records: 234

S1 TX ( HIV OR hiv-1 OR hiv-2* OR hiv1 OR hiv2 OR hiv infect* OR human immunodeficiency virus OR human immune deficiency virus OR human immuno-deficiency virus OR human immune-deficiency virus ) OR TX ( ((human immun*) AND (deficiency virus)) ) OR TX ( acquired immunodeficiency syndromes OR acquired immune deficiency syndrome OR acquired immuno-deficiency syndrome OR acquired immune-deficiency syndrome ) OR TX ( ((acquired immun*) AND (deficiency syndrome)) ) OR TX ( AIDS OR hiv/aids ) OR MW hiv infection OR MW hiv [144,654]

S2 TX Antiretroviral* OR TX ( anti AND retroviral* ) OR TX ( ARV* OR ART OR "antiretroviral therapy" OR HAART ) OR TX ( highly AND active AND antiretroviral* AND therap* ) OR TX ( anti AND hiv ) OR TX ( anti AND acquired immunodeficiency ) OR TX ( anti AND acquired immuno-deficiency ) OR TX ( anti AND acquired immune-deficiency ) OR TX ( anti AND acquired immun* AND deficienc* ) OR MW antiretroviral OR MW antiretroviral therapy [79,534]

S3 TX ( "young adult*" or teenager* or teens or "high school" or "secondary school" or student* or adolescen* ) OR MW adolescents [807,657]

S4 TX ( (self-management OR self-manage OR empowerment OR empower or self-care or self-help or self-efficacy OR self-regulate OR "shared decision-making" OR "shared decisionmaking" OR "shared decision making" OR "patient responsibility" OR self-responsibility OR "self responsibility" OR “patient directed” OR patient-directed or goal-setting OR “goal setting” OR “reflective thinking” OR “patient engagement” OR “patient involvement” OR “engaging patients” OR “involving patients” OR self-monitor OR “self monitor” OR self-monitoring OR self-administration OR self-directed OR “consumer involvement” OR “consumer engagement” OR “consumer management”) ) OR TX ( patient-centered OR patient-centred OR patient-focused OR patient-focussed OR “patient centred” or “patient centered” OR “patient focussed” OR “patient focused” ) OR MW patient centered care OR MW self management OR MW self care OR MW self-efficacy OR MW patient participation [153,376]

S5 S1 AND S2 AND S3 AND S4  [234]

**Web of Science Core Collection: SCI-EXPANDED, CPCI-S, SSCI (Clarivate Analytics)**

Search done on 1 Aug 2019

Total # records: 321

#1 TS=(HIV OR hiv-1 OR hiv-2* OR hiv1 OR hiv2 OR hiv infect* OR human immunodeficiency virus OR human immune deficiency virus OR human immuno-deficiency virus OR human immune-deficiency virus) OR TS=(((human immun*) AND (deficiency virus))) OR TS=(acquired immunodeficiency syndromes OR acquired immune deficiency syndrome OR acquired immuno-deficiency syndrome OR acquired immune-deficiency syndrome) OR TS=(((acquired immun*) AND (deficiency syndrome))) OR TS=(aids) OR TS=(HIV infections) OR TS=(HIV)

*Indexes=SCI-EXPANDED, SSCI, CPCI-S Timespan=All years*

[803,652]

#2 TS=(Antiretroviral*) OR TS=(anti AND retroviral*) OR TS=(ARV* OR ART OR "antiretroviral therapy" OR HAART) OR TS=(highly AND active AND antiretroviral* AND therap*) OR TS=(anti AND hiv) OR TS=(anti AND acquired immunodeficiency) OR TS=(anti AND acquired immuno-deficiency) OR TS=(anti AND acquired immune-deficiency) OR TS=(anti AND acquired immun* AND deficienc*) OR TS=(antiretroviral) OR TS=(antiretroviral therapy)

*Indexes=SCI-EXPANDED, SSCI, CPCI-S Timespan=All years*

[461,664]]

#3 TS=("young adult*" or teenager* or teens or "high school" or "secondary school" or student* or adolescen*) OR TS=(adolescent)

*Indexes=SCI-EXPANDED, SSCI, CPCI-S Timespan=All years*

*[999,221]*

#4 TS=((self-management OR self-manage OR empowerment OR empower or self-care or self-help or self-efficacy OR self-regulate OR "shared decision-making" OR "shared decisionmaking" OR "shared decision making" OR "patient responsibility" OR self-responsibility OR "self responsibility" OR “patient directed” OR patient-directed or goal-setting OR “goal setting” OR “reflective thinking” OR “patient engagement” OR “patient involvement” OR “engaging patients” OR “involving patients” OR self-monitor OR “self monitor” OR self-monitoring OR self-administration OR self-directed OR “consumer involvement” OR “consumer engagement” OR “consumer management”)) OR ALL=(patient-centered OR patient-centred OR patient-focused OR patient-focussed OR “patient centred” or “patient centered” OR “patient focussed” OR “patient focused”) OR TS=(Patient Centered Care) OR TS=(self management) OR TS=(self care) OR TS=(self efficacy) OR TS=(patient participation)

*Indexes=SCI-EXPANDED, SSCI, CPCI-S Timespan=All years*

[455,433]

#5 #4 AND #3 AND #2 AND #1

*Indexes=SCI-EXPANDED, SSCI, CPCI-S Timespan=All years*

[321]

**LILACS (Virtual Health Library)**

Searched on 1 Aug 2019

# of search records: n=29

| Database : | **LILACS** |
| --- | --- |
| Search on : | **self management OR self manage OR empowerment OR empower or self care or self help or self efficacy or patient participation [Words] and HIV OR AIDS OR antiretroviral$ OR HAART OR ART [Words] and adolescent$ OR adolescence OR teenager$ OR young adult$ OR teens OR high school OR student$ OR secondary school [Words]** |

**ClinicalTrials.gov** ([www.ClinicalTrials.gov](http://www.ClinicalTrials.gov))

Search date: 1 Aug 2019

# of search records: n=289

Top of Form

1008 Studies found for: **adolescent* OR adolescence OR teenager* OR young adult* OR teens OR high school OR student* OR secondary school | HIV OR AIDS OR antiretroviral$ OR HAART OR ART**

Bottom of Form

So I did not use these

But instead used the following:

289 Studies found for: **Self management OR self manage OR empowerment OR empower or self care or self help or self efficacy or patient participation | HIV OR AIDS OR antiretroviral OR HAART OR ART**

**World Health Organization (WHO) trials portal** (<http://apps.who.int/trialsearch/>)

Search date: 1 Aug 2019

# of search records: n=346 records for 339 trials found!

Advanced Search:

(HIV OR AIDS OR antiretroviral OR HAART OR ART) in the Condition

(Self management OR self manage OR empowerment OR empower or self care or self help or self efficacy or patient participation) in the Intervention

Recruitment status is ALL

 
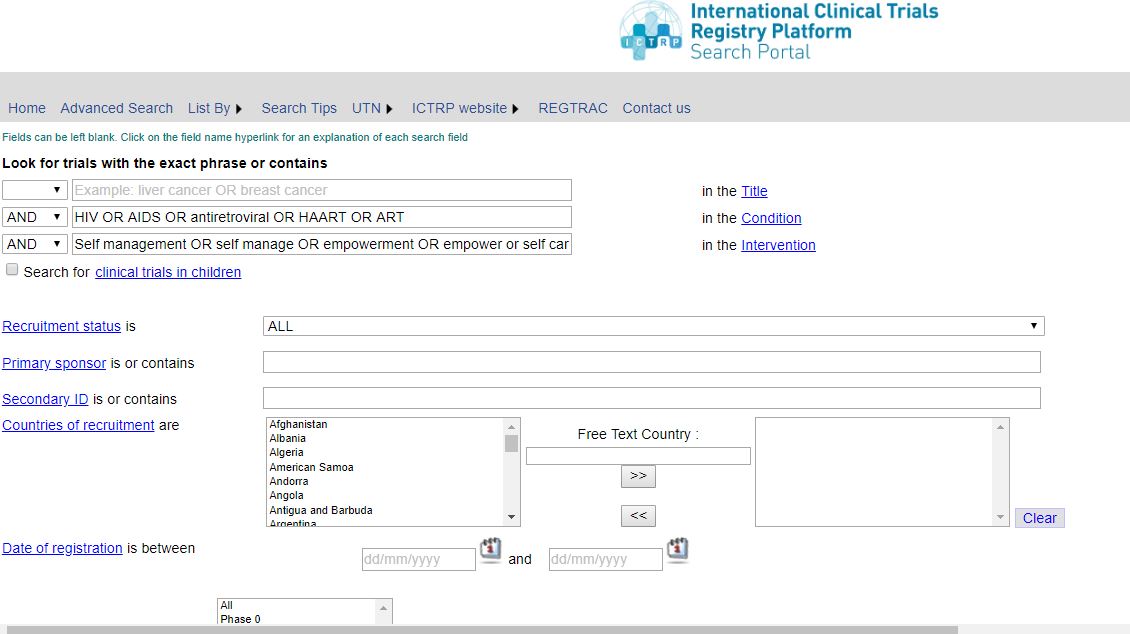

Supplement: Supplementary file 2 — Additional file 2. Search histories. [file 12879_2021_6072_MOESM2_ESM.docx]
