# Supplementary material for: Self-management interventions for adolescents living with HIV: a systematic review
Source: BMC Infect Dis. 2021 May 7;21:431. doi: 10.1186/s12879-021-06072-0 (PMC8105944; doi:10.1186/s12879-021-06072-0)
Supplement: Supplementary file 3 — Additional file 3. Summary of ongoing studies. [file 12879_2021_6072_MOESM3_ESM.docx]

Additional file 3 Summary of ongoing studies

| **ID** | **Name of intervention** | **Design** | **Age group** | **Estimated sample size** | **Country & Setting** | **Outcomes** |
| --- | --- | --- | --- | --- | --- | --- |
| Agwu (2018) | Tech2Check - technology-enhanced community health nursing intervention | RCT – parallel group | 12-25 | 120 | USA  High income  Urban  Home/community | **Viral suppression**  **Health care utilisation**  **Management cost difference** |
| Amico (2019) | TERA (Triggered Escalating Real-Time Adherence) | RCT – parallel group | 13-24 | 120 | USA  High income  Urban  ICT | **HIV stigma mechanisms**  **Confidence**  **Social support**  **Adherence**e  **Health related quality of life**  **Mental health** – Depression, life events  **Emotional health** – Emotional regulation  **Health/risk behaviours -** Sex behavior  **Self-care abilities** - Substance use  **Viral suppression**  **Services utilisation** |
| Belzer (2018)  Naar (2019) | Text message/Cell Phone support (SMART)/Scale-it-Up programme | RCT – parallel group | 15-24 | 190 | USA  High income  Urban  Home | **Adherence**  **Viral suppression**  **Self-care abilities** - Substance use  **Mental Health** - Depression |
| Donenberg (2019) | IMPAACT – Trauma informed CBT | RCT – parallel group | 15-19 | 256 (128 youth and caregivers per arm) | Botswana, South Africa, Zimbabwe and Malawi  Middle to low income  Urban/rural  Health facility | **Mental Health** – Anxiety, post traumatic stress  **Adherence**  **Viral suppression** |
| Horvath (2019)  Amico (2017) | YouThrive | RCT – parallel group | 15-24 | 300 (150 per group) | USA  High income  Urban  Home | **Confidence -** Adherence self-efficacy and adherence support  **HIV stigma**  **Social support**  **Adherence**  **Viral suppression**  **Health care utilisation**  **Health/risk behaviour** – sexual behaviour and relationship status  **Self-care abilities** – substance use  **Mental health**  **Emotional health** – emotional regulation  **Technology adoption and use** |
| Mimiaga (2018) | Positive STEPS | RCT – parallel group | 16-29 | 192 (96 per group) | USA  High Income  Urban  Health facility | **Adherence**  **Viral suppression**  **Secondary outcomes:** social support, self-efficacy, self-regulation, and motivation / outcome expectancies |
| Outlaw (2016) | Motivational Enhancement System for Adherence (MESA) | RCT – parallel group | 16-24 | 200 | USA  High income  Urban  Home/ICT | **Knowledge –** HIV treatment knowledge  **Confidence**  - Motivation, behavioral skills/confidence to take medication  **Social support**  **Adherence**  **Viral suppression**  **CD 4 count**  **Self-care abilities** – substance use  **Mental health** |
| Rotheram 2019 Arnold 2019 | Stepped Care intervention | RCT – parallel group | 12-24 | 220 (110 per group) | USA  High income  Urban  Home & Health facility | **Adherence**  **Viral suppression**  **Health care utilisation**  **Health/risk behaviour** – sexual behaviour  **Self-care abilities** - alcohol use and substance use  **Mental health**  **Health care costing** |
| Sam-Agudu (2017)^^[[1]](#footnote-1)^^ | Adolescent Coordinated Transition | Cluster RCT – parallel group | 13-17 | 276 | Nigeria  Middle income  Urban/rural  Health facility | **Social support**  **Mental health**  **Viral suppression**  **Health/risk behaviours** - Sexual risk behavior  **Transition Readiness^^[[2]](#footnote-2)^^** |
| Sibinga (2018) | Mindfulness-based stress reduction (MBSR) | RCT – parallel group | 13-24 | 170 (85 per group) | USA  High income  Urban  Health facility | **Knowledge**  - illness cognition  **Mindfulness**  **Mental health**.  **Emotional health**  **Adherence**  **Health/risk behaviour** – sex behaviour  **Health care utilisation** |
| Subramanian (2019) ^^[[3]](#footnote-3)^^ | Integrated Care Delivery of HIV Prevention and Treatment (SHIELD - Support for HIV Integrated Education, Linkages to Care, and  Destigmatization) | Cluster RCT – parallel group | HIV cohort: 16-24 | HIV cohort: 160-170 | Zambia  Low income  Urban  Health facility (unclear) | **Adherence**  **Viral suppression**  **Health care utilisation** |

1. According to the main author, this project was not completed [↑](#footnote-ref-1)
2. Not part of the outcomes of this review, but included for completeness [↑](#footnote-ref-2)
3. This study has a HIV negative and an HIV positive cohort – the interventions aspects presented here are for the HIV positive cohort [↑](#footnote-ref-3)
