# Supplementary material for: Self-management interventions for adolescents living with HIV: a systematic review
Source: BMC Infect Dis. 2021 May 7;21:431. doi: 10.1186/s12879-021-06072-0 (PMC8105944; doi:10.1186/s12879-021-06072-0)
Supplement: Supplementary file 4 — Additional file 4. Excluded studies with reasons. [file 12879_2021_6072_MOESM4_ESM.docx]

Additional file 4: Excluded studies and reasons

| **ID** | **Ongoing/Complete** | **Reason for exclusion** | **Name of intervention** |
| --- | --- | --- | --- |
| Ampt et al. (2017) (1) | Ongoing | Wrong patient population | WHISPER (text messaging) |
| Berrien et al. (2004) (2) | Complete | Wrong intervention | Home-based Nursing |
| Blashill et al. (2017) (3) | Complete | Wrong patient population | CBT for body-image and self-care |
| Chang et al. (2017) (4) | Ongoing | Wrong patient population | Community Health Worker |
| Christopoulos et al. (2014, 2018) (5,6) | Complete | Wrong patient population | Connect 4 Care (2-way text messaging) |
| Ssewamala (2018) (7) | Ongoing | Wrong intervention | Youth focused economic empowerment |
| Dilorio et al. (2008) (8) | Complete | Wrong patient population | Motivational interviewing |
| Fuller & Robinson (2020) (9) | Ongoing | Wrong study design | Plus Care mobile platform (case management) |
| Pinto (2017) (10) | Ongoing | Wrong patient population | Computer-based intervention for HIV+YoungAdults (eSMART-MH) |
| Garofalo et al. (2017) (11) | Complete | Wrong intervention | Personalized text message reminders |
| Graves et al. (2018) (12) | Complete | Wrong intervention | Family day clinic |
| Tu et al. (2011) (13) | Complete | Wrong patient population | Community-driven Self-Management support intervention (coaching) |
| Hailey & Arscott (2013) (14) | Complete | Wrong study design | STAR Track adherence programme (ST-AMP) - text messaging; online DOT |
| Moghadam et al. (2018) (15) | Complete | Wrong patient population | Empowerment programme for women |
| Kaihin et al. (2015) (16) | Complete | Wrong study design | Empowerment intervention |
| Lyon et al. (2003) (17) | Complete | Wrong study design | Family group intervention |
| Zimet (2020) (18) | Ongoing | Wrong study design | Empowerment intervention |
| Naar-King et al. (2018) (19) | Ongoing | Wrong study design | Targeting prospective memory (PM)- mostly 2way tailored text messaging |
| Willis et al. (2019) (20) | Complete | Wrong intervention | Community adolescent treatment supporters |
| Chokephaibulkit et al. (2016) (21) | Complete | Wrong study design | Happy Teen Programme |
| Pardo et al. (2017) (22) | Complete | Wrong study design | CHAMP+ model |

**References**

1. Ampt FH, Mudogo C, Gichangi P, Lim MSC, Manguro G, Chersich M, et al. WHISPER or SHOUT study : protocol of a cluster-randomised controlled trial assessing mHealth sexual reproductive health and nutrition interventions among female sex workers in Mombasa , Kenya. BMJ Open. 2017; 7(8), e017388. https://doi.org/10.1136/bmjopen-2017-017388

2. Berrien V, Salazar JC, Reynolds E, McKay K. Adherence to Antiretroviral Therapy in HIV-Infected Pediatric Patients Improves with Home-Based Intensive Nursing Intervention. AIDS Patient Care STDS. 2004;18(6):355–63.

3. Blashill AJ, Safren SA, Wilhelm S, Jampel J, Taylor SW, O’Cleirigh C, et al. Cognitive Behavioral Therapy for Body Image and Self-Care (CBT-BISC) in Sexual Minority Men Living with HIV: A Randomized Controlled Trial. Heal Psychol. 2017;36(10):937–46. https://doi.org/10.1037/hea0000505

4. Chang LW, Mbabali I, Kong X, Hutton H, Amico KR, Kennedy CE, et al. Impact of a community health worker HIV treatment and prevention intervention in an HIV hotspot fishing community in Rakai , Uganda (mLAKE): study protocol for a randomized controlled trial. Trials. 2017; 18, 494. https://doi.org/10.1186/s13063-017-2243-6.

5. Christopoulos KA, Riley ED, Carrico AW, Tulsky J, Moskowitz JT, Dilworth S, et al. A Randomized Controlled Trial of a Text Messaging Intervention to Promote Virologic Suppression and Retention in Care in an Urban Safety-Net Human Immunodeficiency Virus Clinic : The Connect4Care Trial. Clinical Infectious Diseases. 2018; 67(5), 751–759. https://doi.org/10.1093/cid/ciy156

6. Christopoulos KA, Riley ED, Tulsky J, Carrico AW, Moskowitz JT, Wilson L, et al. A text messaging intervention to improve retention in care and virologic suppression in a U . S . urban safety-net HIV clinic : study protocol for the Connect4Care ( C4C ) randomized controlled trial. BMC Infect Dis, 2014; 14, 7181–11. https://doi.org/10.1186/s12879-014-0718-62014;1–11.

7. Ssewamala F. Evaluating a Youth-Focused Economic Empowerment Approach to HIV Treatment Adherence. ClinicalTrials.gov [Internet]. 2018; Available from: https://clinicaltrials.gov/ct2/show/results/NCT01790373

8. Dilorio C, McCarthy F, Resnicow K, Holstad MM, Soet J, Yeager K, et al. Using motivational interviewing to promote adherence to antiretroviral medications: A randomized controlled study C. AIDS Behav. 2008;20(3):273–83. https://doi.org/10.1080/09540120701593489

9. Fuller J, Robinson M. PlusCare: Mobile Platform to Increase Linkage to Care in Adolescents Living With HIV / AIDS. ClinicalTrials.gov [Internet]. 2020; Available from: https://clinicaltrials.gov/ct2/show/NCT03758066

10. Pinto M. Computer-Based Intervention in HIV-Positive Young Adults. ClinicalTrials.gov [Internet]. 2017; Available from: https://clinicaltrials.gov/ct2/show/NCT02544126

11. Garofalo R, Kuhns LM, Hotton A, Johnson A, Muldoon A, Rice D. A randomized controlled trial of personalized text message reminders to promote medication adherence among HIV-positive adolescents and young adults. AIDS Behav. 2017;20(5):1049–59. https://doi.org/10.1007/s10461-015-1192-x.A

12. Graves JC, Elyanu P, Schellack CJ, Asire B, Prust L, Prescott MR, et al. Impact of a Family Clinic Day intervention on paediatric and adolescent appointment adherence and retention in antiretroviral therapy : A cluster randomized controlled trial in Uganda. PLoS One [Internet]. 2018;679:1–18. Available from: http://dx.doi.org/10.1371/journal.pone.0192068

13. Tu D, Littlejohn D, Gross P, Bodenhamer S, Tam T, Pedersen S, et al. Does individual self-management support coaching increase antiretroviral adherence? Can J Infect Dis Med Microbiol. 2011; 22, 4–129..

14. Hailey JH, Arscott J. Using Technology to Effectively Engage Adolescents and Young Adults into Care : STAR TRACK Adherence Program. J Assoc Nurses AIDS Care [Internet]. 2013;24(6):582–6. Available from: http://dx.doi.org/10.1016/j.jana.2013.03.001

15. Moghadam ZB, Rezaei E, Sharifi B, Nejat S, Saeieh SE. The Effect of Empowerment and Educational Programs on the Quality of Life in Iranian Women with HIV. J Int Assoc Provid AIDS Care [Internet]. 2018;17:1–9. Available from: https://doi.org/10.1177/2325958218759681

16. Kaihin R, Kastpibal N, Chitreechuer J, Grimes RM. Effect of an Empowerment Intervention on Antiretroviral Drug Adherence in Thai Youth. Behav Med. 2015;41(4):186–94. https://doi.org/10.1080/08964289.2014.911717

17. Lyon ME, Trexler C, Akpan-Townsend C, Selden K, Fletcher J, Addlestone IC, et al. A Family Group Approach to Increasing Adherence to Therapy in HIV-Infected Youths : Results of a Pilot Project. AIDS Patient Care STDS. 2003;17(6):299–308.

18. Zimet G. Development of an Empowerment Intervention for Young Women Living With HIV. ClinicalTrials.gov [Internet]. 2020; Available from: https://clinicaltrials.gov/ct2/show/NCT01454921

19. Naar-King S, Woods SP, Outlaw AY. Targeting PM to Improve HIV Adherence in Adolescents at Risk for Substance Abuse. ClinicalTrials.gov [Internet]. 2018; Available from: https://clinicaltrials.gov/ct2/show/NCT01959217

20. Willis N, Milanzi A, Mawodzeke M, Dziwa C, Armstrong A, Yekeye I, et al. Effectiveness of community adolescent treatment supporters ( CATS ) interventions in improving linkage and retention in care , adherence to ART and psychosocial well-being: a randomised trial among adolescents living with HIV in rural Zimbabwe. BMC Publich Heal [Internet]. 2019;19:1–9. Available from: https://doi.org/10.1186/s12889-019-6447-4

21. Chokephaibulkit K, Tarugsa J, Hospital S, Lolekha R, Leowsrisook P, Manaboriboon B, et al. Outcomes of a Comprehensive Youth Program for HIV-infected Adolescents in Thailand. JANAC. 2016;26(6), 758–769. https://doi.org/10.1016/j.jana.2015.08.005..

22. Pardo G, Saisaengjan C, Gopalan P, Ananworanich J, Lakhonpon S, Nestadt DF, et al. Cultural Adaptation of an Evidence-Informed Psychosocial Intervention to Address the Needs of PHIV + Youth in Thailand. Glov Soc Welf. 2017;4:209–18. https://doi.org/10.1007/s40609-017-0100-x
