# Supplementary material for: Self-management interventions for adolescents living with HIV: a systematic review
Source: BMC Infect Dis. 2021 May 7;21:431. doi: 10.1186/s12879-021-06072-0 (PMC8105944; doi:10.1186/s12879-021-06072-0)
Supplement: Supplementary file 5 — Additional file 5. Risk of bias tables. [file 12879_2021_6072_MOESM5_ESM.docx]

# Additional file 5: Risk of bias tables

### Belzer 2015

| **Bias** | **Authors’ judgement** | **Support for judgement** |
| --- | --- | --- |
| Was the allocation sequence adequately generated? | Unclear risk | Not reported how sequence was generated |
| Was the allocation sequence adequately concealed? | Unclear risk | Not reported |
| Were baseline characteristics similar? | Low risk | Baseline characteristics similar |
| Were baseline outcome measurements similar? | Low risk | Baseline outcome measurements were similar |
| Was knowledge of the allocated interventions adequately prevented during the study? | High risk | Not able to blind participants and personnel. Might influence outcomes, as some are subjective |
| Was the study adequately protected against contamination | Unclear risk | YLHIV selected from 5 study sites and intervention involved individual phone calls. Therefore unlikely that there was contamination. However, not reported whether YLHIV from the same sites engaged in other ways |
| Were incomplete outcome data adequately addressed? | High risk | Attrition in the intervention group: 9/19 (47%) Control: 1/18 6% |
| Was the study free from selective outcome reporting? | Low risk | Outcomes pre-specified in methods section reported in 2 separate publications |
| Was the study free from other risks of bias? | Low risk |  |

### Bhana 2014

| **Bias** | **Authors’ judgement** | **Support for judgement** |
| --- | --- | --- |
| Was the allocation sequence adequately generated? | Unclear risk | not reported |
| Was the allocation sequence adequately concealed? | Unclear risk | not reported |
| Were baseline characteristics similar? | Unclear risk | Baseline characteristics not reported |
| Were baseline outcome measurements similar? | Unclear risk | Baseline outcome measurements not reported |
| Was knowledge of the allocated interventions adequately prevented during the study? | High risk | Not able to blind participants and personnel. Might influence outcomes, as some are subjective |
| Was the study adequately protected against contamination | Unclear risk | Not reported how contamination was avoided. Participants at both sites individually randomised. |
| Were incomplete outcome data adequately addressed? | Unclear risk | 74 families enrolled, only 65 randomised. Of those, 59 completed follow-up assessments (91%). Not stratified by group, no flow-chart Among the 33 families randomized to the VUKA arm, 32 attended at least one session, 100% of whom completed it, with 94% attending at least 5 of the 6 days, and 55% attending all 6 days. The most common reasons for not attending were illness and family time conflicts. |
| Was the study free from selective outcome reporting? | High risk | Not all outcomes clearly reported |
| Was the study free from other risks of bias? | Low risk |  |

### Dow 2020

| **Bias** | **Authors’ judgement** | **Support for judgement** |
| --- | --- | --- |
| Was the allocation sequence adequately generated? | Unclear risk | Coin tossing to allocate individuals. Two individuals based on alphabetical order. |
| Was the allocation sequence adequately concealed? | Unclear risk | Coin tossing was done according to an alphabetical list (which does not seem to be concealed) |
| Were baseline characteristics similar? | Low risk | No statistical test, but there seem to be no striking differences between groups |
| Were baseline outcome measurements similar? | Low risk | No statistical test for differences between baseline outcomes, but no striking differences |
| Was knowledge of the allocated interventions adequately prevented during the study? | High risk | Not able to blind personnel and participants due to the nature of the intervention. In the trial registry report they mention that the outcome assessor was unaware of group allocation, but they don't report how this was done. |
| Was the study adequately protected against contamination | Unclear risk | Not described. Siblings were randomised to same group. Control group sessions were at different times. Not stated if participants were excluded if participating in other interventions. |
| Were incomplete outcome data adequately addressed? | Unclear risk | Total loss to follow-up: 11%, LTFU in intervention: 5% vs 19% in control group |
| Was the study free from selective outcome reporting? | Low risk | Prespecified outcomes reported |
| Was the study free from other risks of bias? | Low risk | No other concerns |

### Fabri 2015

| **Bias** | **Authors’ judgement** | **Support for judgement** |
| --- | --- | --- |
| Was the allocation sequence adequately generated? | Unclear risk | no complete report available |
| Was the allocation sequence adequately concealed? | Unclear risk | no complete report available |
| Were baseline characteristics similar? | Unclear risk | no complete report available |
| Were baseline outcome measurements similar? | Unclear risk | no complete report available |
| Was knowledge of the allocated interventions adequately prevented during the study? | Unclear risk | no complete report available |
| Was the study adequately protected against contamination | Unclear risk | no complete report available |
| Were incomplete outcome data adequately addressed? | Unclear risk | no complete report available |
| Was the study free from selective outcome reporting? | Unclear risk | no complete report available |
| Was the study free from other risks of bias? | Unclear risk | no complete report available |

### Holden 2018

| **Bias** | **Authors’ judgement** | **Support for judgement** |
| --- | --- | --- |
| Was the allocation sequence adequately generated? | High risk | non-randomised study |
| Was the allocation sequence adequately concealed? | High risk | non-randomised study |
| Were baseline characteristics similar? | Low risk | Controls matched regarding age and gender. No differences. No other characteristics reported |
| Were baseline outcome measurements similar? | Unclear risk | Matched cases/controls. There was a difference (p-value of .01) in weight before the workshops, with cases having a mean of 23.09 kg and controls 21.15 kg. no difference between CD4 count/adherence |
| Was knowledge of the allocated interventions adequately prevented during the study? | High risk | Not possible to blind participants due to the nature of the intervention. Clinical outcomes gathered from records. Data was anonymised before analysis |
| Was the study adequately protected against contamination | Unclear risk | Historical controls |
| Were incomplete outcome data adequately addressed? | Unclear risk | Not reported how many participants completed baseline and follow-up assessments, but clinical data was obtained from routine medical records |
| Was the study free from selective outcome reporting? | Unclear risk | Only clinical data for both groups. Intervention group participants were interviewed at follow-up |
| Was the study free from other risks of bias? | Low risk |  |

### Hosek 2018

| **Bias** | **Authors’ judgement** | **Support for judgement** |
| --- | --- | --- |
| Was the allocation sequence adequately generated? | Unclear risk | Not reported how random sequence was generated |
| Was the allocation sequence adequately concealed? | Unclear risk | Not reported how allocation was concealed |
| Were baseline characteristics similar? | High risk | The randomization process resulted in equivalent groupson almost all baseline demographic characteristics, with theexception of four measures: days since tested HIV positive;days since first sought treatment for HIV; ever receiving adiagnosis of AIDS; and taking HIV medications (Table 3).The ACCEPT group had a shorter average time sincetested positive (191 days) than the HEALTH group average(289 days) (p = 0.025); a shorter average time since firstsought treatment for HIV (171 days vs 269 days, p = 0.027);and a lower proportion having received an AIDS diagnosis(3.5% vs. 19.6%, p = 0.009)." |
| Were baseline outcome measurements similar? | Low risk | No differences in outcomes reported at baseline |
| Was knowledge of the allocated interventions adequately prevented during the study? | High risk | Blinding of participants and personnel not possible due to the nature of the intervention. Outcome measures mostly self-reported |
| Was the study adequately protected against contamination | Unclear risk | Not described. Group sessions ran concurrently, but randomisation occurred at individual (not clinic) level, not sure that there was no contamination |
| Were incomplete outcome data adequately addressed? | Low risk | Loss to follow-up in intervention: 10/57 (17.5%); control 8/46 (17.4%)Although close to 20% loss to follow-up, this is similar between groups |
| Was the study free from selective outcome reporting? | Low risk | All outcomes pre-specified in methods section reported on |
| Was the study free from other risks of bias? | Low risk |  |

### Jeffries 2016

| **Bias** | **Authors’ judgement** | **Support for judgement** |
| --- | --- | --- |
| Was the allocation sequence adequately generated? | Unclear risk | Full report not available |
| Was the allocation sequence adequately concealed? | Unclear risk | Full report not available |
| Were baseline characteristics similar? | Unclear risk | Full report not available |
| Were baseline outcome measurements similar? | Unclear risk | Full report not available |
| Was knowledge of the allocated interventions adequately prevented during the study? | Unclear risk | Full report not available |
| Was the study adequately protected against contamination | Unclear risk | Full report not available |
| Were incomplete outcome data adequately addressed? | Unclear risk | Full report not available |
| Was the study free from selective outcome reporting? | Unclear risk | Full report not available |
| Was the study free from other risks of bias? | Unclear risk | Full report not available |

### Letourneau 2013

| **Bias** | **Authors’ judgement** | **Support for judgement** |
| --- | --- | --- |
| Was the allocation sequence adequately generated? | High risk | Method of generating random sequence not reported. 4/20 (20%) participants were assigned to the intervention group (not randomised). |
| Was the allocation sequence adequately concealed? | High risk | Not reported. 4/20 (20%) participants were assigned to the intervention group (not randomised) |
| Were baseline characteristics similar? | Unclear risk | Not reported per group. |
| Were baseline outcome measurements similar? | Low risk | Baseline outcome measurements for Viral load, CD4 count and adherence were similar between groups |
| Was knowledge of the allocated interventions adequately prevented during the study? | High risk | Blinding of patients and personnel: High - not blinded, families were incentivized. Blinding of outcome assessors: unclear |
| Was the study adequately protected against contamination | Unclear risk | Not stated if participants were excluded if they participated in other interventions. |
| Were incomplete outcome data adequately addressed? | Low risk | No loss to follow-up |
| Was the study free from selective outcome reporting? | Low risk | All outcomes pre-specified in methods section were reported on |
| Was the study free from other risks of bias? | Low risk | No other risk of bias identified |

### Mimiaga 2018

| **Bias** | **Authors’ judgement** | **Support for judgement** |
| --- | --- | --- |
| Was the allocation sequence adequately generated? | Low risk | Randomisation sequence generated by a computer |
| Was the allocation sequence adequately concealed? | Unclear risk | not reported |
| Were baseline characteristics similar? | Unclear risk | baseline characteristics not reported according to group allocation. Authors report: "Both the intervention and control groups did not significantly differ with respect to baseline sociodemographics and adherence scores" |
| Were baseline outcome measurements similar? | Unclear risk | No data presented. IN the text authors report: "Both the intervention and control groups did not significantly differ with respect to baseline sociodemographics and adherence scores." |
| Was knowledge of the allocated interventions adequately prevented during the study? | High risk | Blinding not possible due to the nature of the intervention. Outcomes were self-reported |
| Was the study adequately protected against contamination | Unclear risk | Not described how contamination was avoided |
| Were incomplete outcome data adequately addressed? | Unclear risk | Loss-to-follow-up not reported, no flow diagram present |
| Was the study free from selective outcome reporting? | Unclear risk | Outcome data not clearly reported |
| Was the study free from other risks of bias? | Unclear risk | Very short report of pilot study |

### Naar King 2006

| **Bias** | **Authors’ judgement** | **Support for judgement** |
| --- | --- | --- |
| Was the allocation sequence adequately generated? | Unclear risk | Not reported how sequence was generated: 'Statistician' randomised participants |
| Was the allocation sequence adequately concealed? | Unclear risk | Not described how allocation was concealed |
| Were baseline characteristics similar? | Unclear risk | Baseline characteristics not reported per group. |
| Were baseline outcome measurements similar? | Low risk | There were no differences between the treatment and control group in baseline condom use, alcohol use, marijuana use, or log viral load."Although data is not shown |
| Was knowledge of the allocated interventions adequately prevented during the study? | High risk | Not able to blind participants and personnel. Outcomes on health risks self-reported. not reported whether outcome assessors for viral load were blinded |
| Was the study adequately protected against contamination | Unclear risk | Not described how contamination was avoided |
| Were incomplete outcome data adequately addressed? | High risk | Comment: more than 20% loss to follow-up; 19% in intervention and 21% in control group |
| Was the study free from selective outcome reporting? | Low risk | Comment: more than 20% loss to follow-up; 19% in intervention and 21% in control group |
| Was the study free from other risks of bias? | Low risk |  |

### Naar King 2009

| **Bias** | **Authors’ judgement** | **Support for judgement** |
| --- | --- | --- |
| Was the allocation sequence adequately generated? | Unclear risk | Although randmonisation process described (used automated clinical trial management tool based on telephone interactive voice-response technology), it is not described how the randomisation sequence was generated - assumed computer-generated? |
| Was the allocation sequence adequately concealed? | Low risk | An automated clinical trial management tool based ontelephone interactive voice-response technology was used torandomize subjects to their treatment arm. Using state-of-the-arttechnology, this tool allows users to send and receive randomization information from any telephone. |
| Were baseline characteristics similar? | Unclear risk | there were significantly fewer biological males in the intervention group than in the control group (44.7% vs 60.9%, p=0.03) |
| Were baseline outcome measurements similar? | Low risk | No difference in viral load at baseline |
| Was knowledge of the allocated interventions adequately prevented during the study? | High risk | Not able to blind participants and personnel due to the nature of the intervention. Outcome assessor of viral load blinded |
| Was the study adequately protected against contamination | Unclear risk | Not reported how contamination was prevented |
| Were incomplete outcome data adequately addressed? | High risk | Total randomised n=205Total at 6 months follow-up: n=157 (23% LTFU). Total at 9 months follow-up: n=146 (29% LTFU). Follow-up rate in intervention group: 86% (6 months); 82% (9 months)Follow-up rate in control group: 81% (6 months); 73% (9 months) |
| Was the study free from selective outcome reporting? | Low risk | All outcomes pre-specified in methods section reported on |
| Was the study free from other risks of bias? | Low risk | No other risks of bias detected |

### Rongkavilit 2013

| **Bias** | **Authors’ judgement** | **Support for judgement** |
| --- | --- | --- |
| Was the allocation sequence adequately generated? | Unclear risk | Not described how allocation sequence was generated |
| Was the allocation sequence adequately concealed? | Unclear risk | Not reported |
| Were baseline characteristics similar? | Low risk | Baseline characteristics appear similar, no statistical significant differences. |
| Were baseline outcome measurements similar? | Unclear risk | Baseline outcome data not compared for differences. Looking at Table 3 there seem to be some difference. Low power to detect differences. |
| Was knowledge of the allocated interventions adequately prevented during the study? | High risk | Not possible to blind personnel and participants and therefore high risk of performance bias. Self-reported outcome, therefore high risk of detection bias |
| Was the study adequately protected against contamination | Unclear risk | Not described how contamination was avoided apart from reporting that facilitators in the control group was not trained in MI. |
| Were incomplete outcome data adequately addressed? | High risk | Loss-to-follow-up at 6 months: 10.9% (6/55) intervention group vs. 24% (13/55) in control group |
| Was the study free from selective outcome reporting? | Low risk | All outcomes pre-specified in methods section reported on |
| Was the study free from other risks of bias? | Low risk |  |

### Webb 2018

| **Bias** | **Authors’ judgement** | **Support for judgement** |
| --- | --- | --- |
| Was the allocation sequence adequately generated? | Low risk | Computer-generated sequence |
| Was the allocation sequence adequately concealed? | Unclear risk | Not reported |
| Were baseline characteristics similar? | Unclear risk | Baseline characteristics similar although only gender and age reported. |
| Were baseline outcome measurements similar? | Low risk | Baseline outcomes similar |
| Was knowledge of the allocated interventions adequately prevented during the study? | High risk | Not able to blind personnel and participants due to the nature of the intervention. Some self-reported outcomes. Clinical outcomes from medical records - not reported whether outcome assessors were blinded |
| Was the study adequately protected against contamination | Unclear risk | Control sessions were matched to MBSR sessions wrt number and length of classes, location, didactic and experiential structure. But no cluster randomisation, therefore not clear whether some contamination might have occurred. |
| Were incomplete outcome data adequately addressed? | High risk | Total randomised: n=96, total analysed: n=72. Overall, more than 20% attrition, although equal between groups. baseline n = 72, post-program n = 52 (72%), three-month follow-up n = 40 (55%). |
| Was the study free from selective outcome reporting? | Low risk | All outcomes pre-specified in methods section reported on |
| Was the study free from other risks of bias? | Low risk |  |

### Whiteley 2018

| **Bias** | **Authors’ judgement** | **Support for judgement** |
| --- | --- | --- |
| Was the allocation sequence adequately generated? | Low risk | Randomisation sequence generated with computer software (REDCap) |
| Was the allocation sequence adequately concealed? | Unclear risk | Not reported how allocation was concealed |
| Were baseline characteristics similar? | Low risk | Baseline characteristics similar. see table 1 |
| Were baseline outcome measurements similar? | Unclear risk | Although authors report that there was no difference, some of the pre-test values in Table 2 are different between groups. No statistical test to show differences. |
| Was knowledge of the allocated interventions adequately prevented during the study? | High risk | Blinding not possible due to nature of the intervention. But knowledge of the intervention could have influenced performance. Self-reported outcomes. |
| Was the study adequately protected against contamination | Unclear risk | Participants recruited from HIV clinics in the greater Jackson Mississippi area. But not reported whether participants might have had contact with each other or how contamination was avoided |
| Were incomplete outcome data adequately addressed? | High risk | Flow-diagram not complete - does not show flow of participants through both groups. Follow-up data for viral load missing in 10 participants, 23% in intervention vs. 10% in control group |
| Was the study free from selective outcome reporting? | High risk | Follow-up data on psychological distress and sexual activity not reported |
| Was the study free from other risks of bias? | Low risk |  |
