# Supplementary material for: Self-management interventions for adolescents living with HIV: a systematic review
Source: BMC Infect Dis. 2021 May 7;21:431. doi: 10.1186/s12879-021-06072-0 (PMC8105944; doi:10.1186/s12879-021-06072-0)
Supplement: Supplementary file 6 — Additional file 6. Forest plots. [file 12879_2021_6072_MOESM6_ESM.pptx]

## Slide 1
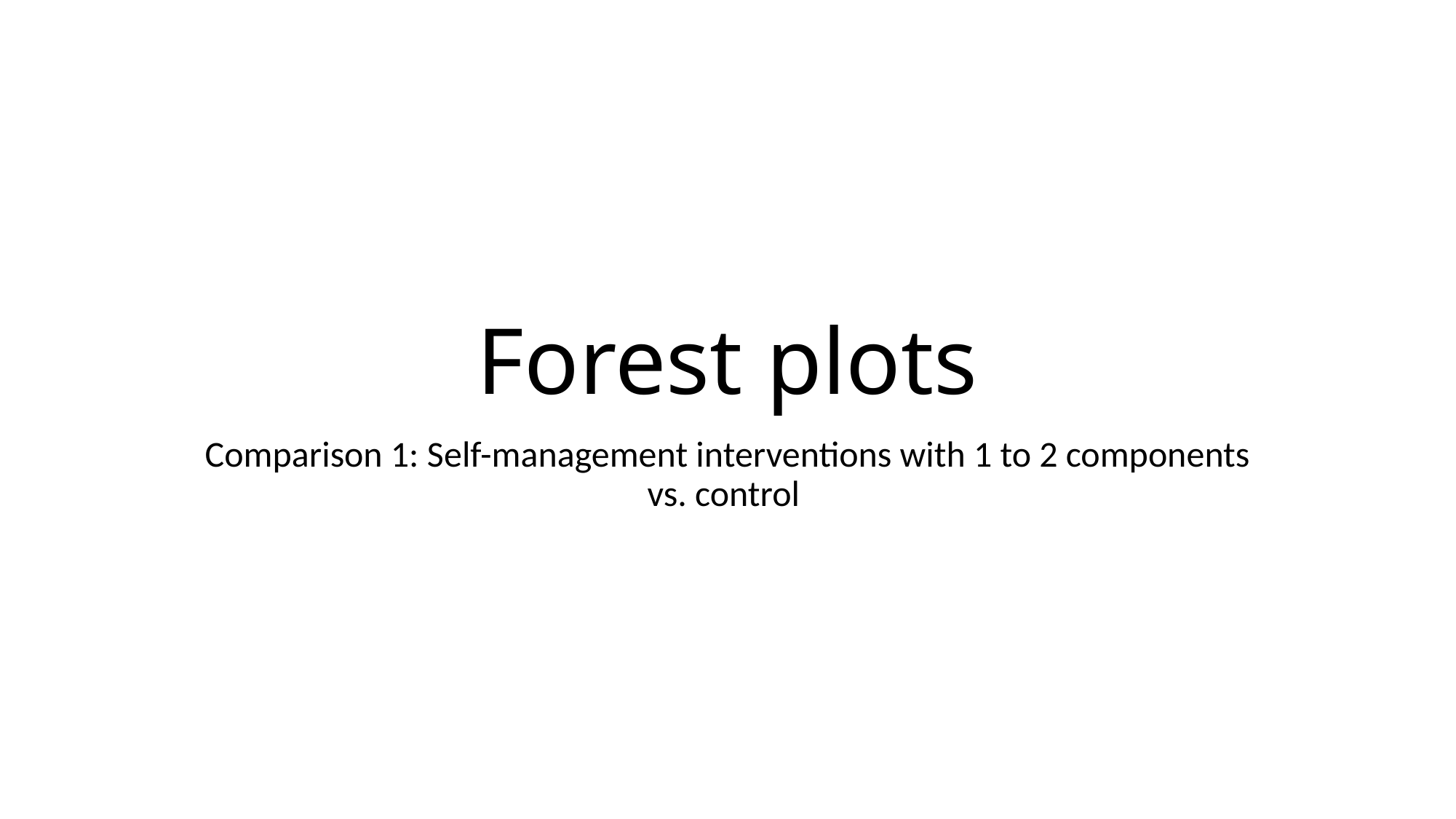

# Forest plots
Comparison 1: Self-management interventions with 1 to 2 components vs. control

## Slide 2
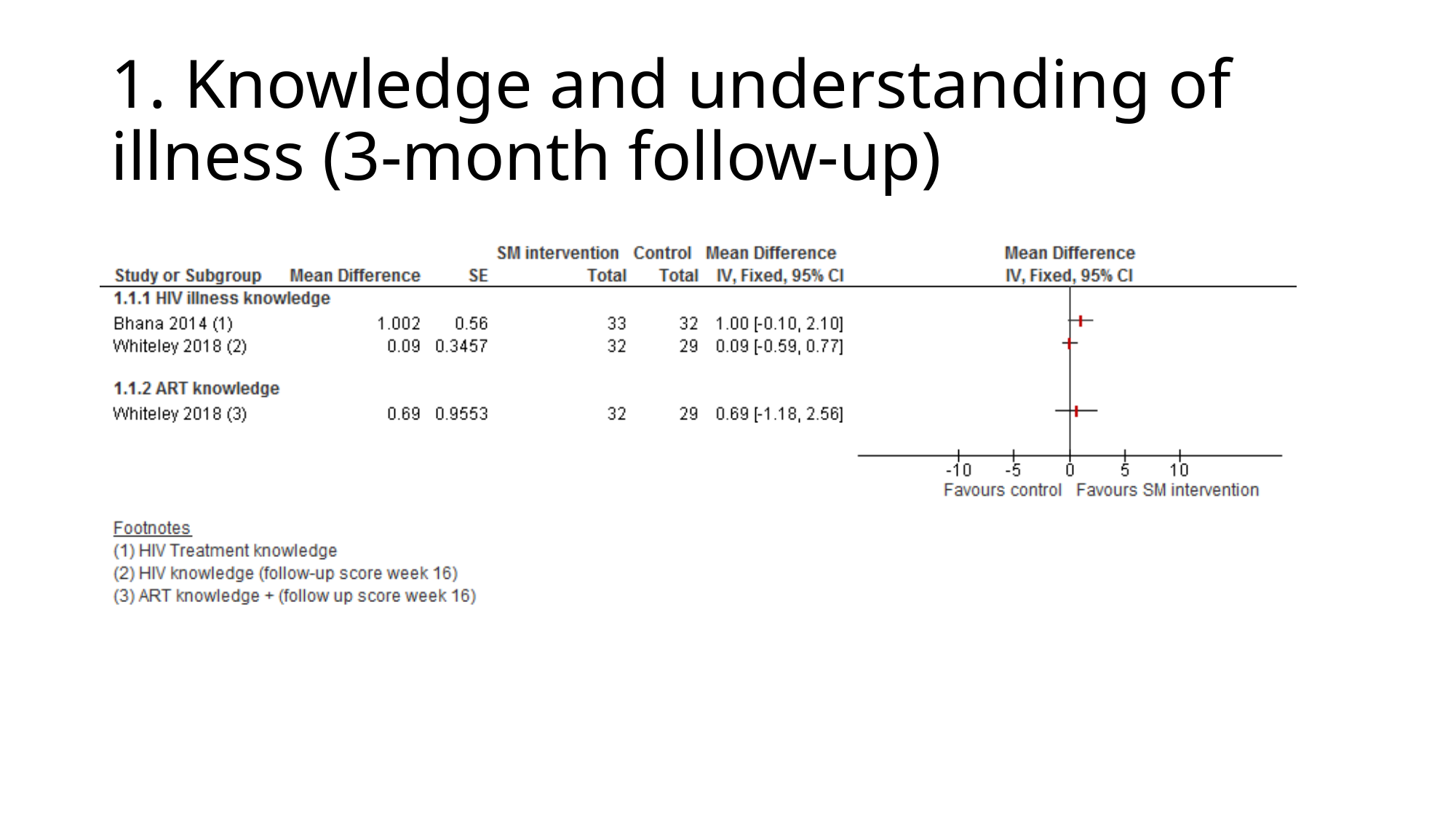

# 1. Knowledge and understanding of illness (3-month follow-up)

## Slide 3
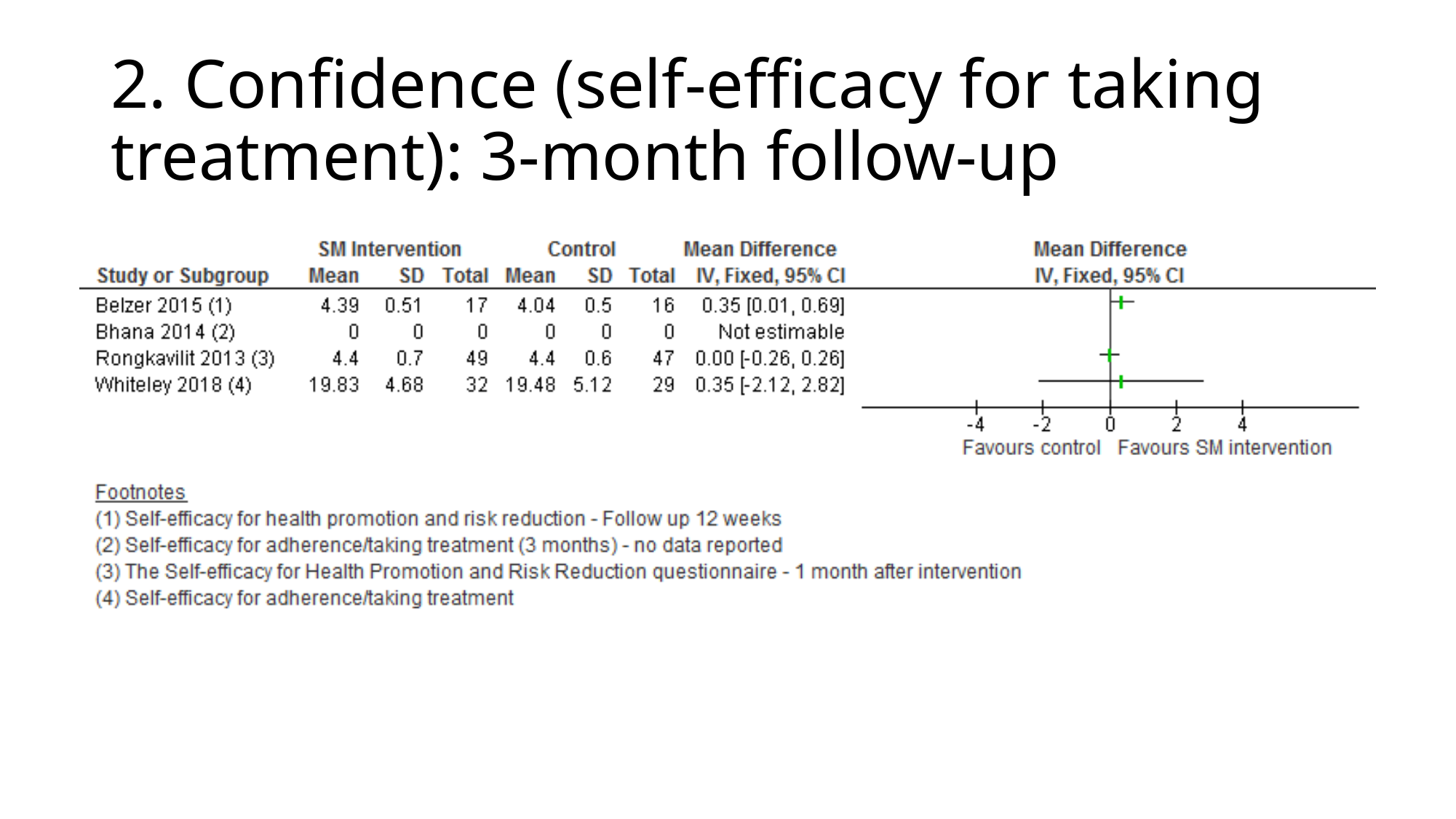

# 2. Confidence (self-efficacy for taking treatment): 3-month follow-up

## Slide 4
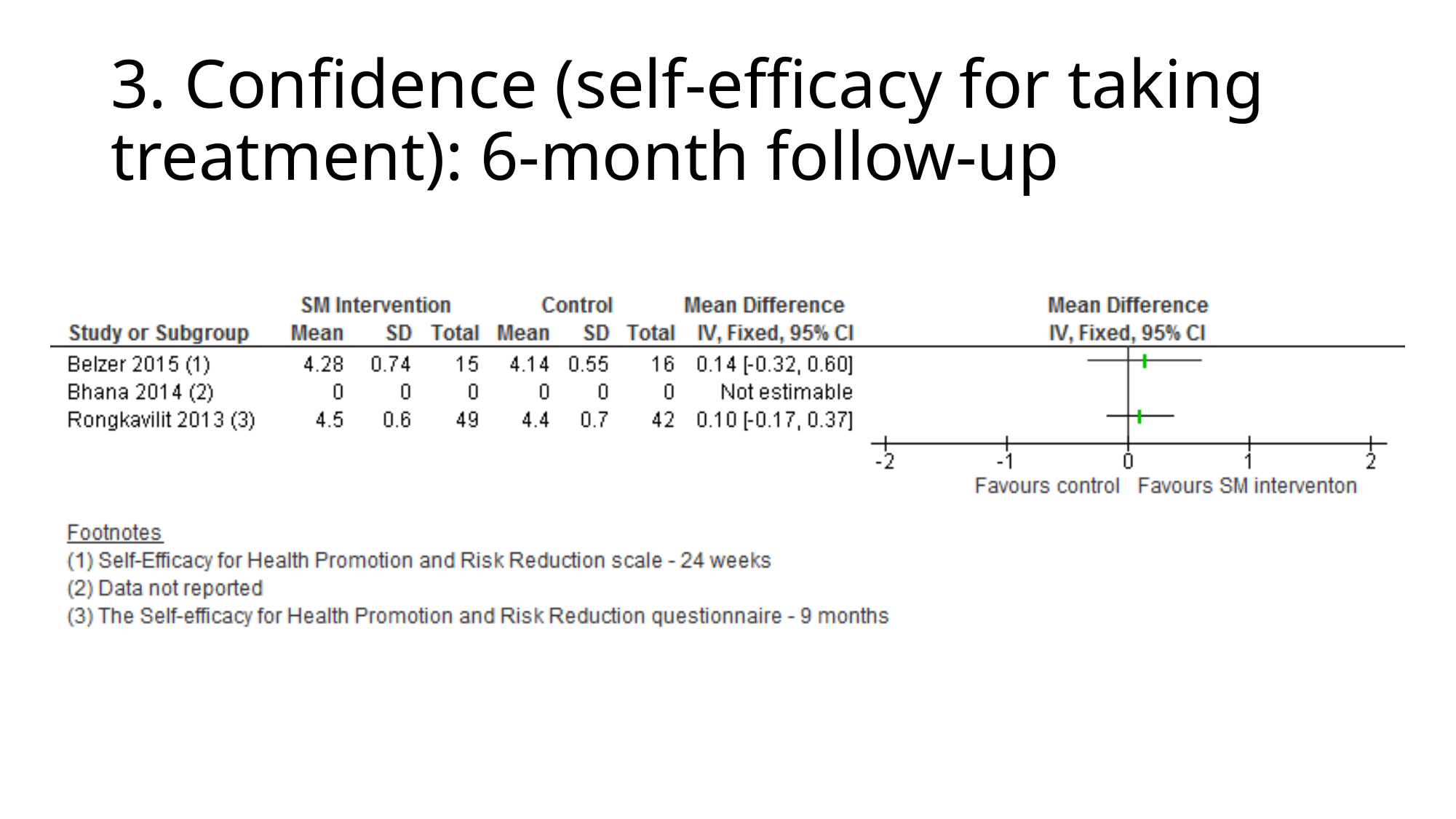

# 3. Confidence (self-efficacy for taking treatment): 6-month follow-up

## Slide 5
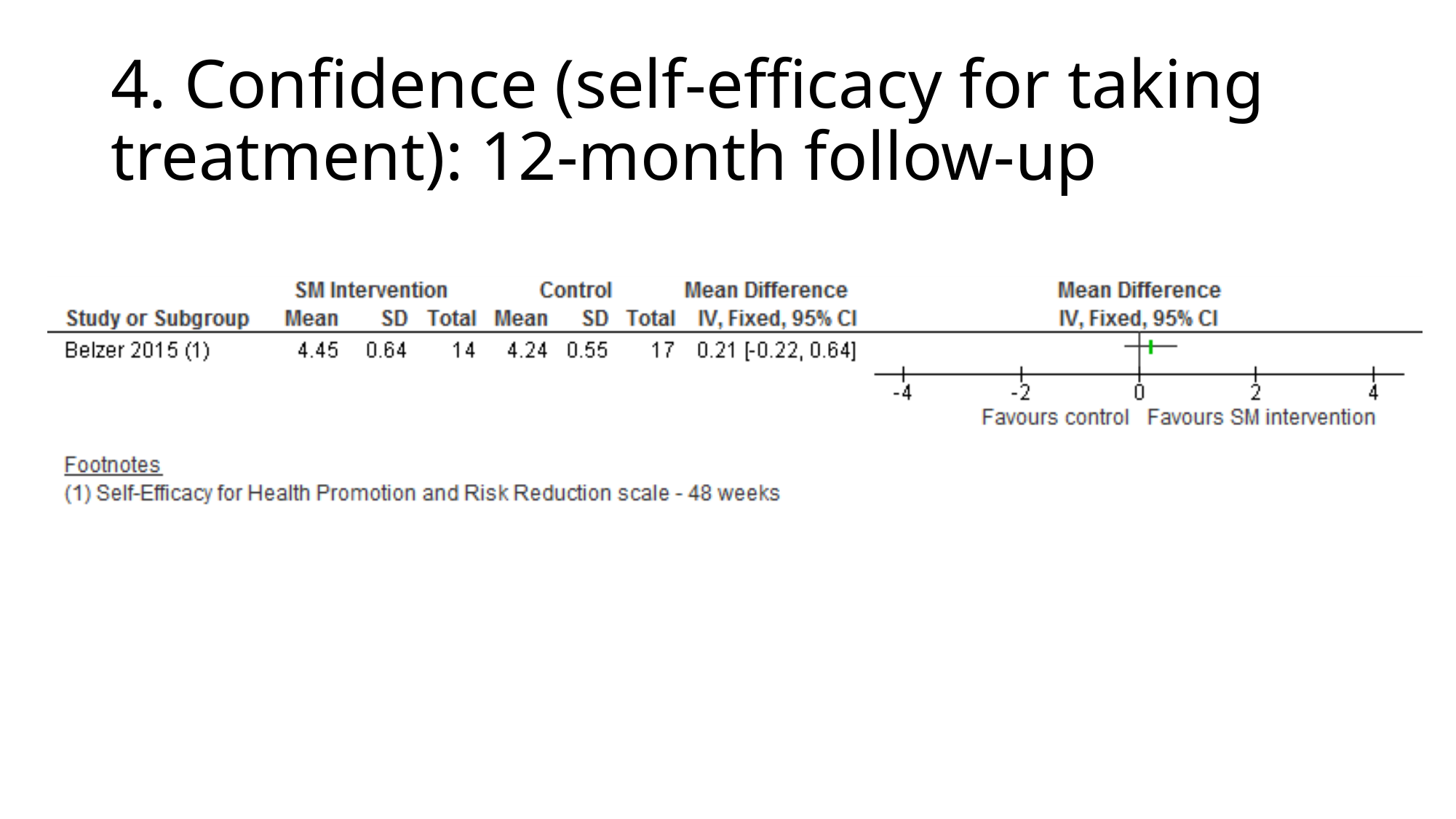

# 4. Confidence (self-efficacy for taking treatment): 12-month follow-up

## Slide 6
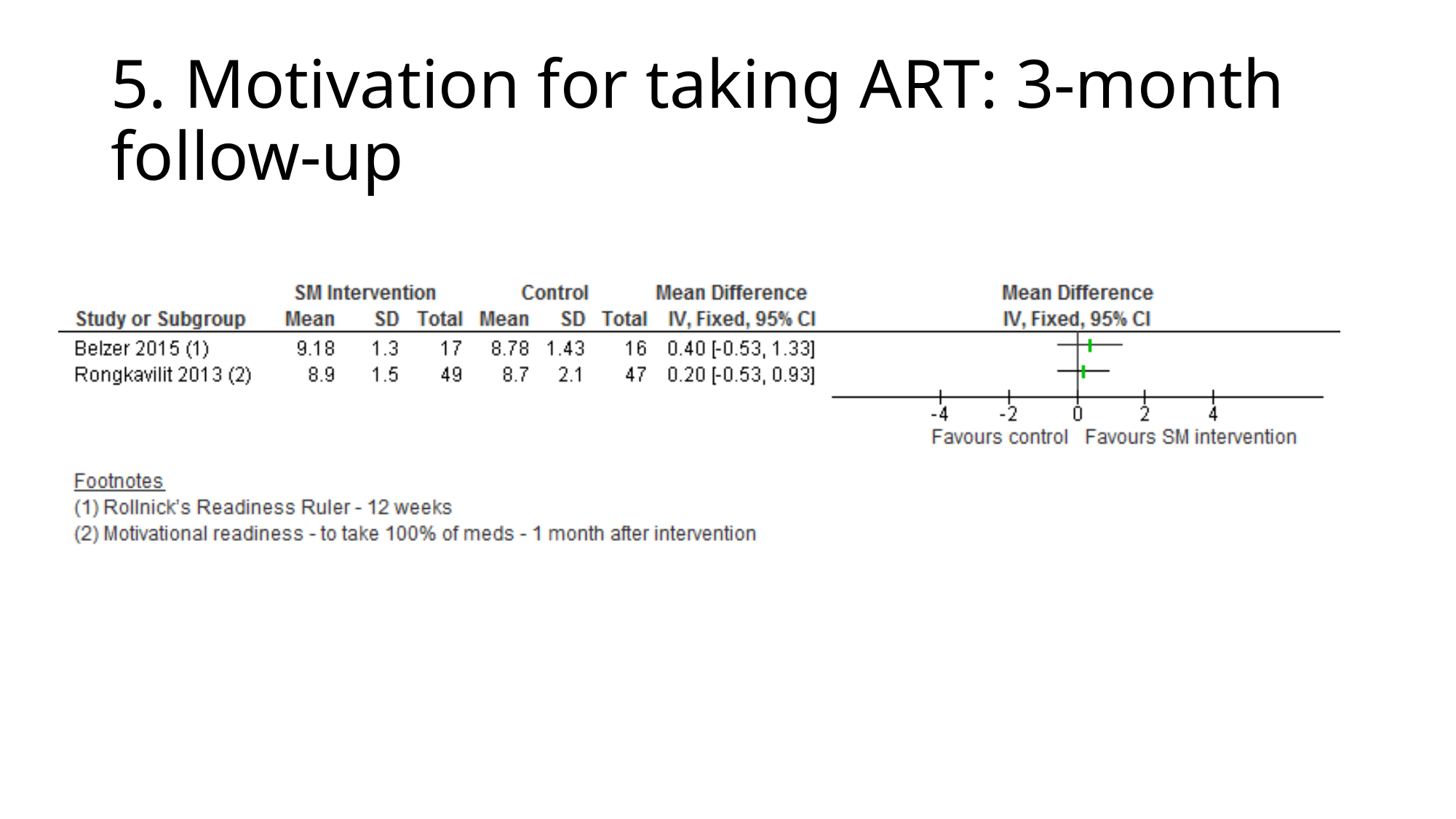

# 5. Motivation for taking ART: 3-month follow-up

## Slide 7
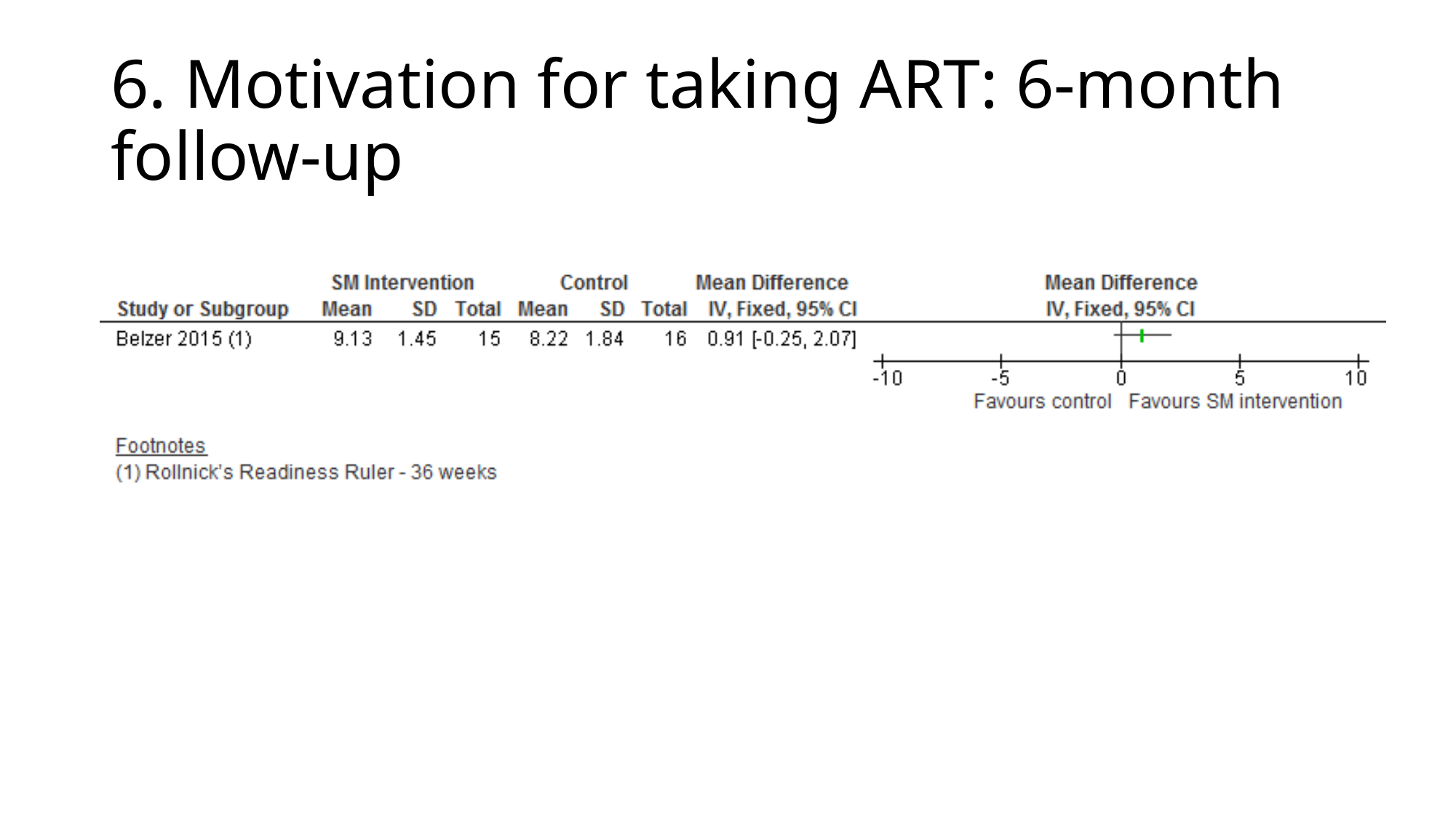

# 6. Motivation for taking ART: 6-month follow-up

## Slide 8
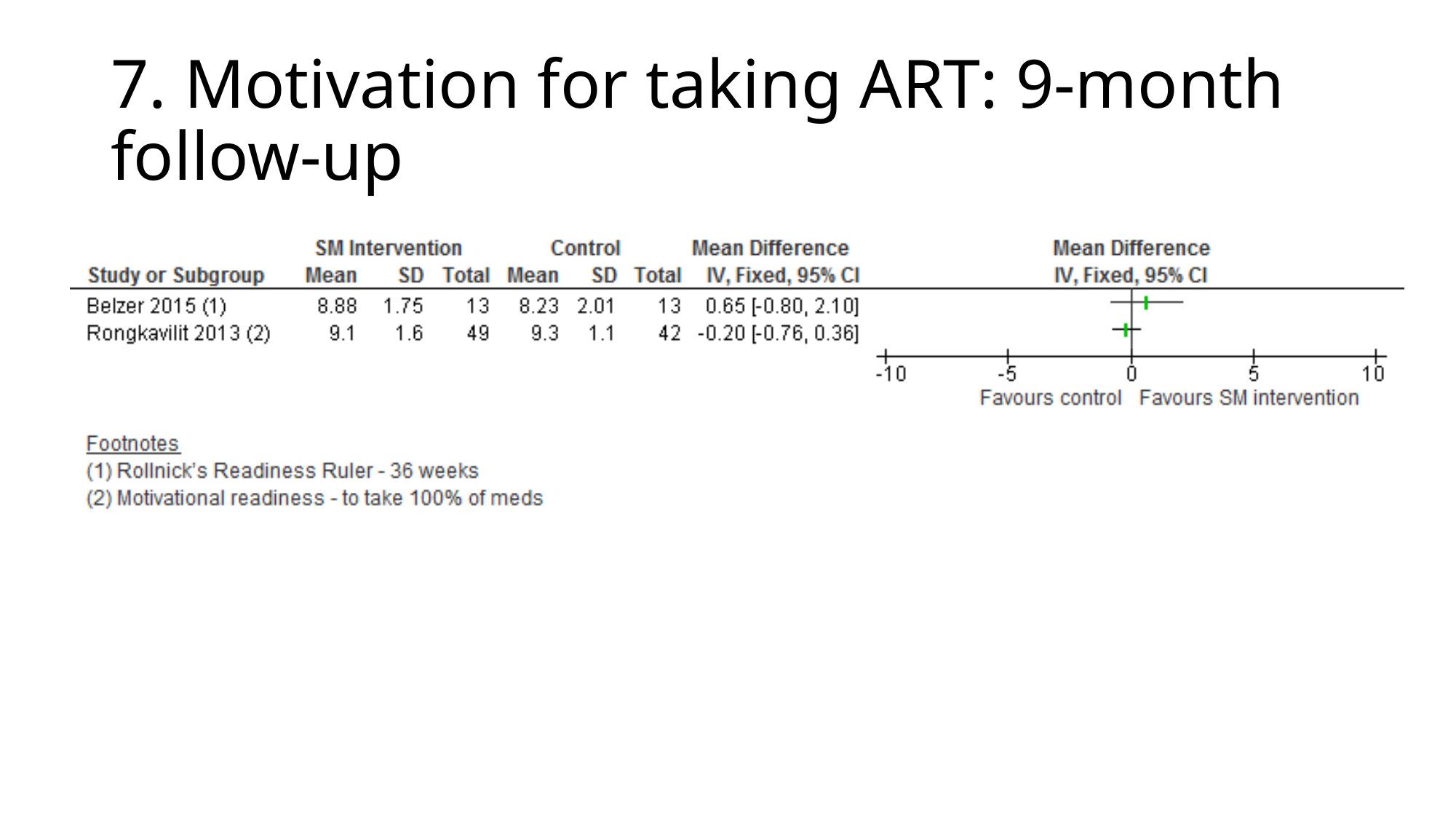

# 7. Motivation for taking ART: 9-month follow-up

## Slide 9
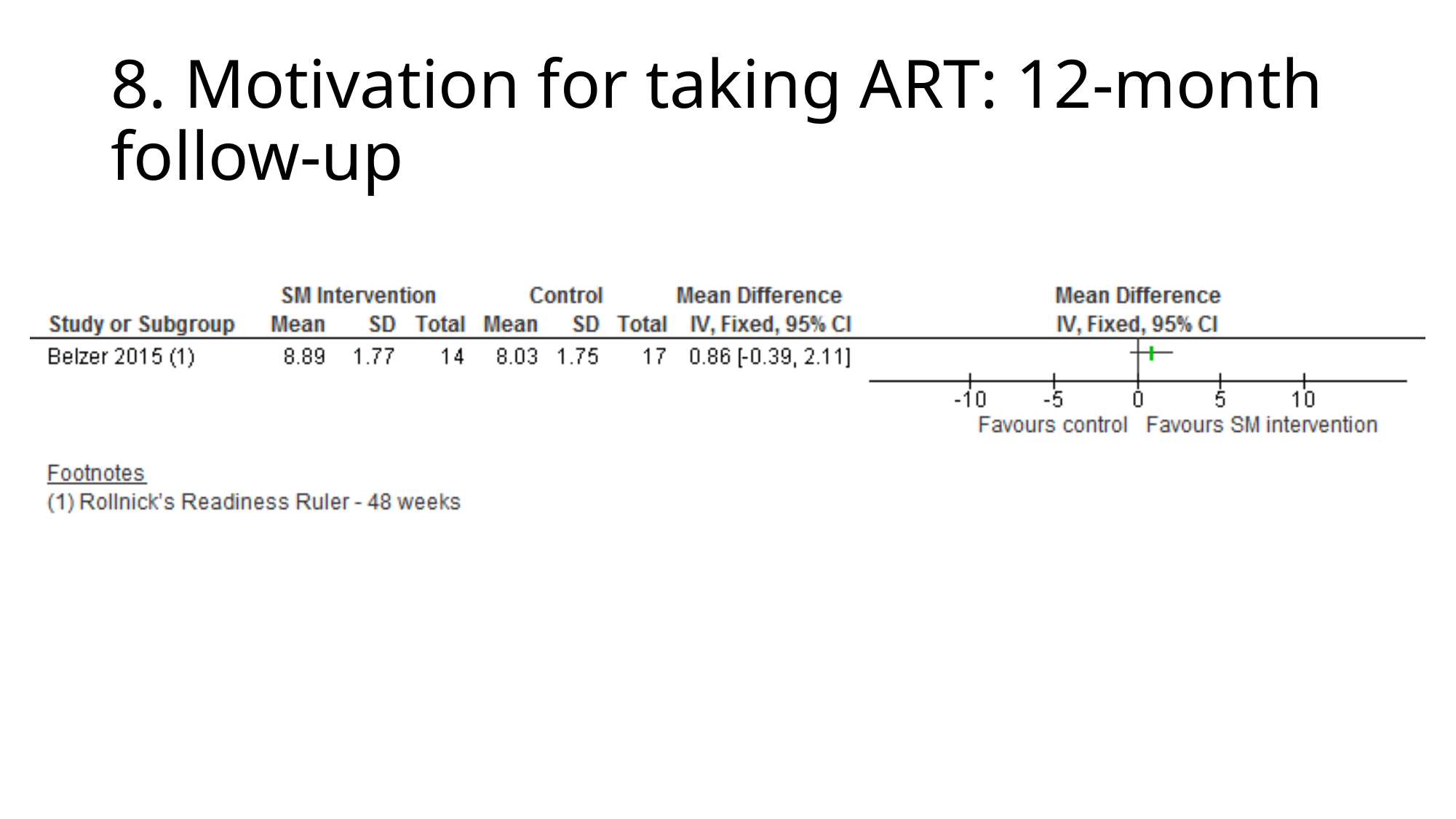

# 8. Motivation for taking ART: 12-month follow-up

## Slide 10
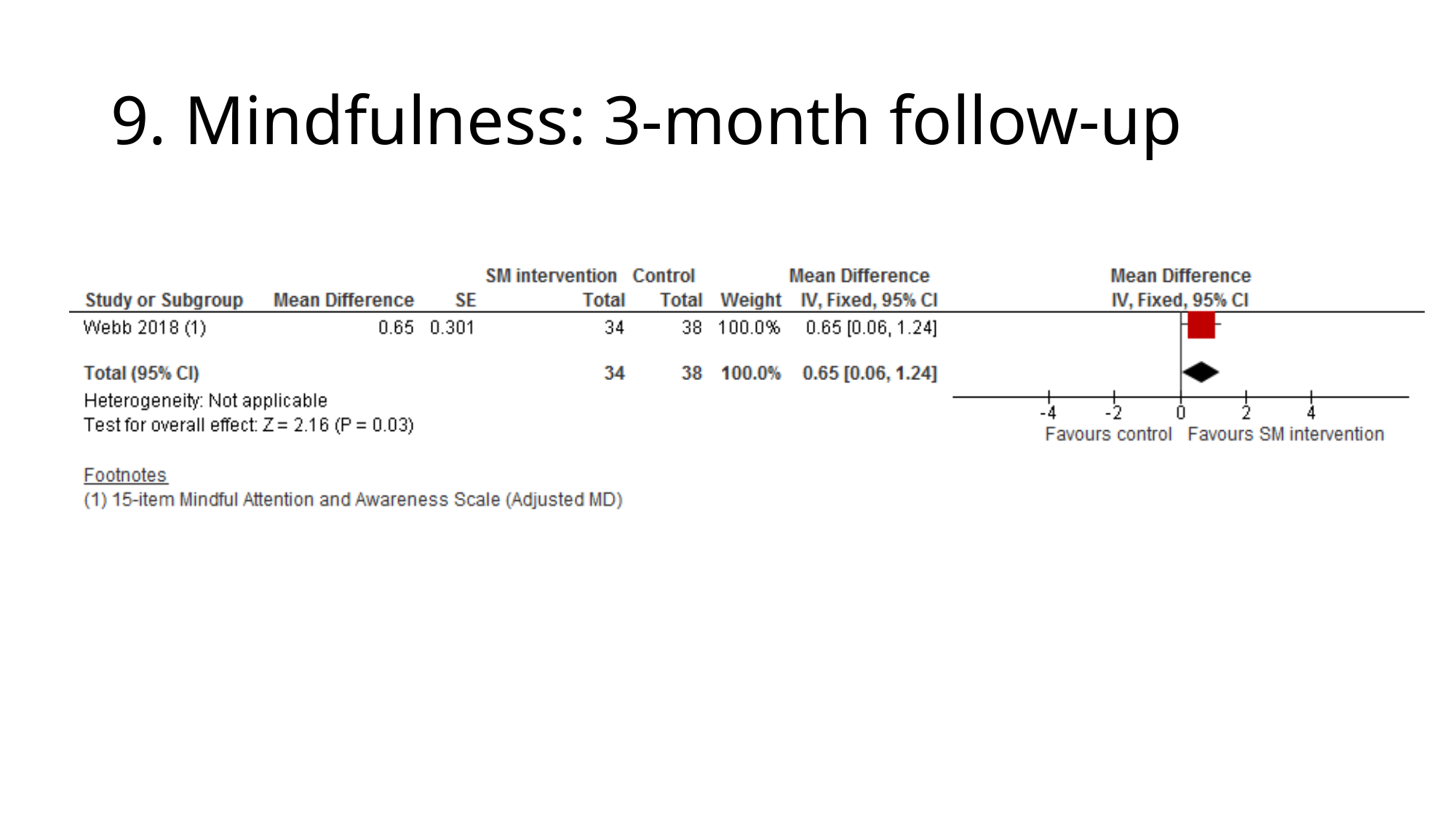

# 9. Mindfulness: 3-month follow-up

## Slide 11
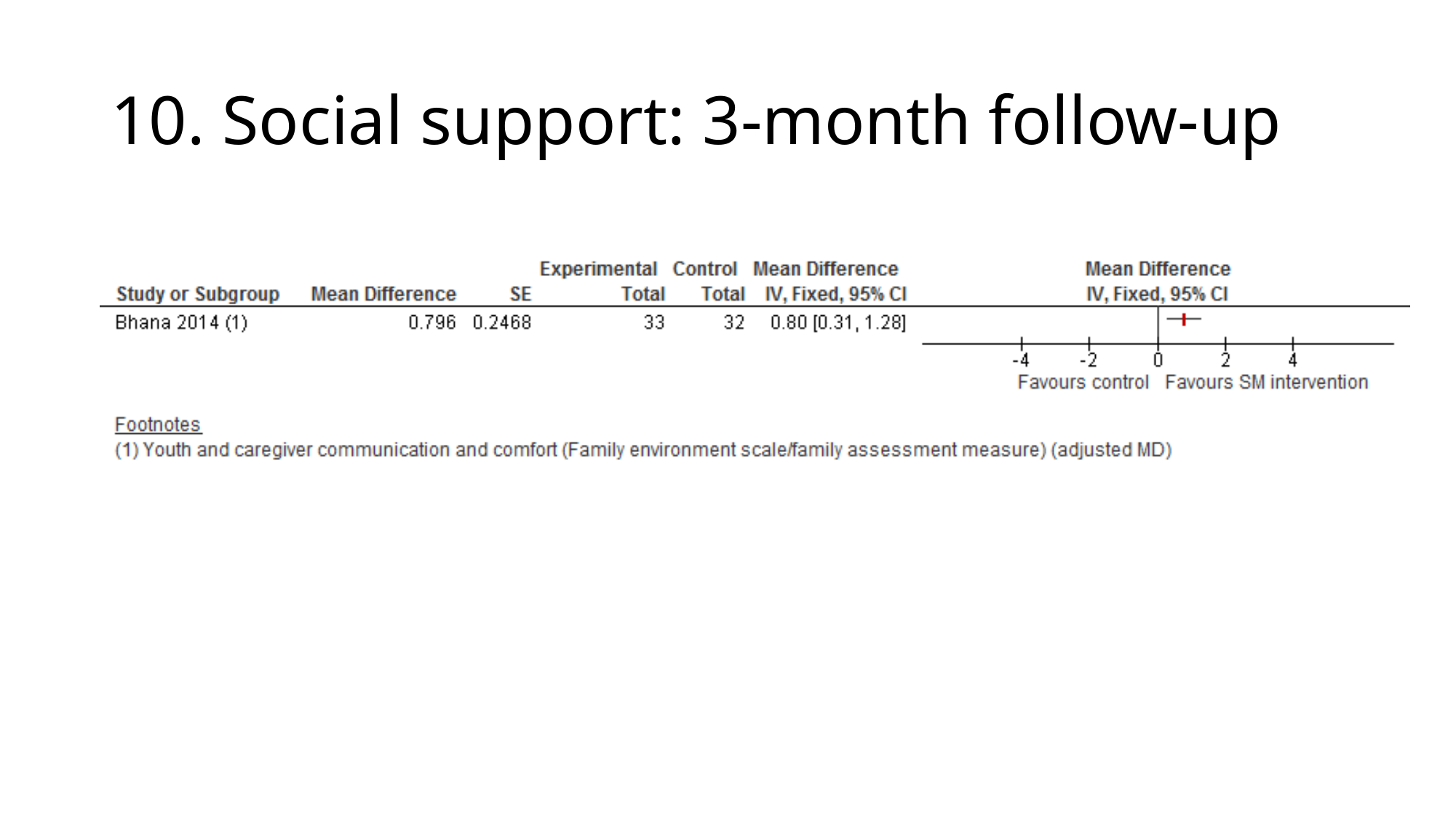

# 10. Social support: 3-month follow-up

## Slide 12
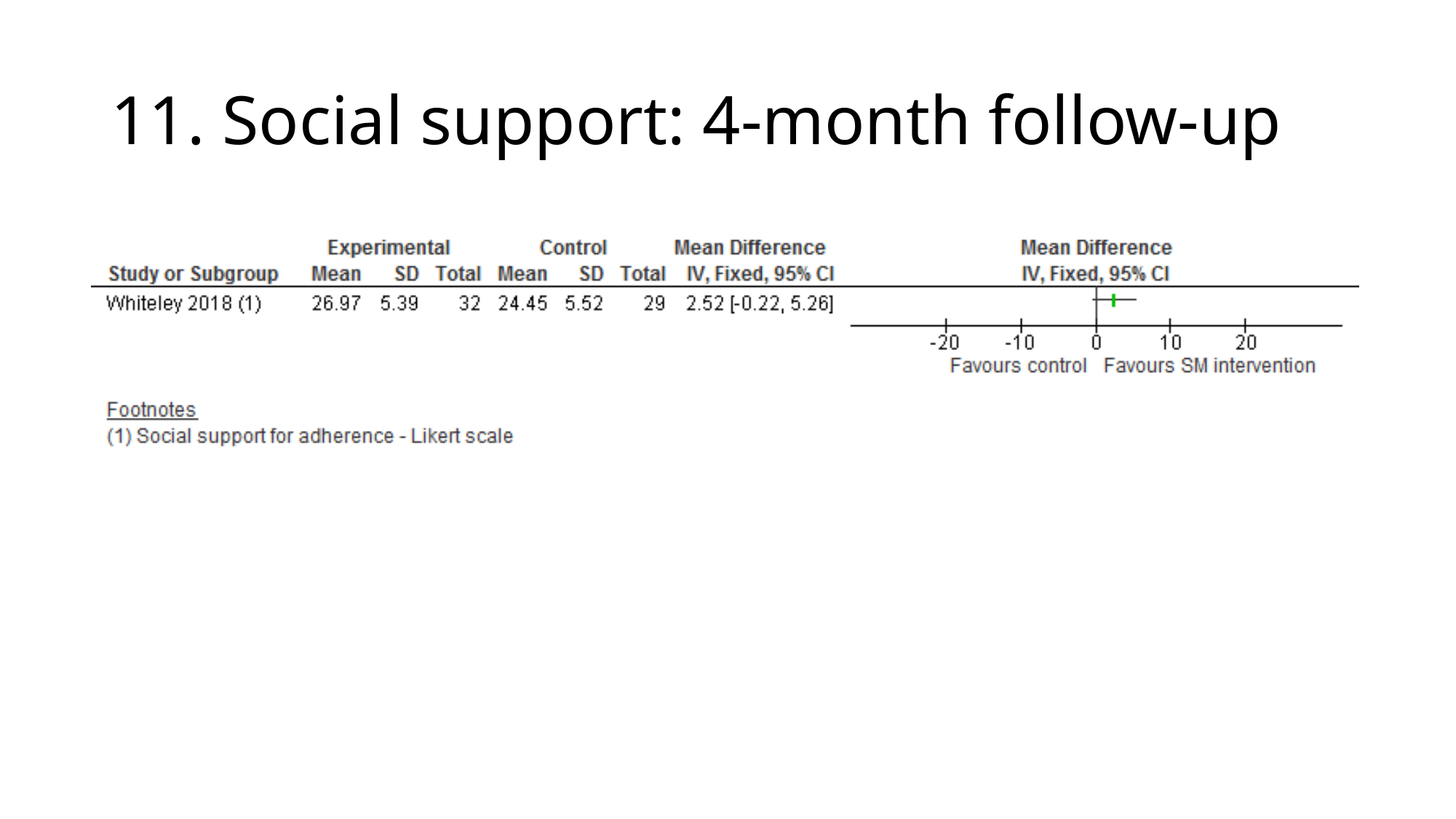

# 11. Social support: 4-month follow-up

## Slide 13
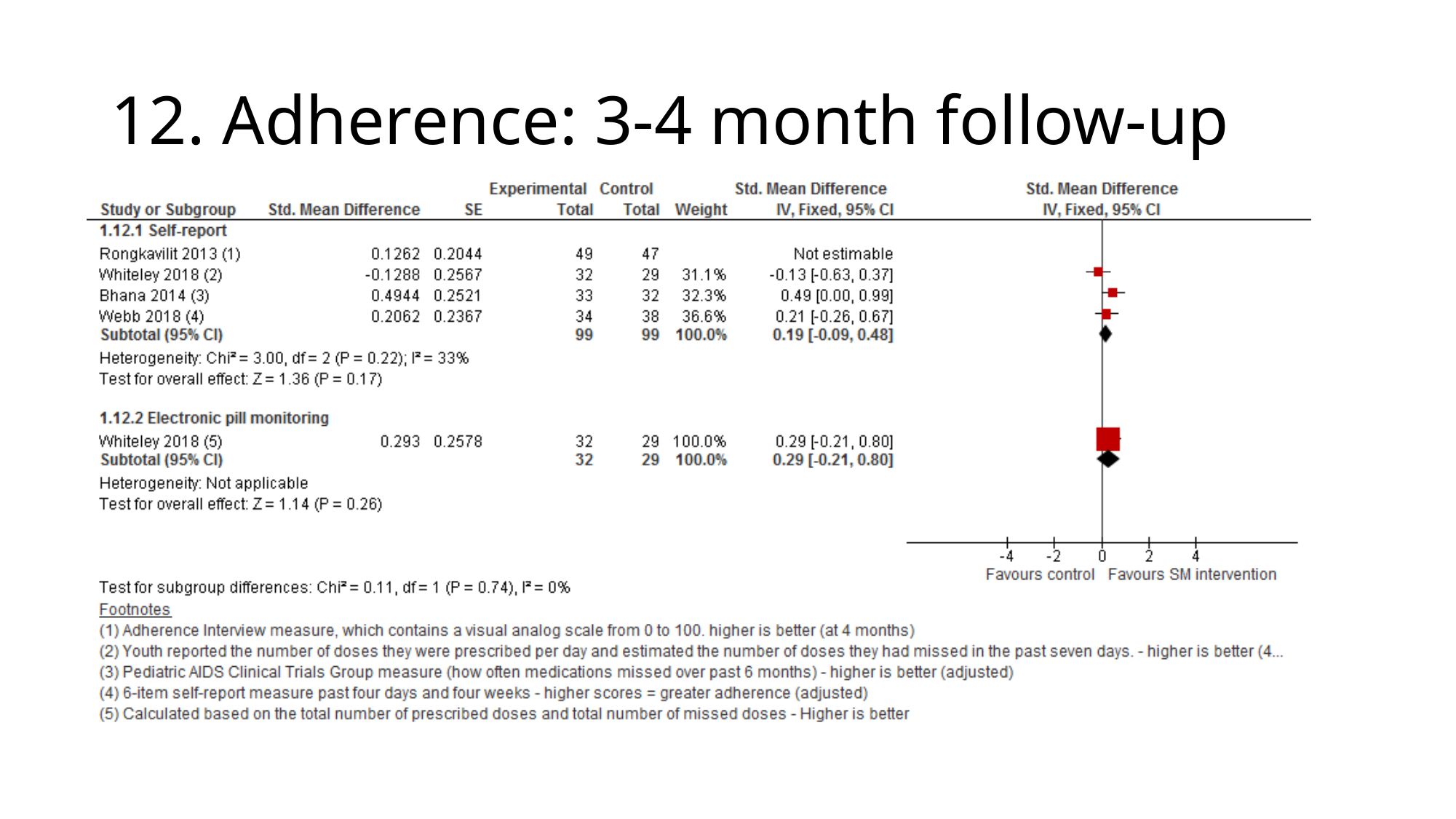

# 12. Adherence: 3-4 month follow-up

## Slide 14
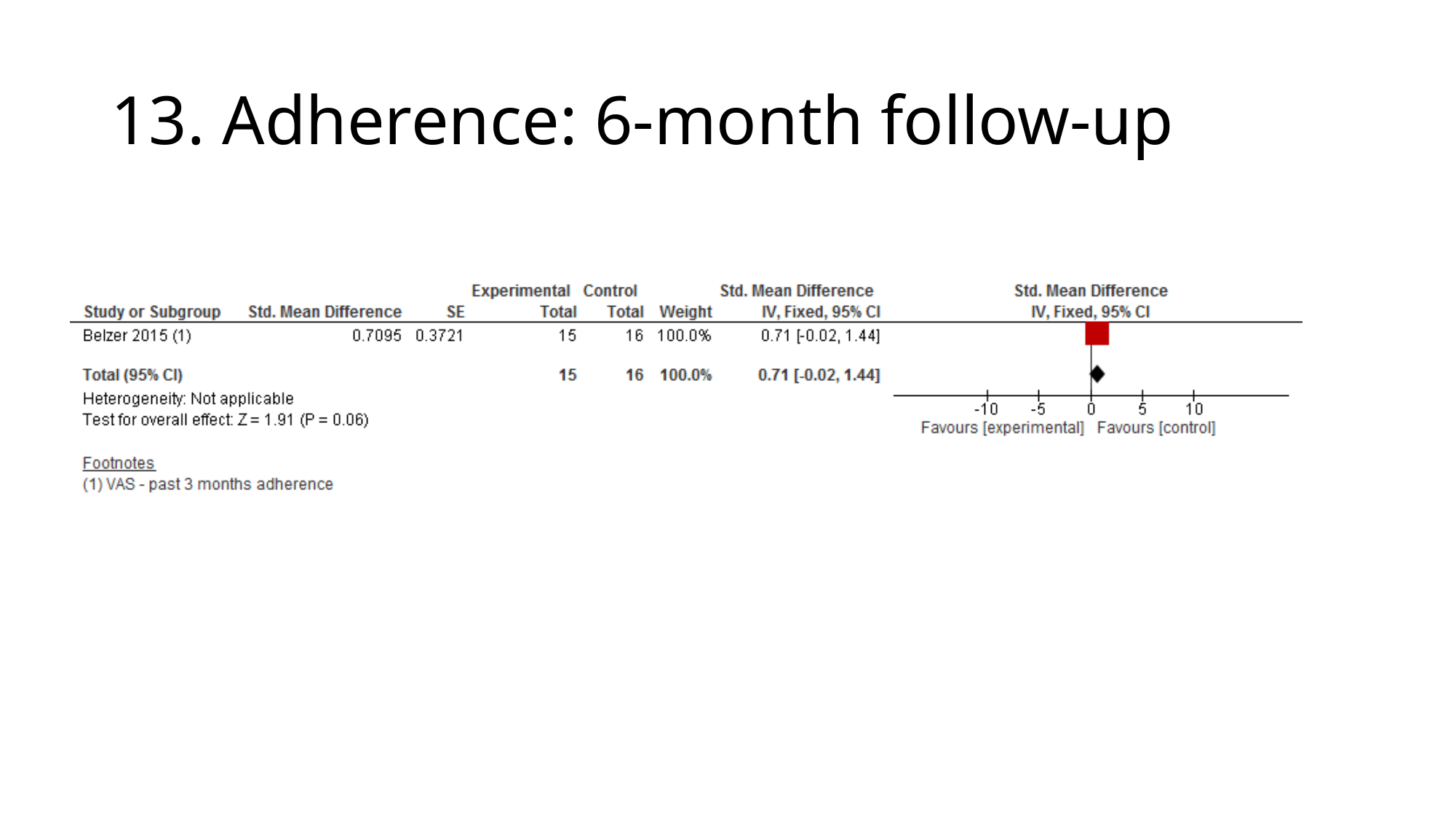

# 13. Adherence: 6-month follow-up

## Slide 15
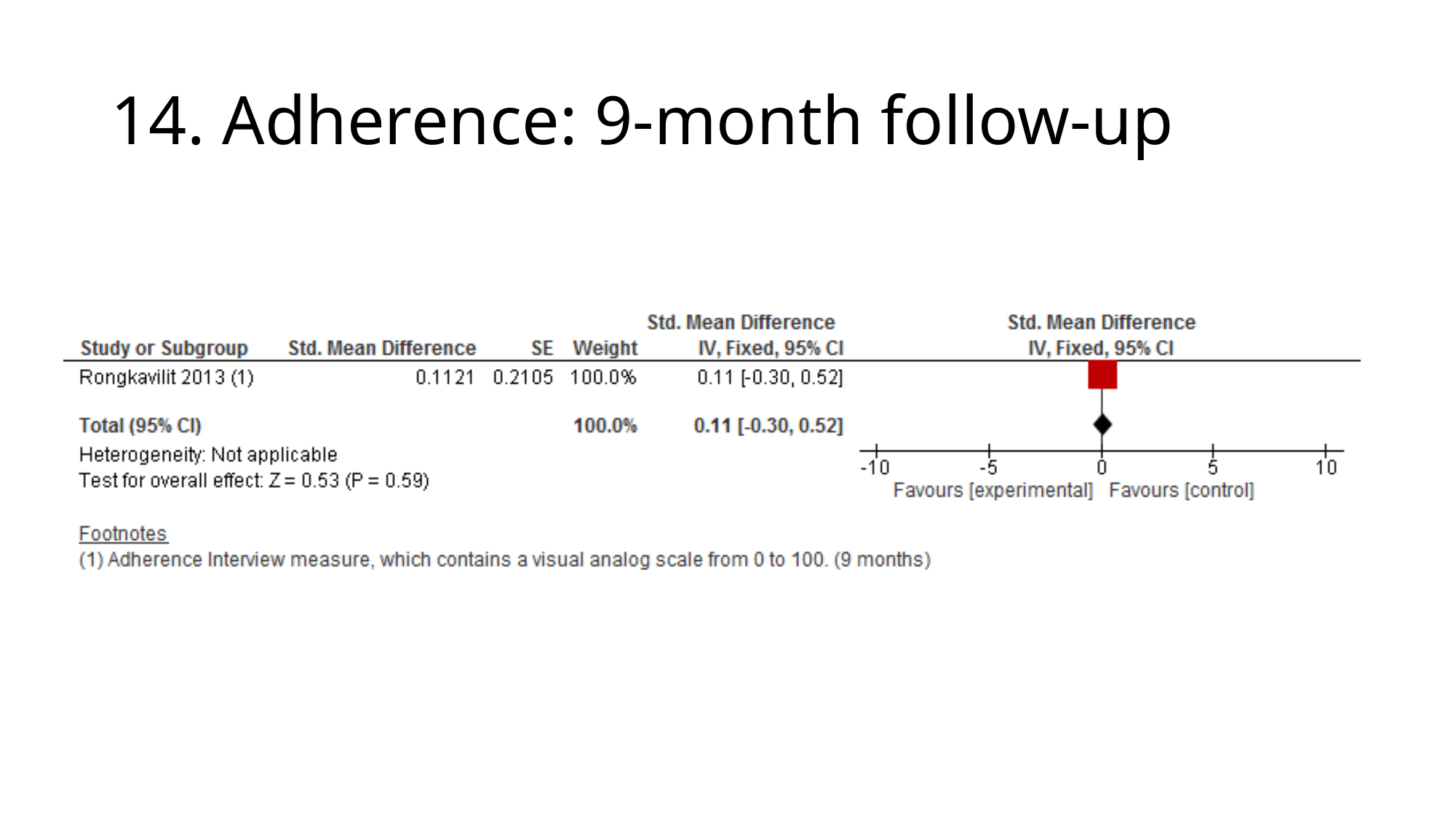

# 14. Adherence: 9-month follow-up

## Slide 16
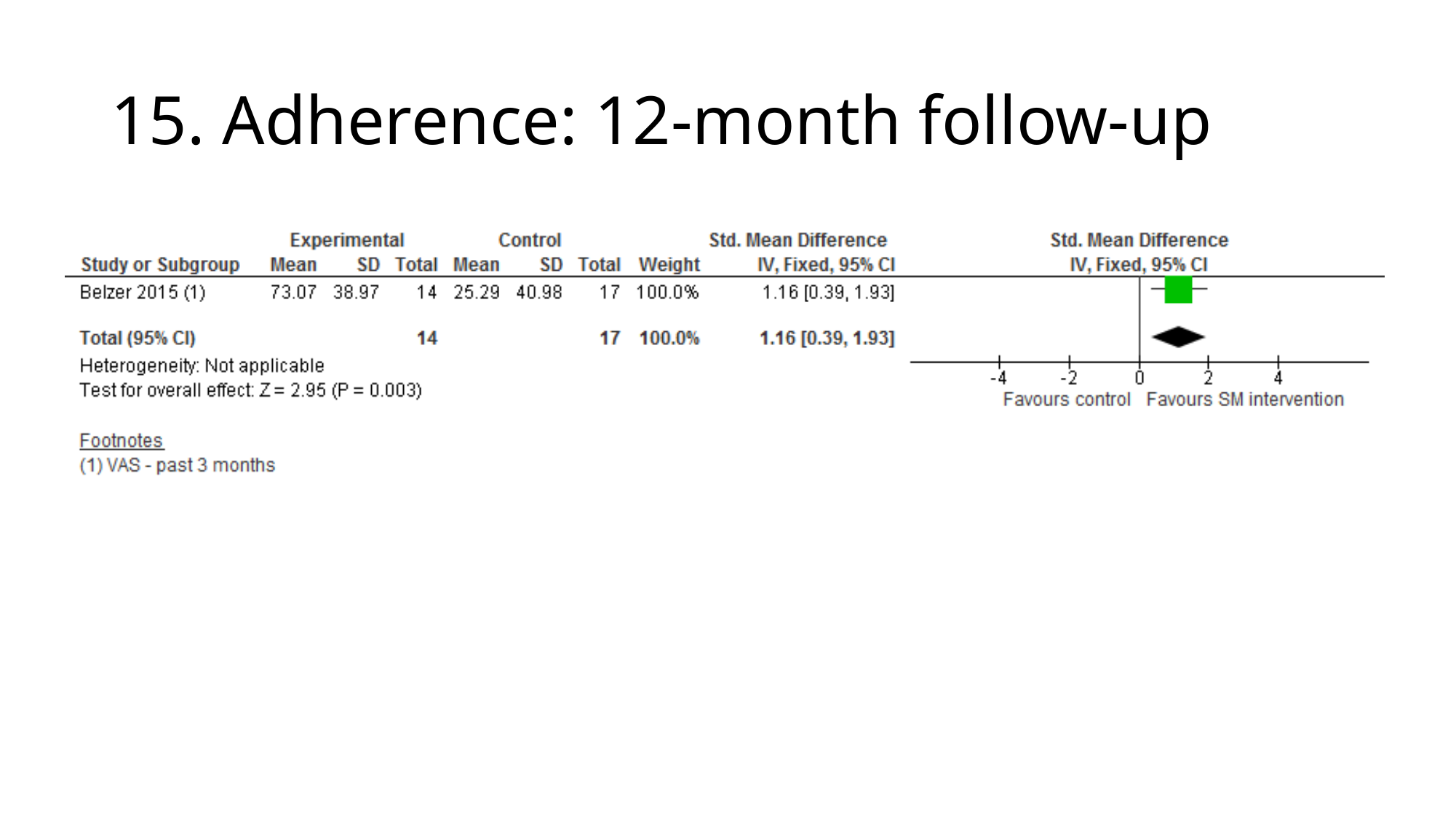

# 15. Adherence: 12-month follow-up

## Slide 17
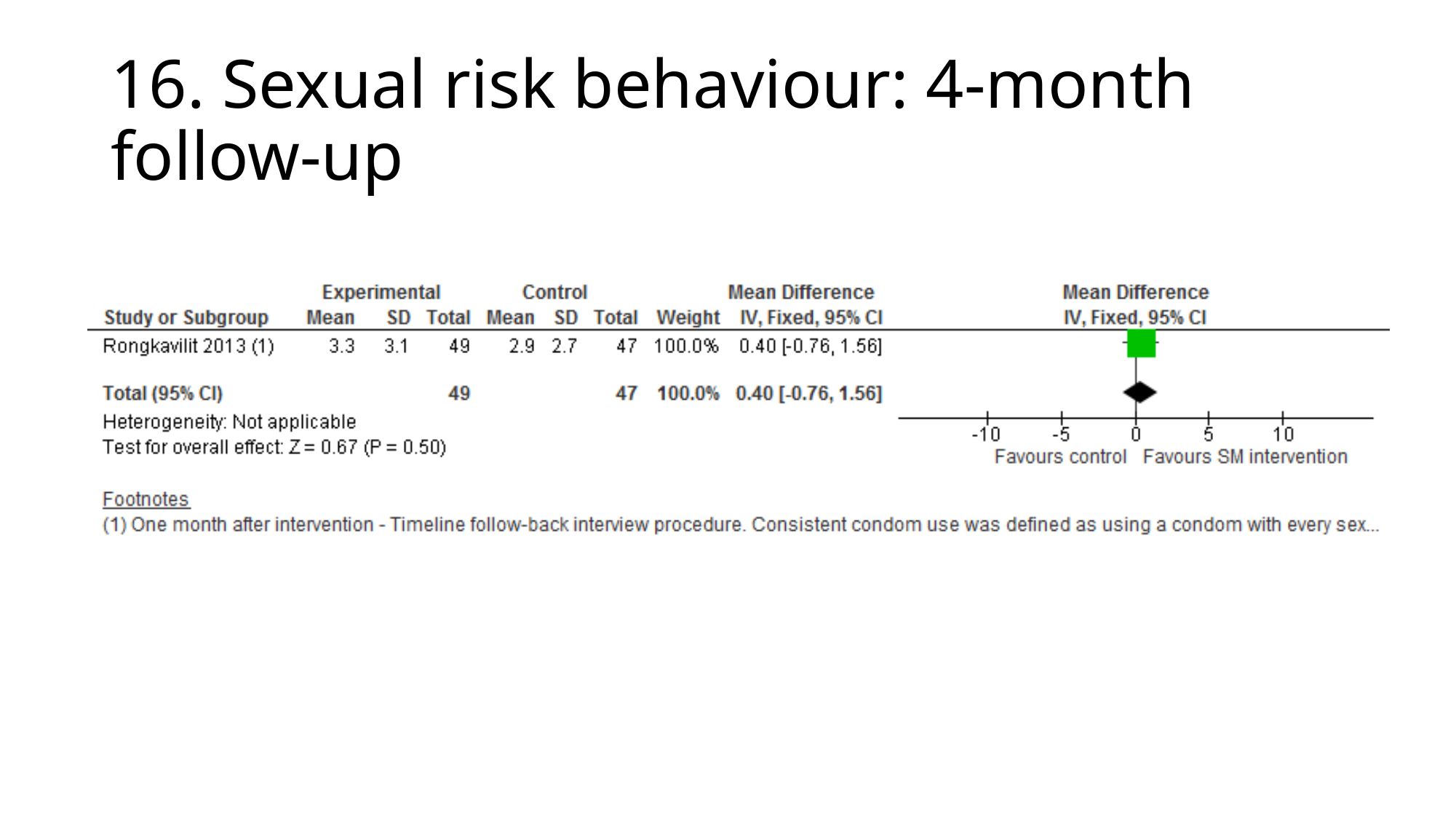

# 16. Sexual risk behaviour: 4-month follow-up

## Slide 18
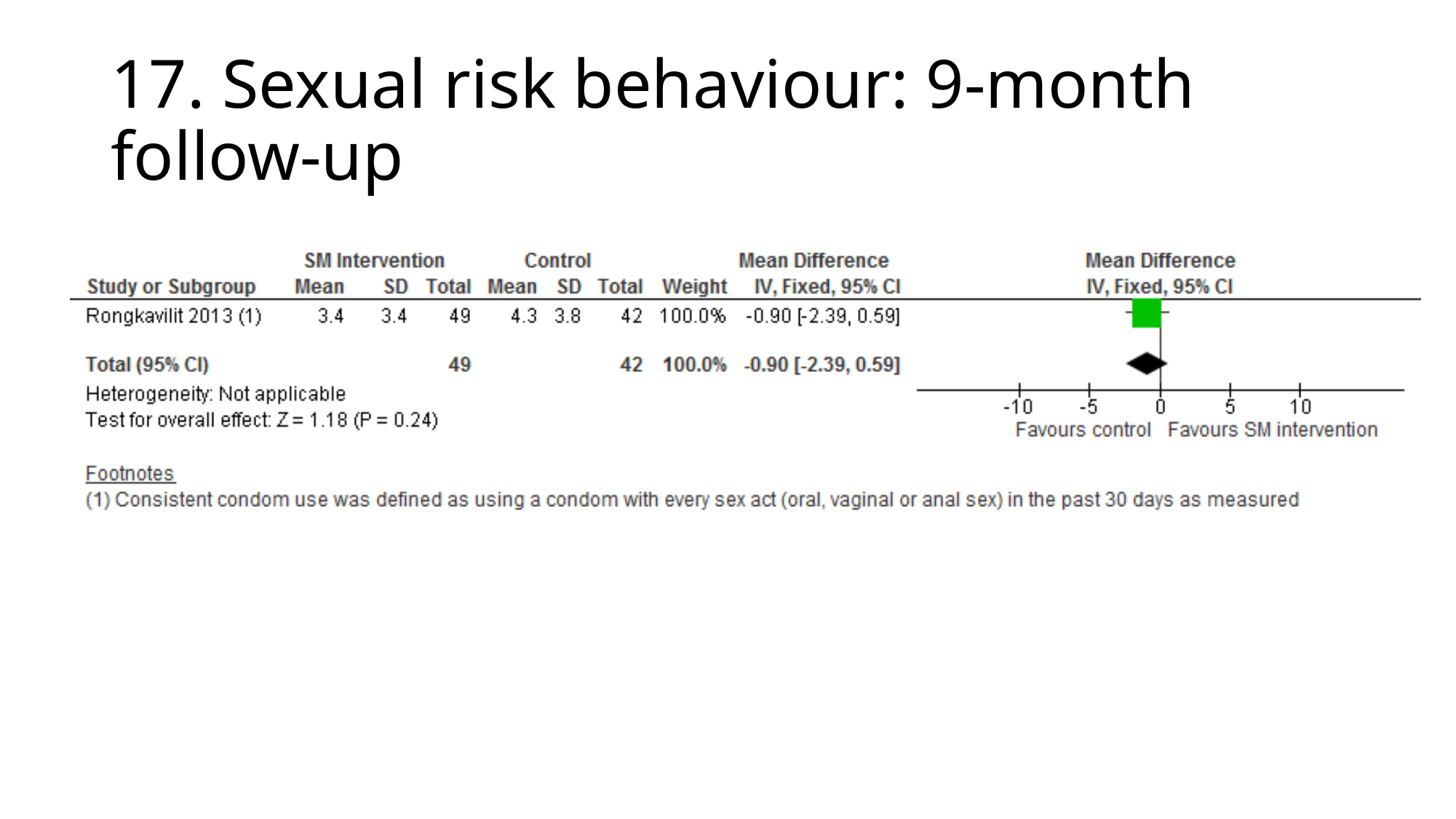

# 17. Sexual risk behaviour: 9-month follow-up

## Slide 19
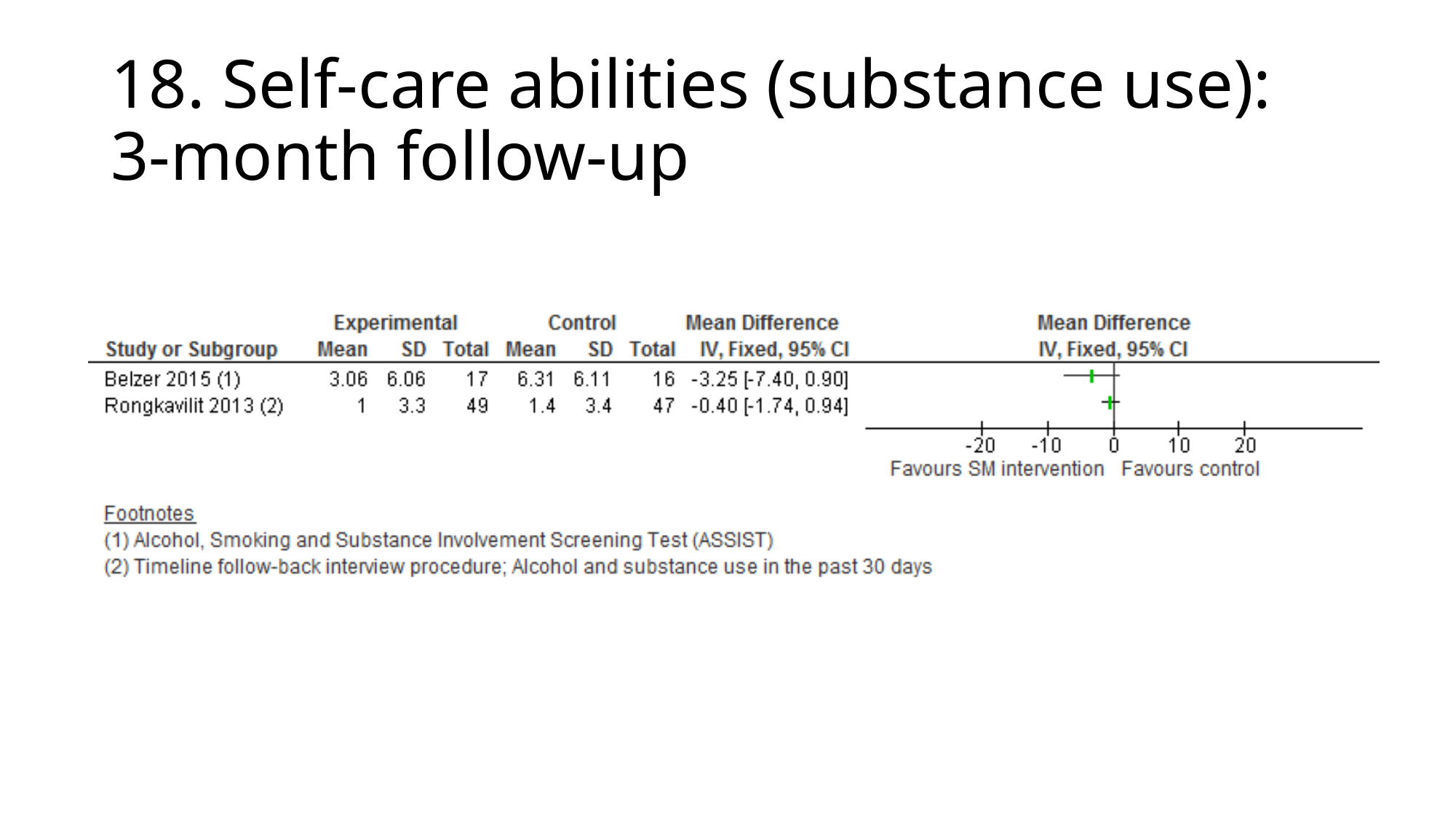

# 18. Self-care abilities (substance use): 3-month follow-up

## Slide 20
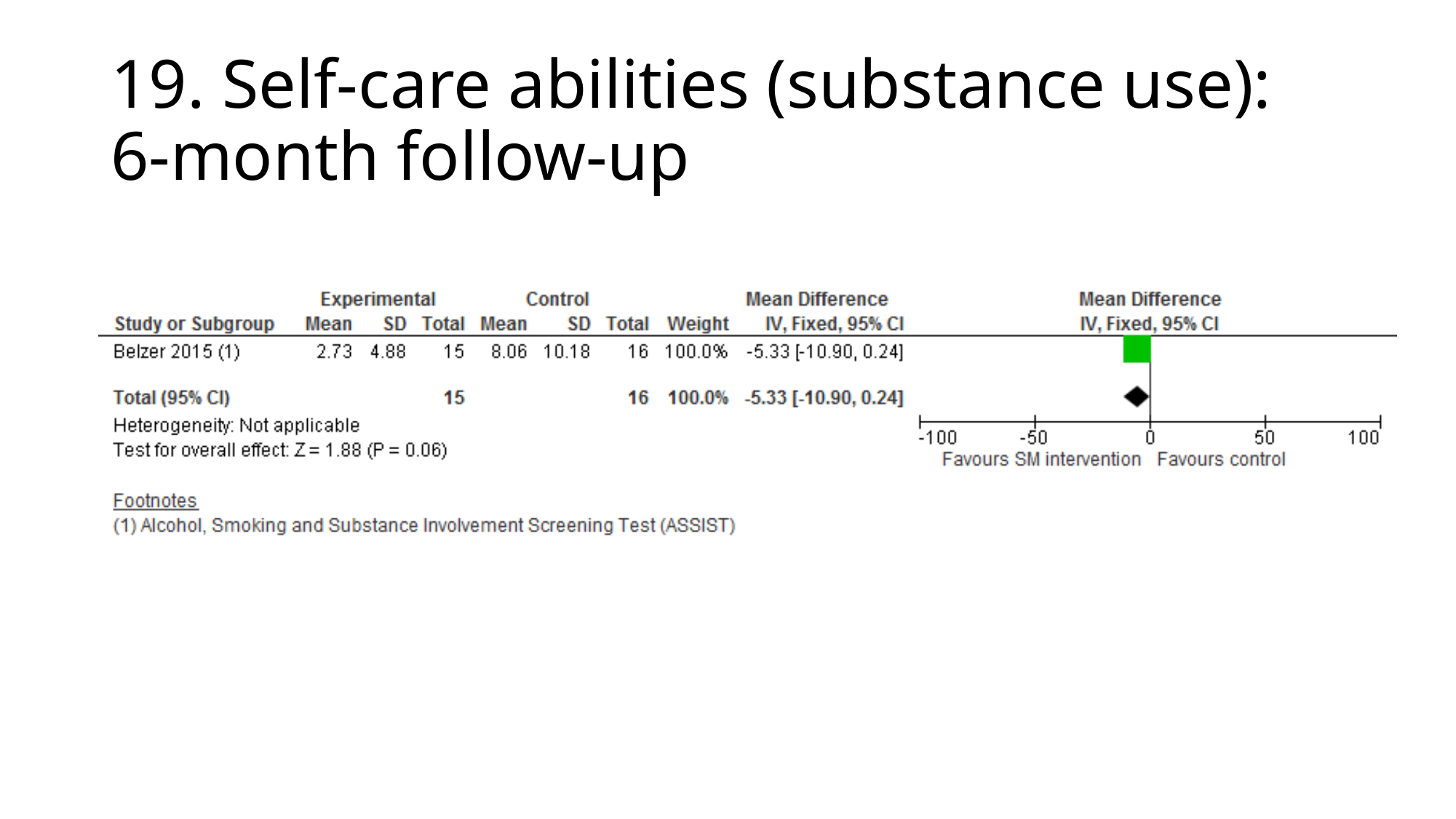

# 19. Self-care abilities (substance use): 6-month follow-up

## Slide 21
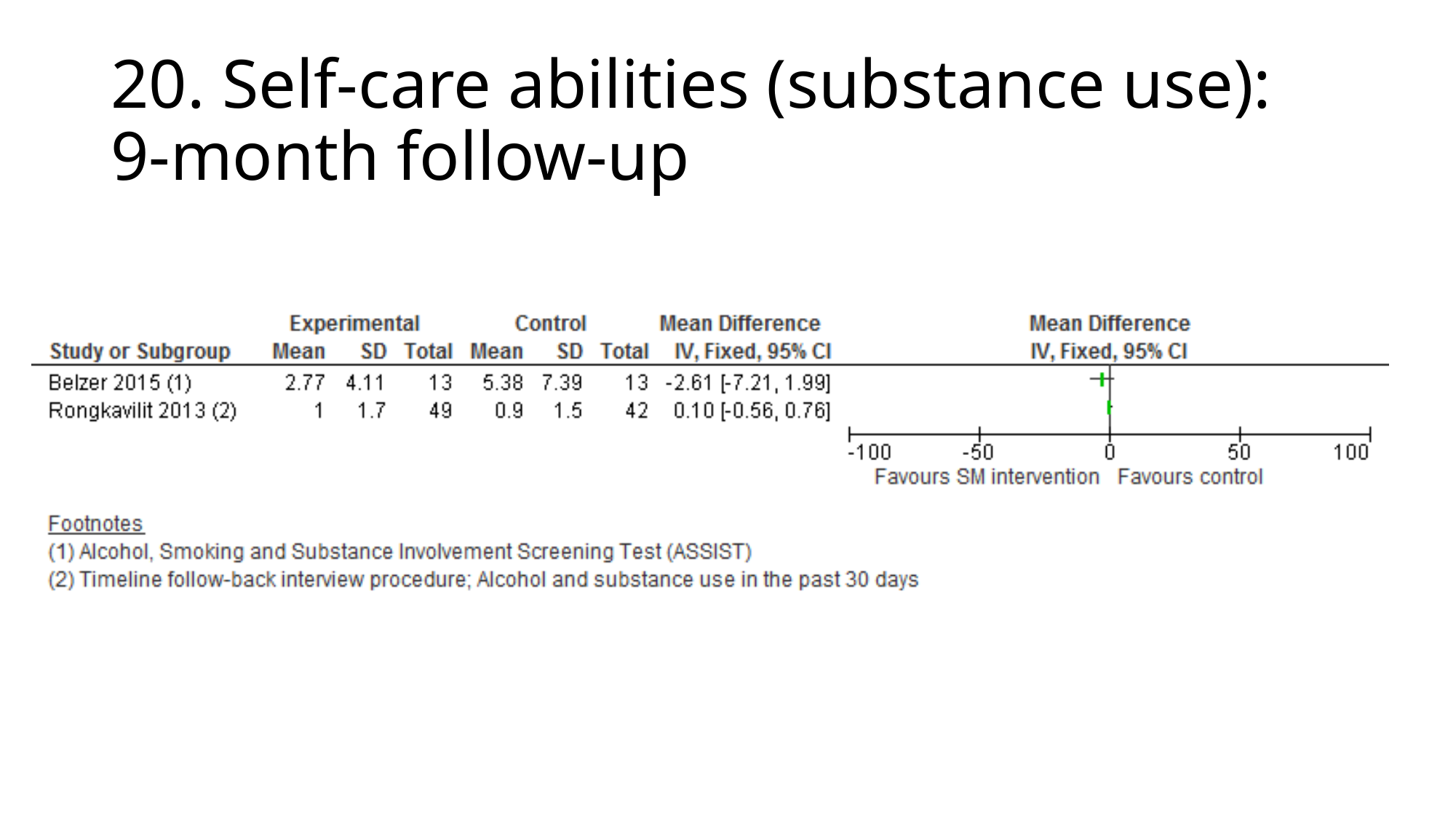

# 20. Self-care abilities (substance use): 9-month follow-up

## Slide 22
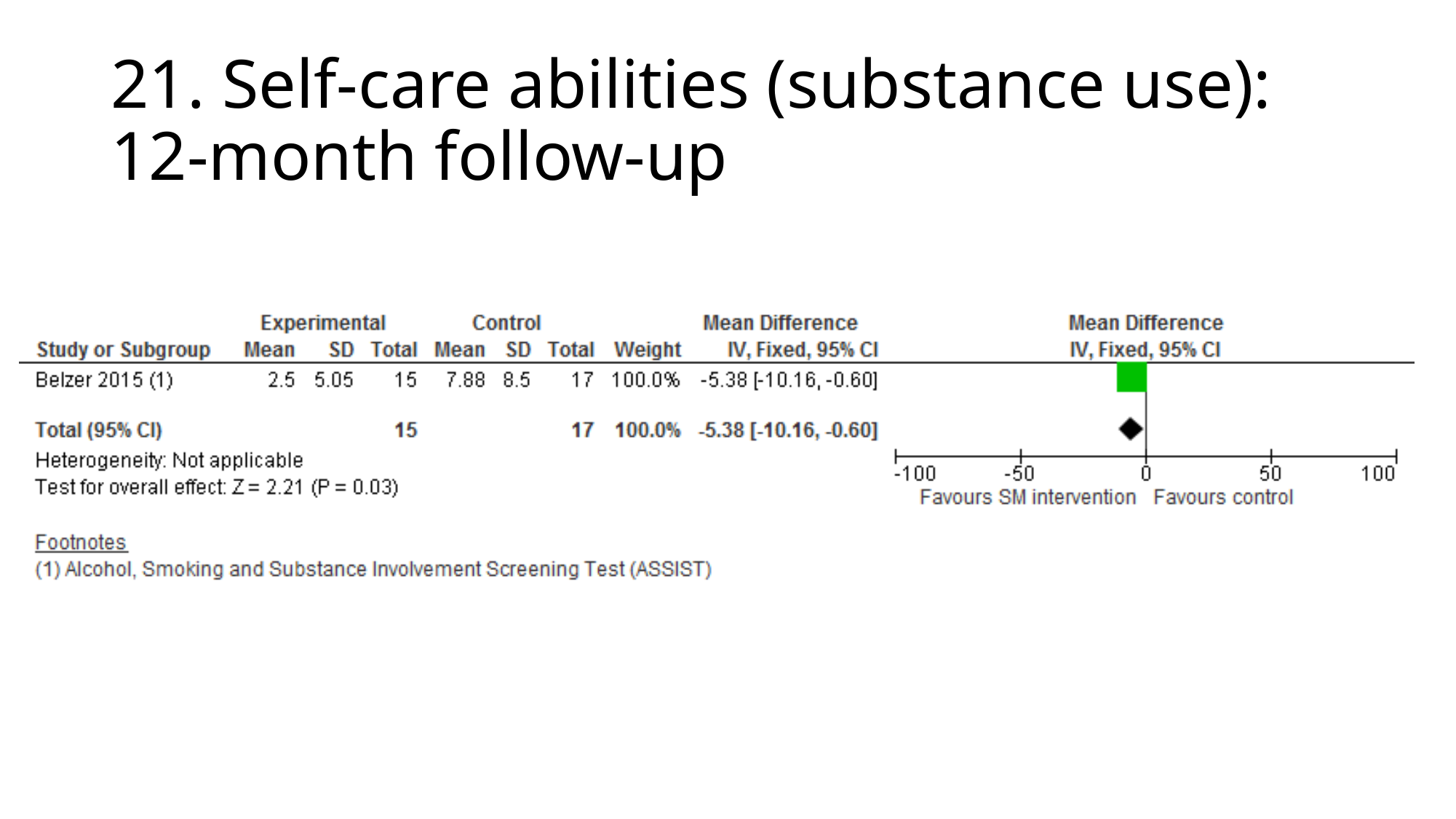

# 21. Self-care abilities (substance use): 12-month follow-up

## Slide 23
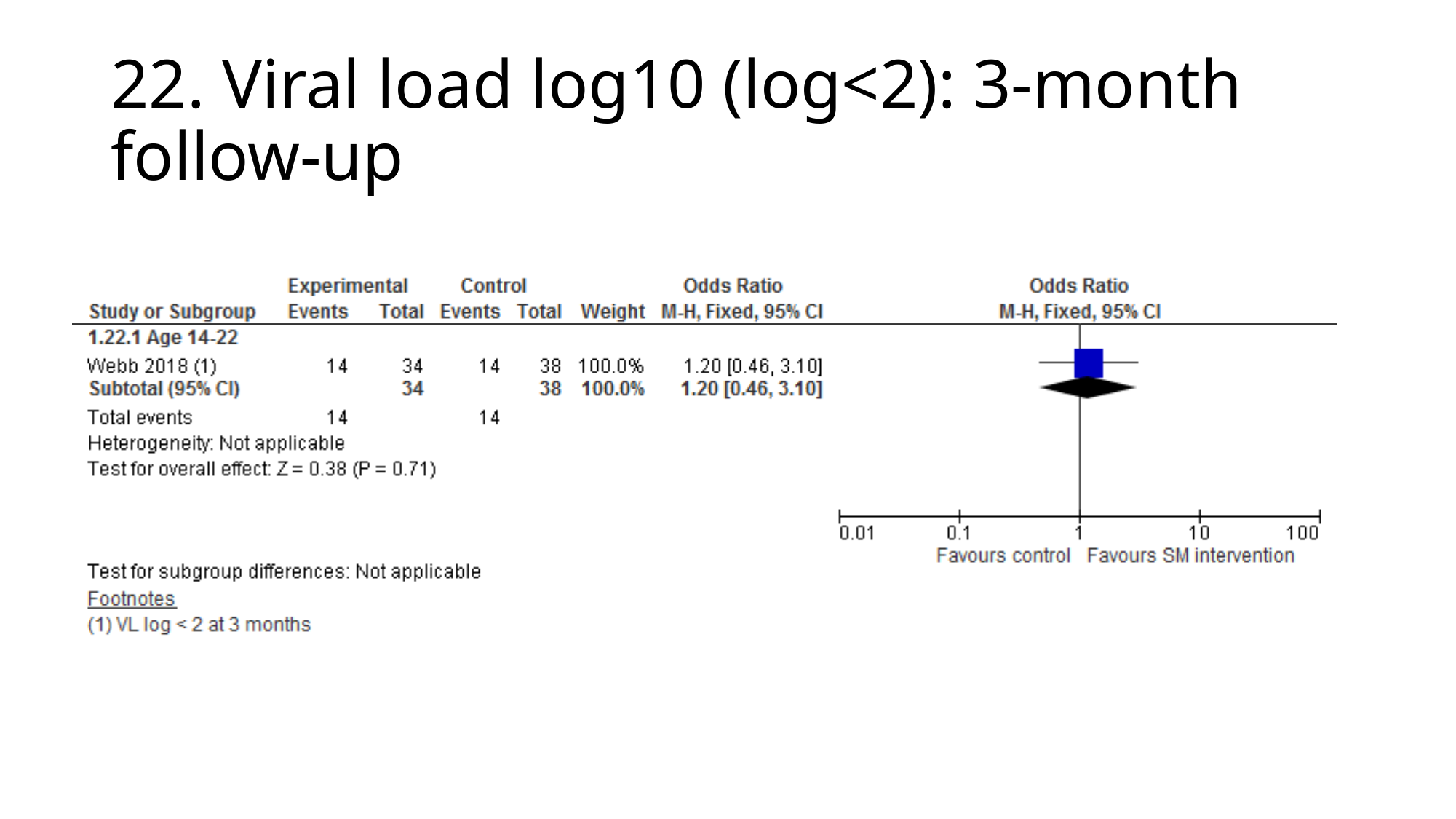

# 22. Viral load log10 (log<2): 3-month follow-up

## Slide 24
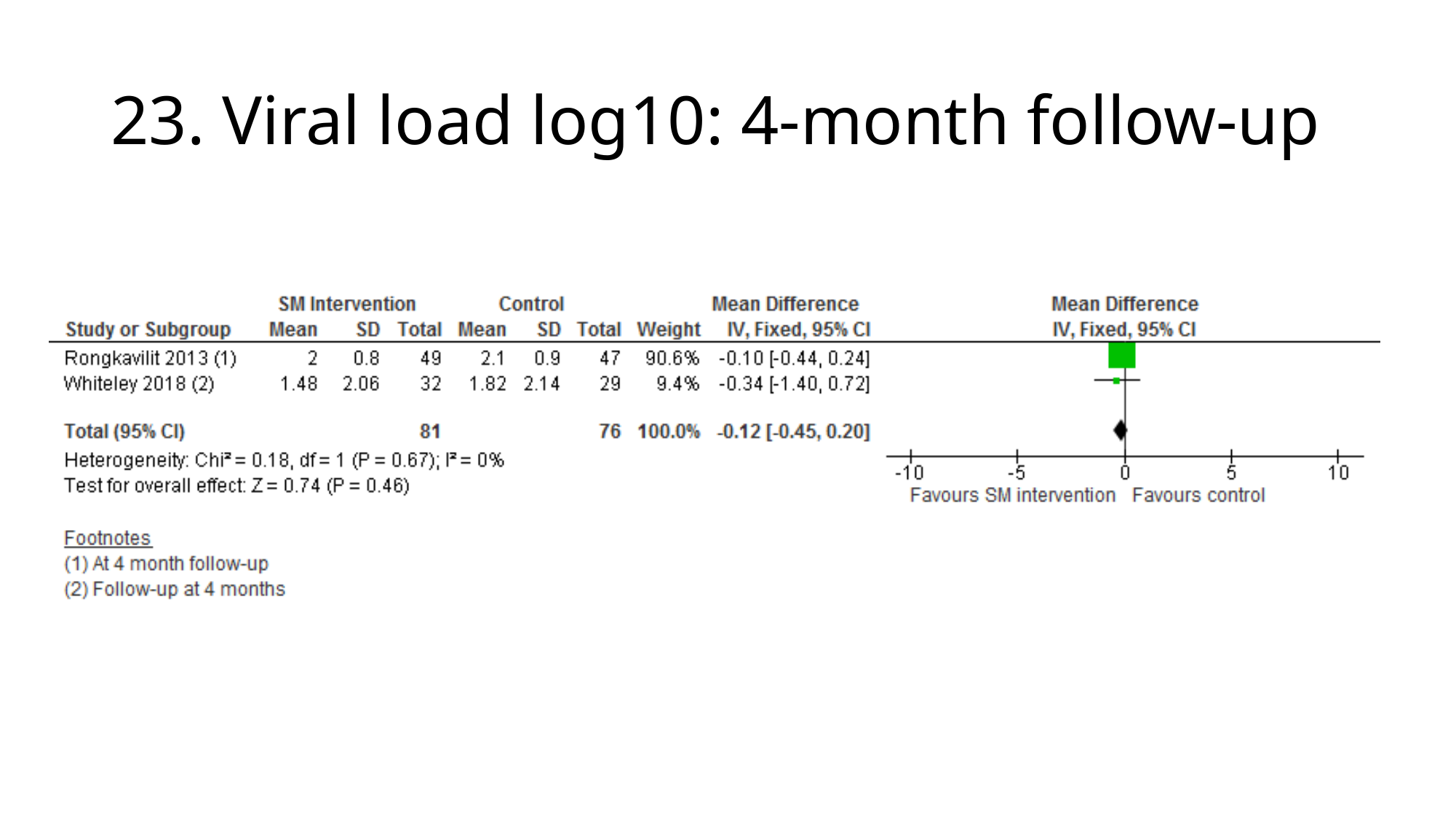

# 23. Viral load log10: 4-month follow-up

## Slide 25
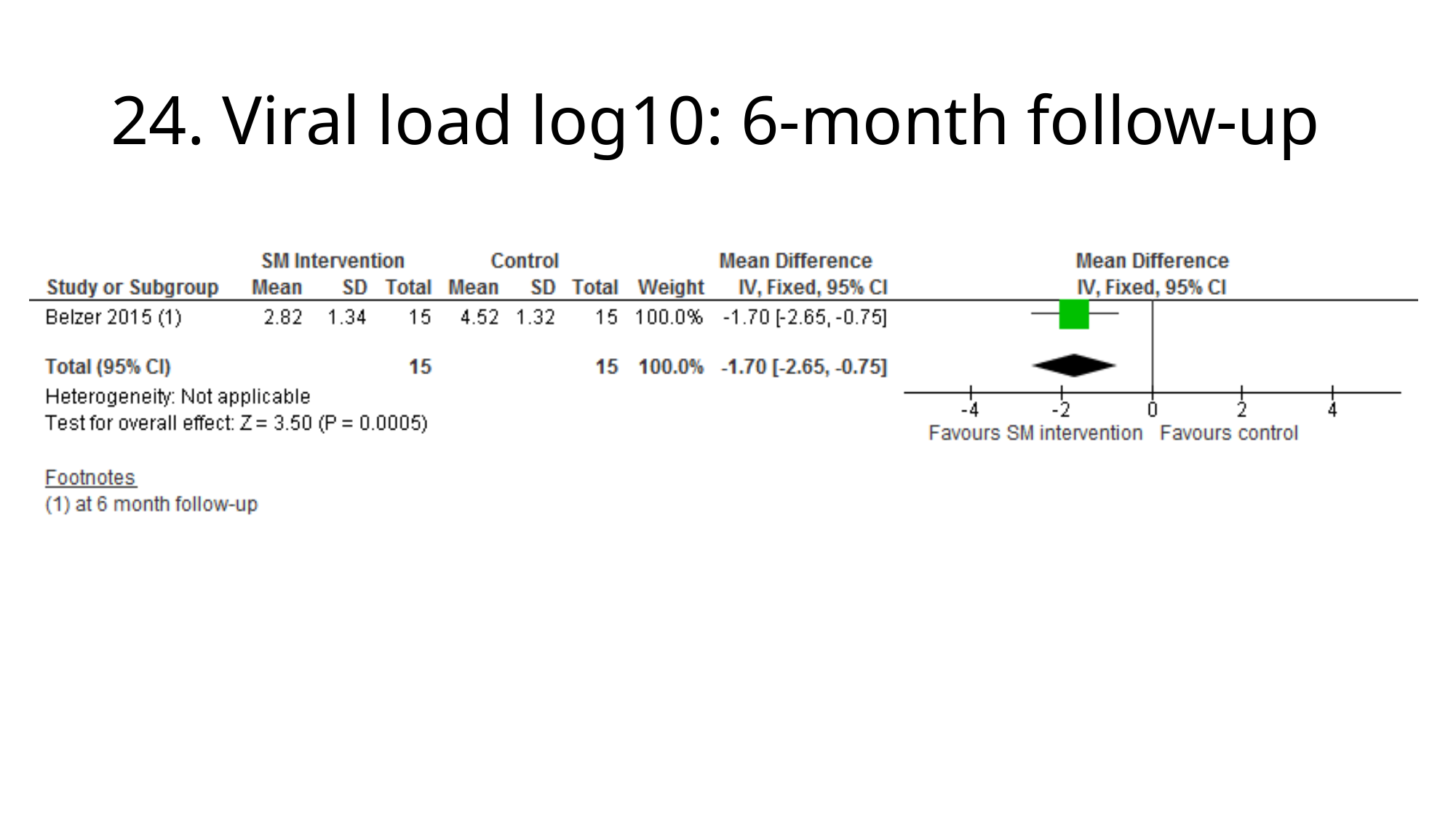

# 24. Viral load log10: 6-month follow-up

## Slide 26
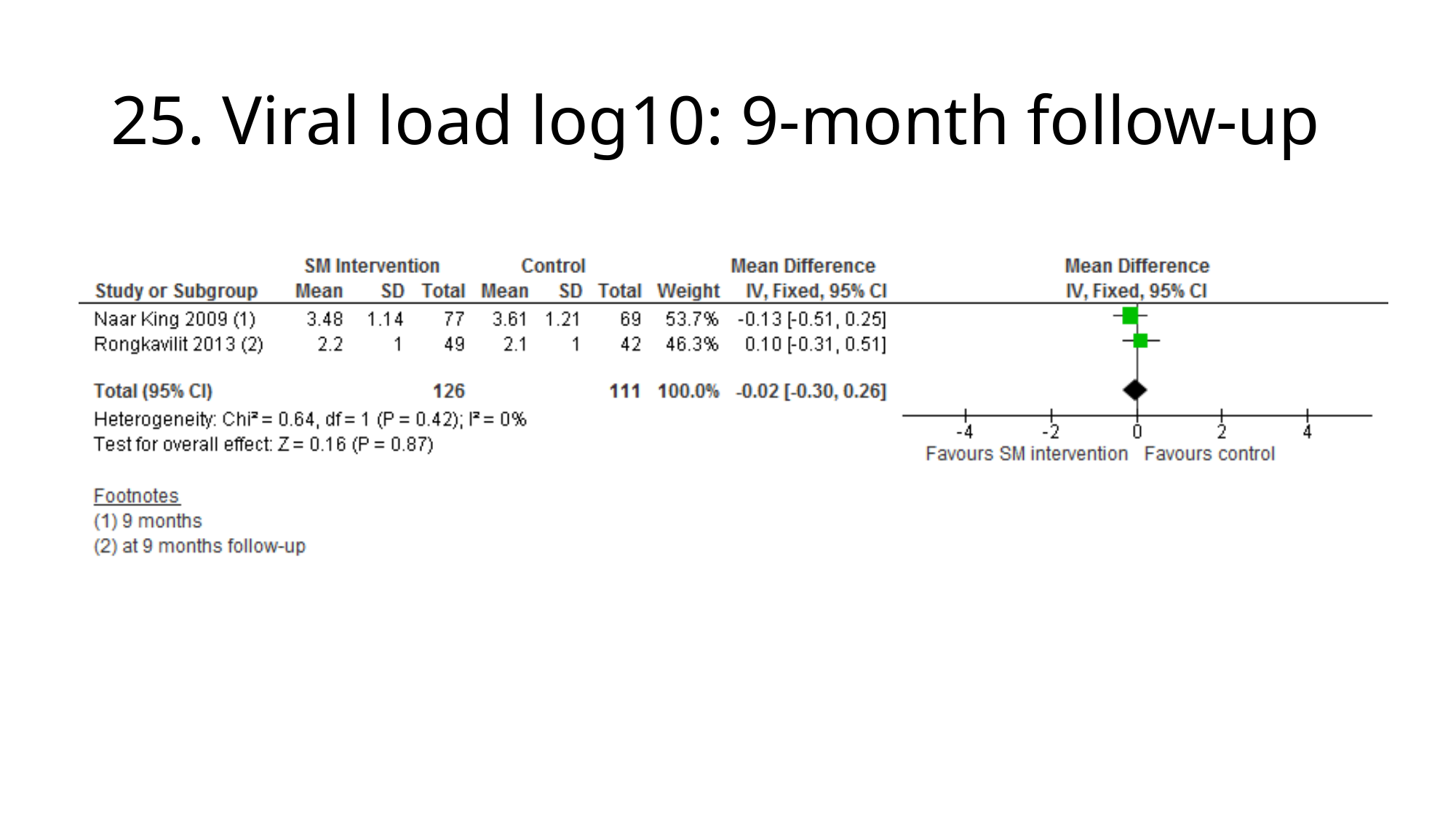

# 25. Viral load log10: 9-month follow-up

## Slide 27
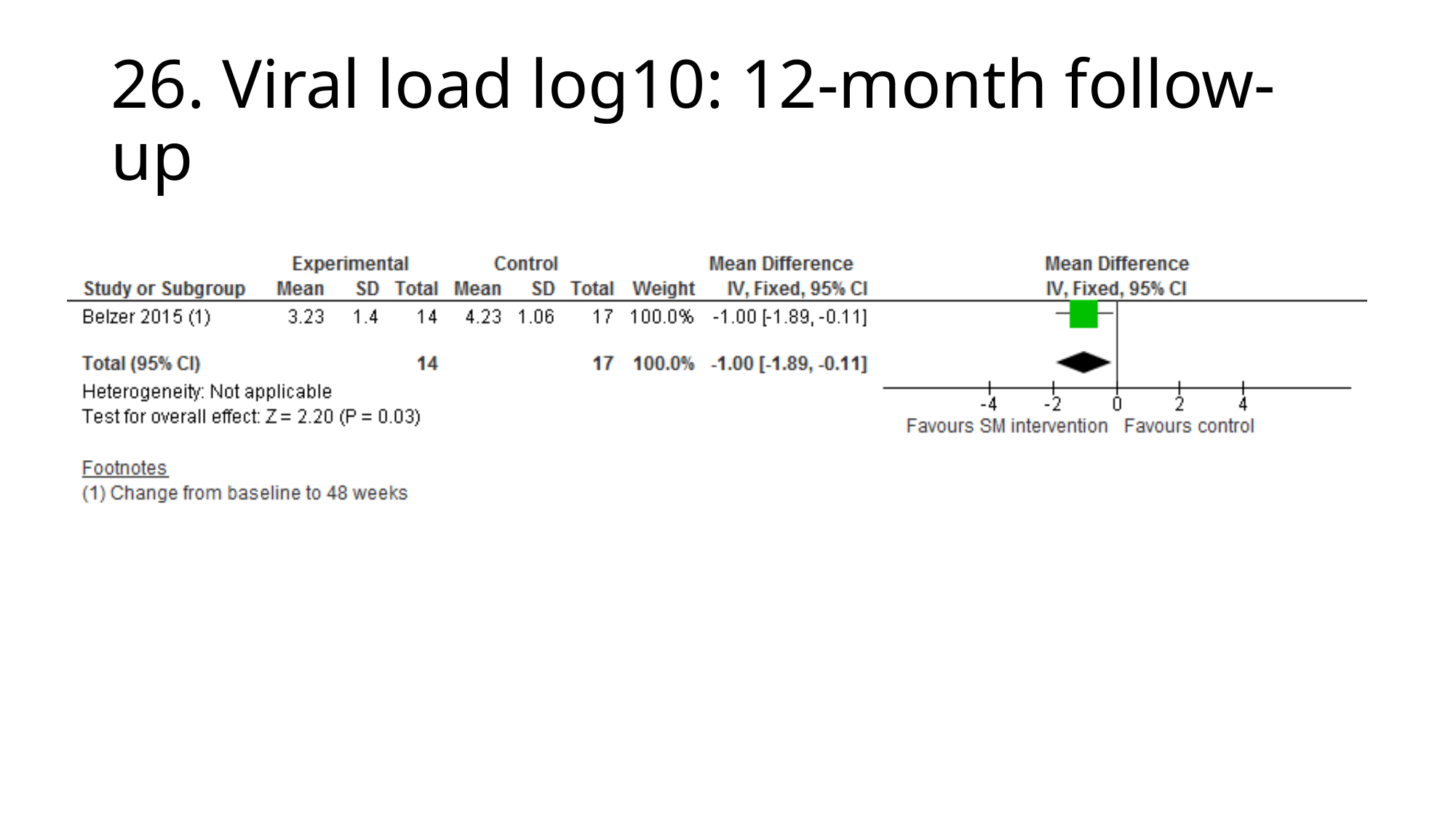

# 26. Viral load log10: 12-month follow-up

## Slide 28
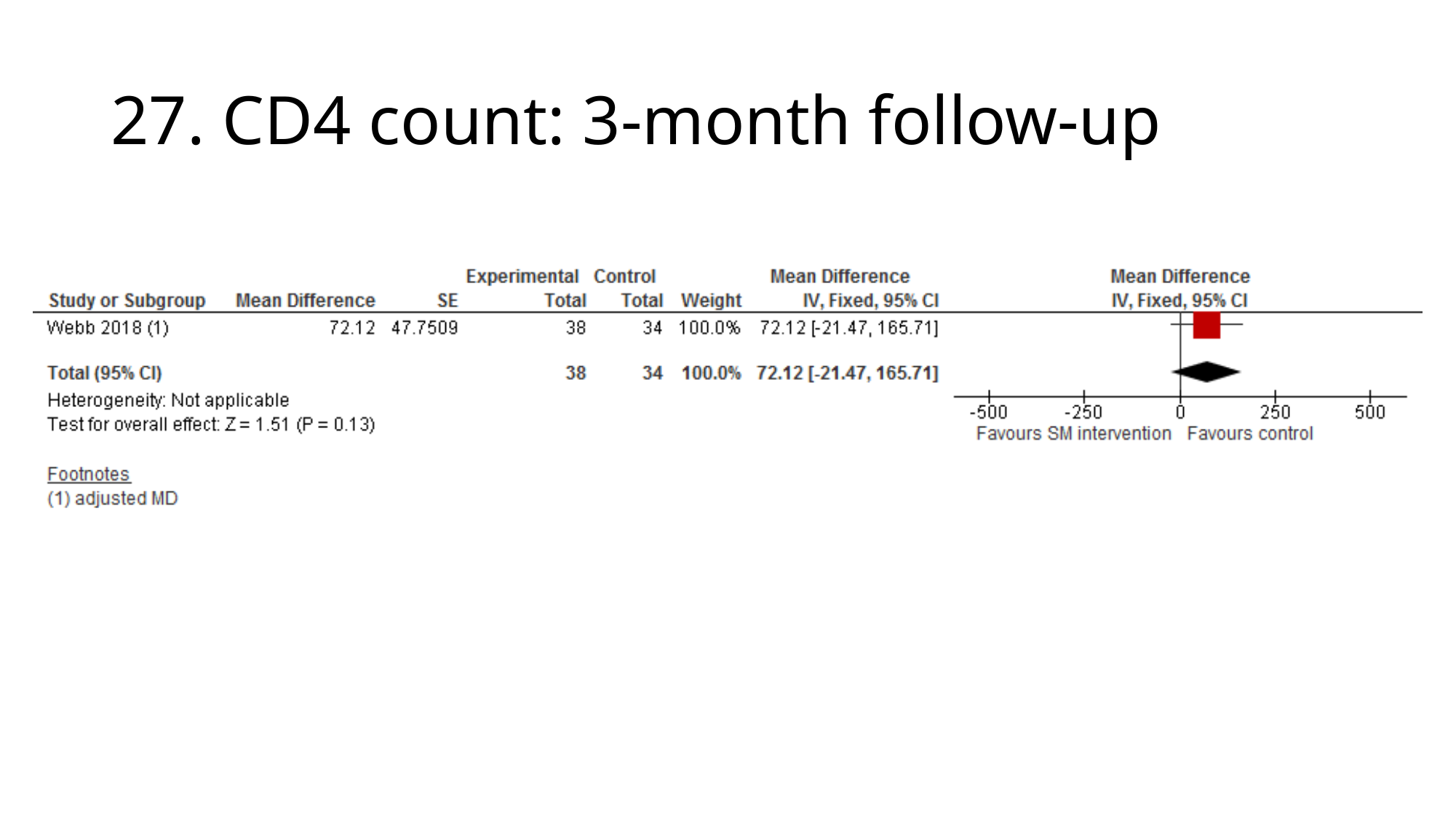

# 27. CD4 count: 3-month follow-up

## Slide 29
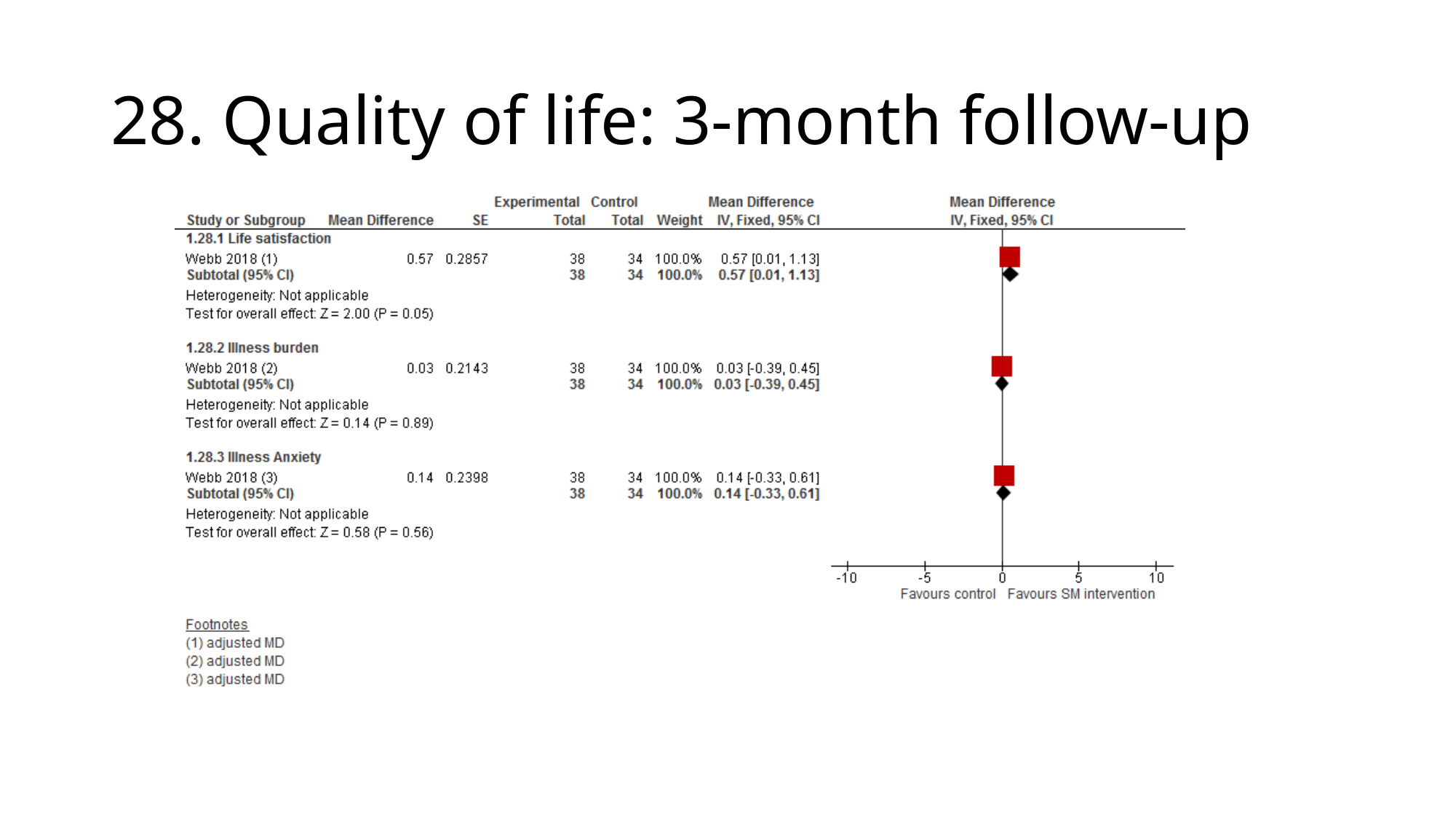

# 28. Quality of life: 3-month follow-up

## Slide 30
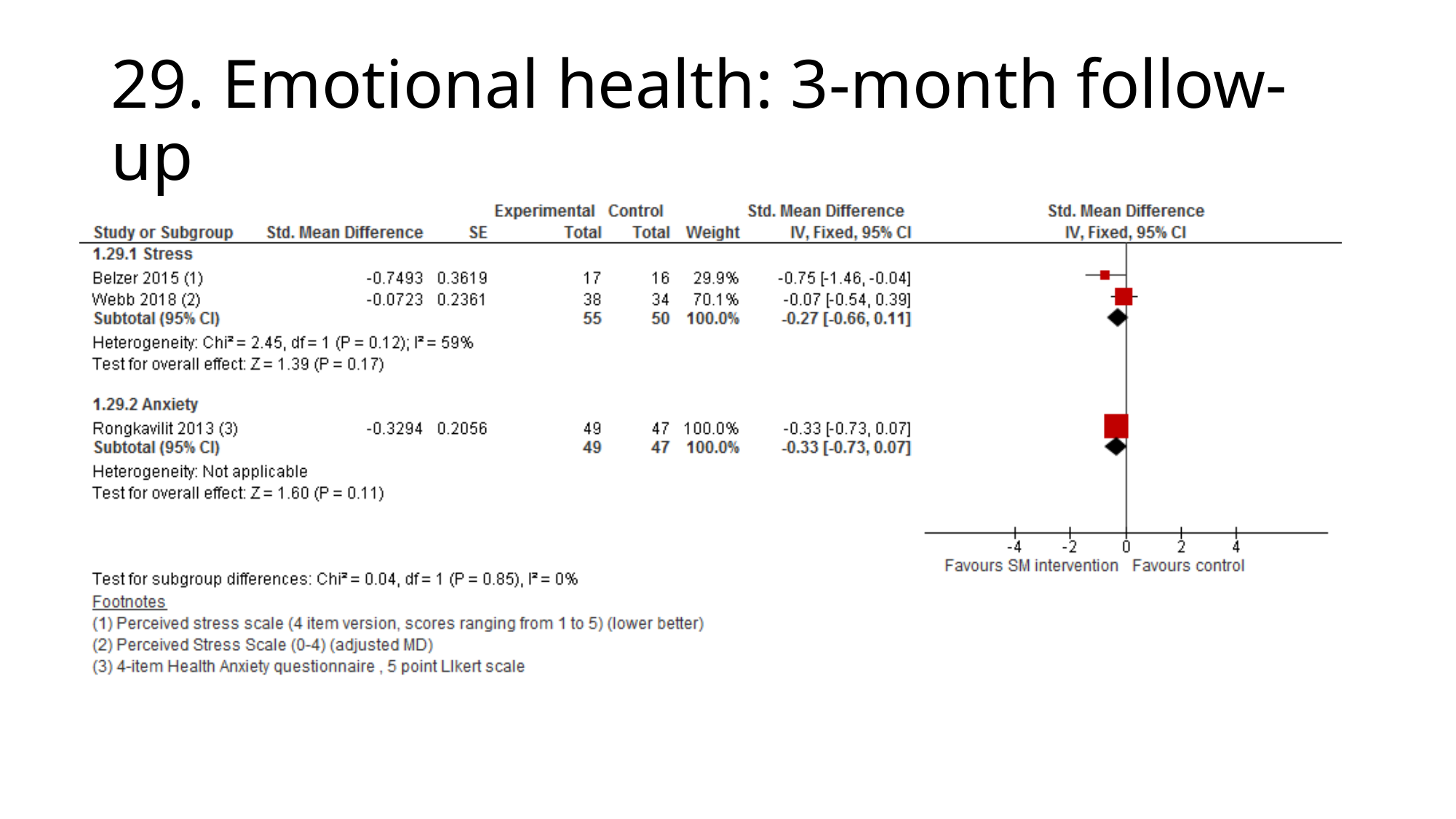

# 29. Emotional health: 3-month follow-up

## Slide 31
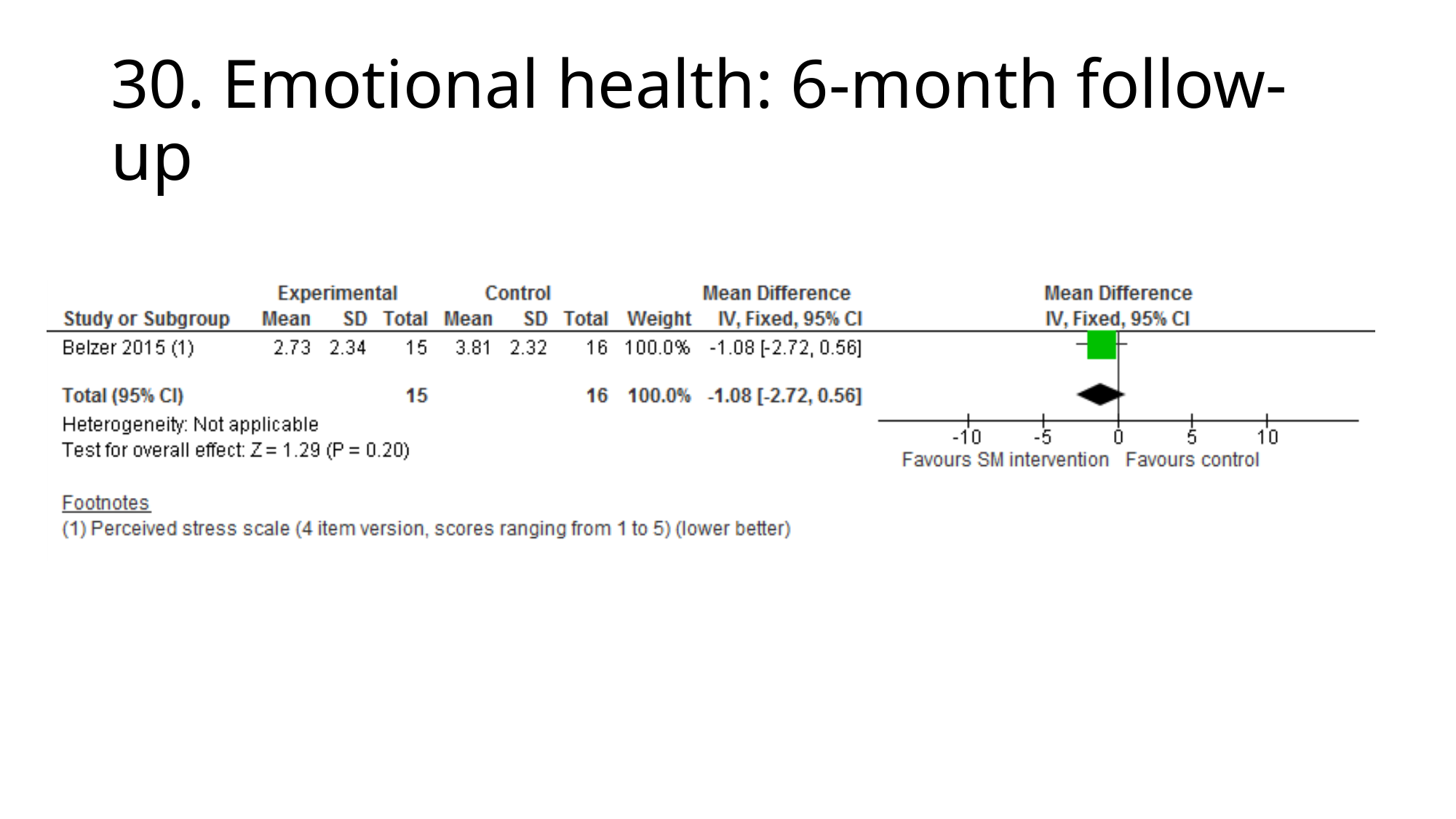

# 30. Emotional health: 6-month follow-up

## Slide 32
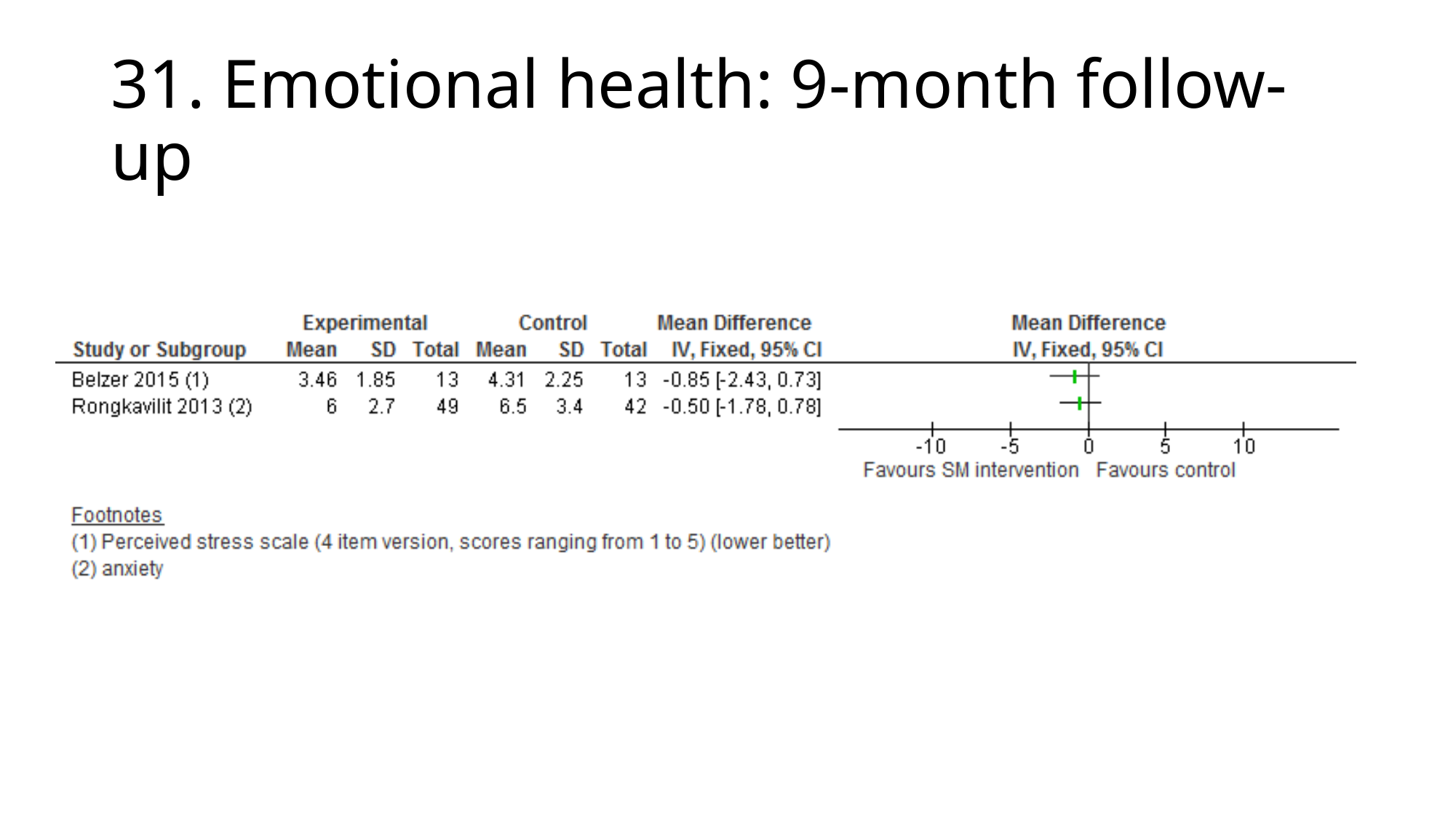

# 31. Emotional health: 9-month follow-up

## Slide 33
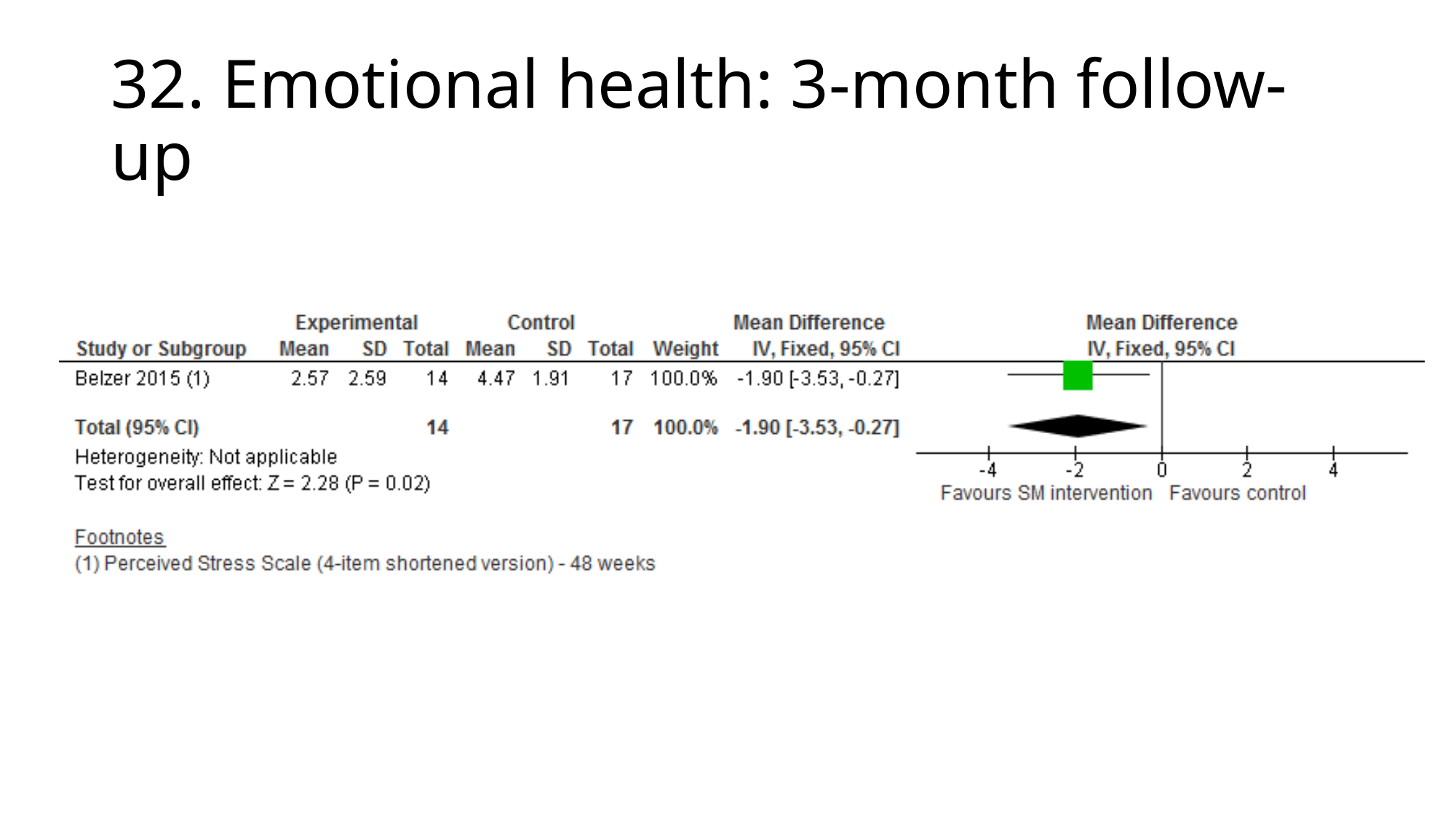

# 32. Emotional health: 3-month follow-up

## Slide 34
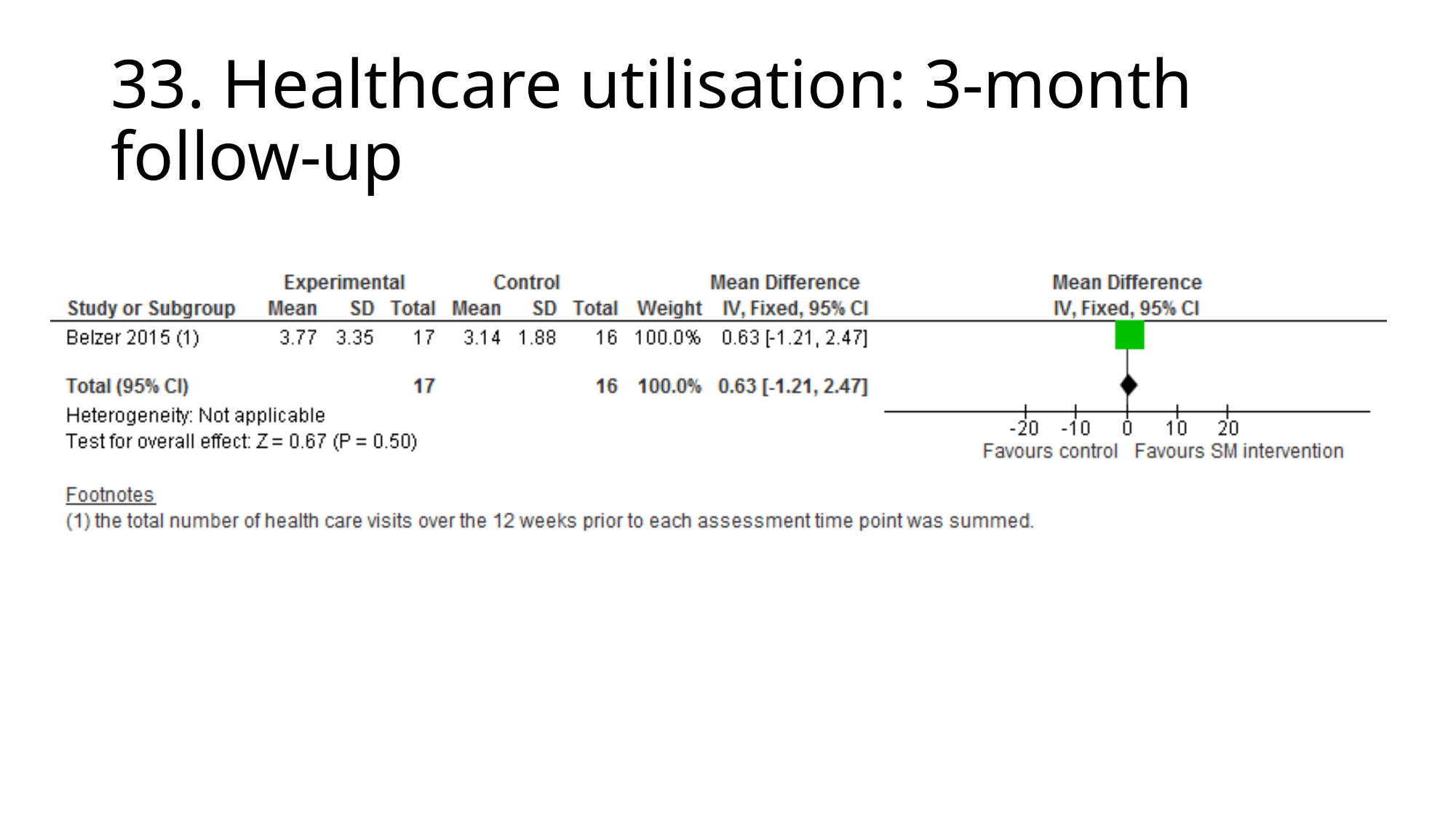

# 33. Healthcare utilisation: 3-month follow-up

## Slide 35
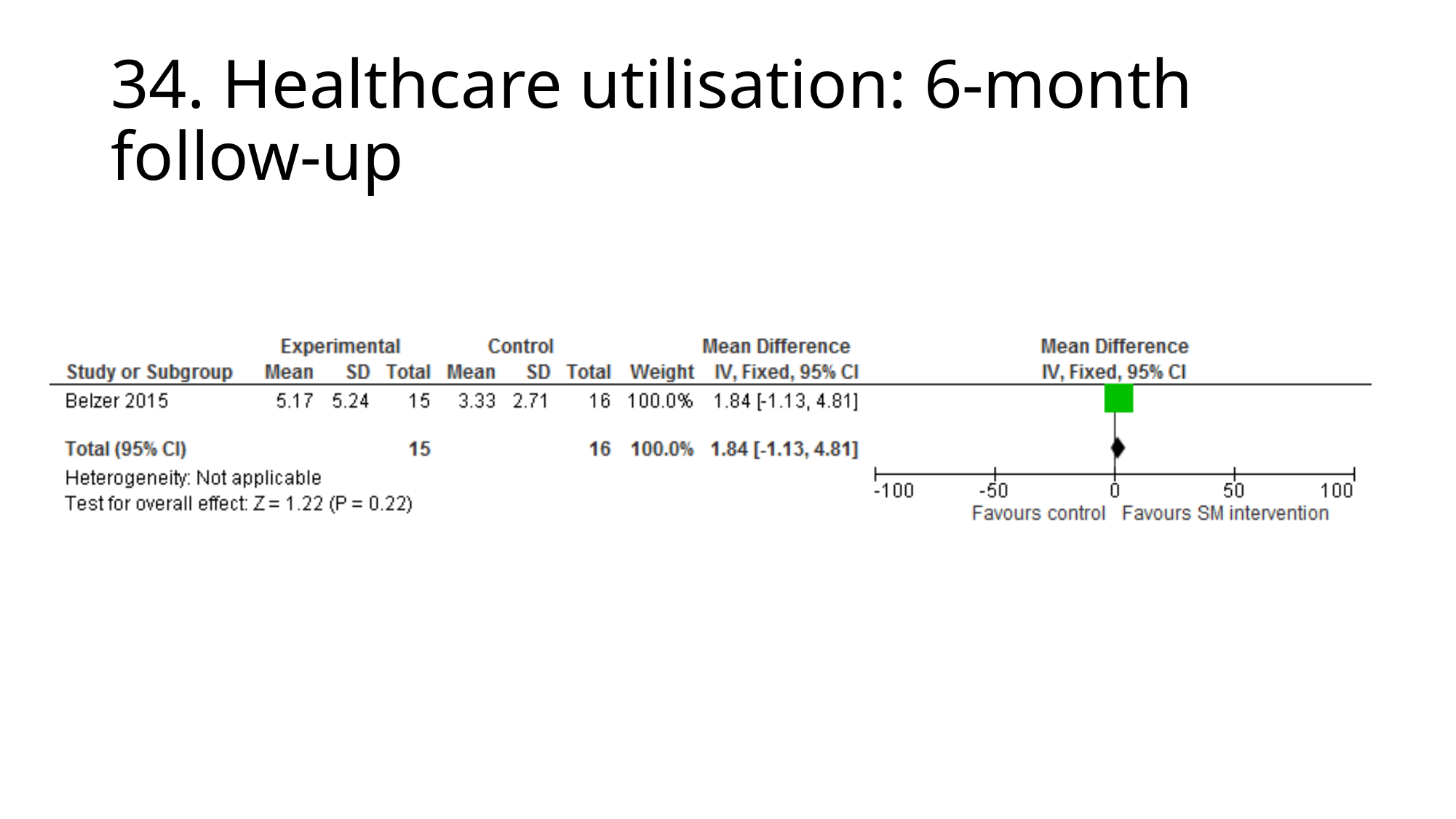

# 34. Healthcare utilisation: 6-month follow-up

## Slide 36
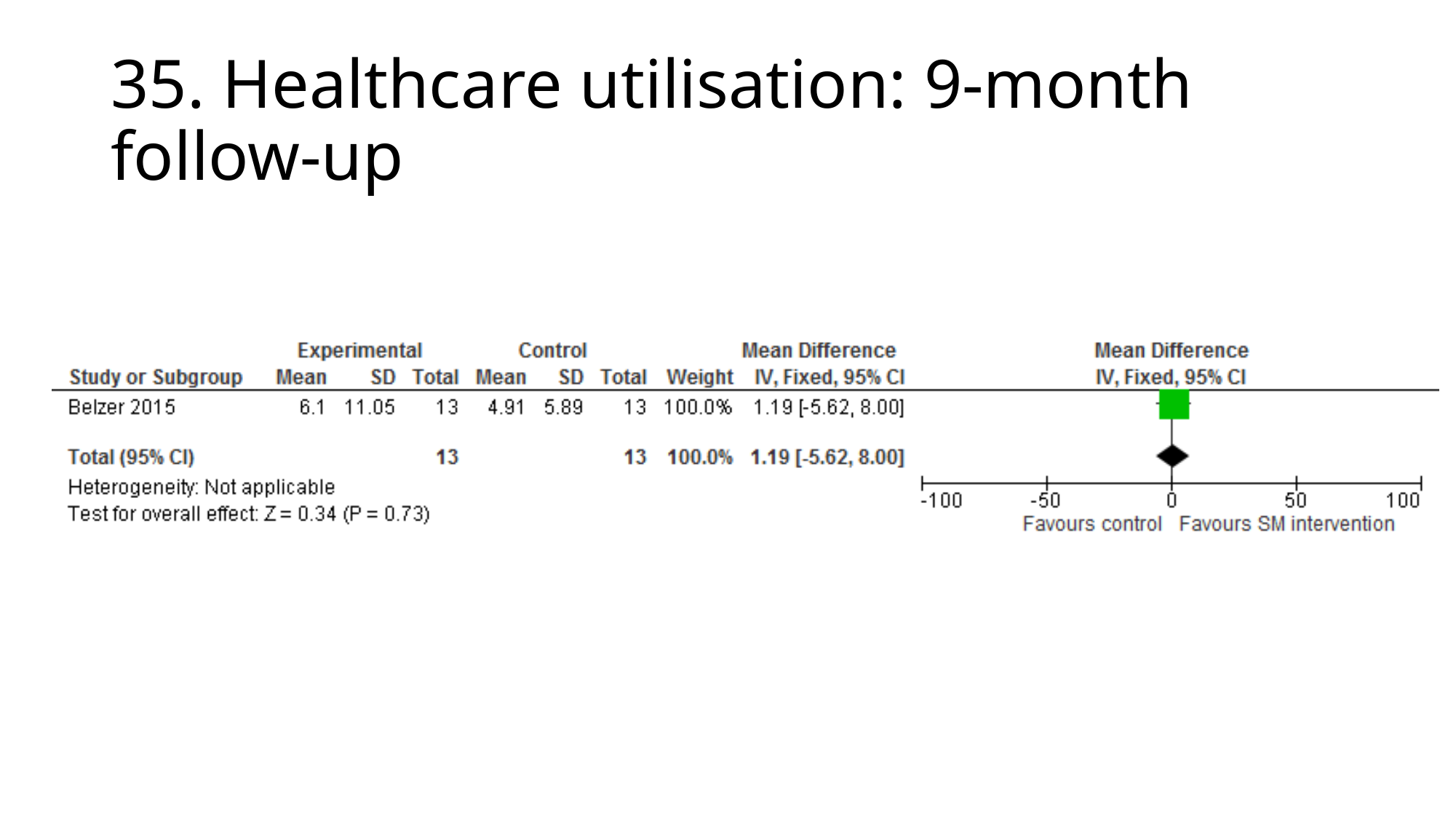

# 35. Healthcare utilisation: 9-month follow-up

## Slide 37
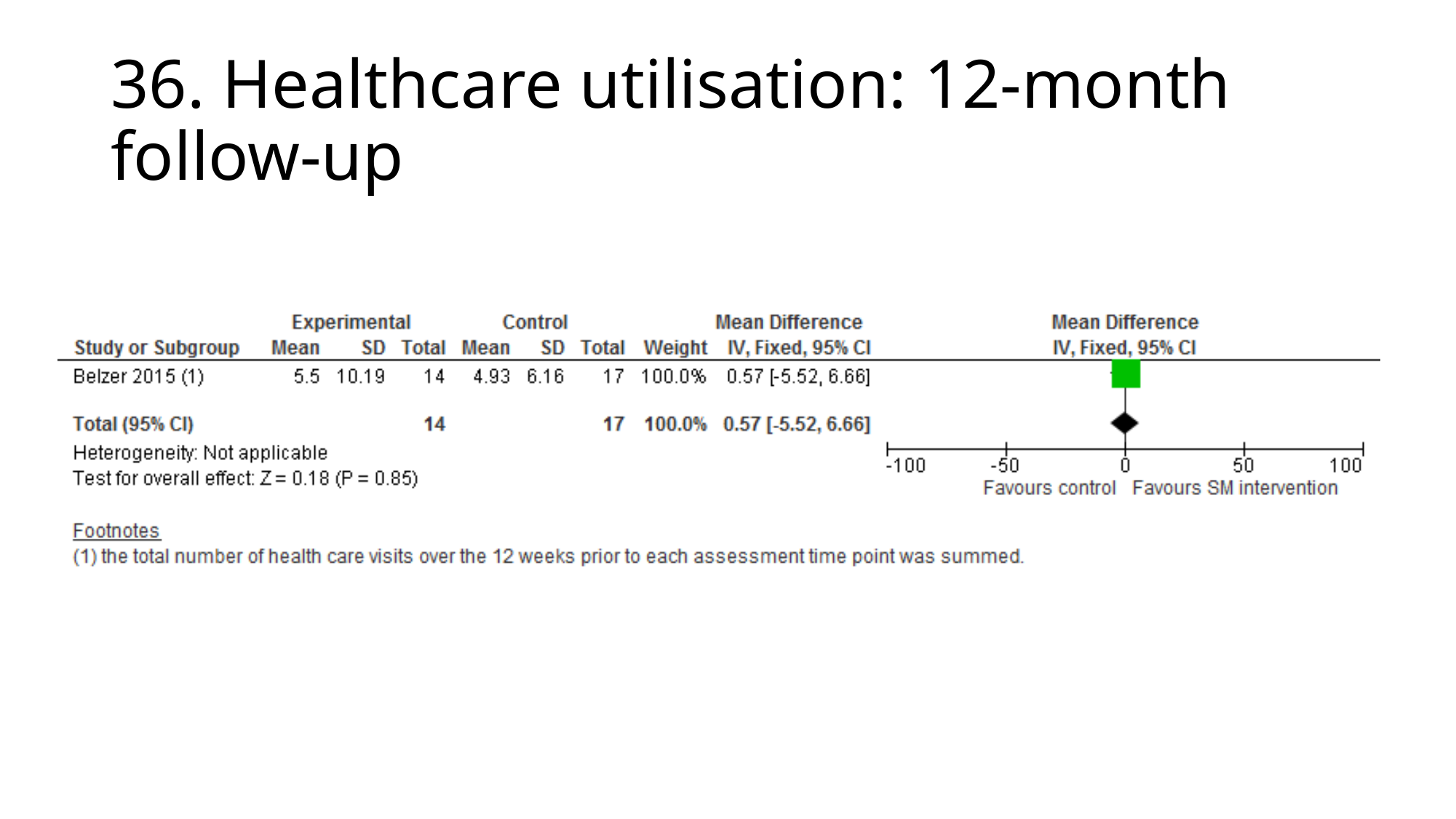

# 36. Healthcare utilisation: 12-month follow-up

## Slide 38
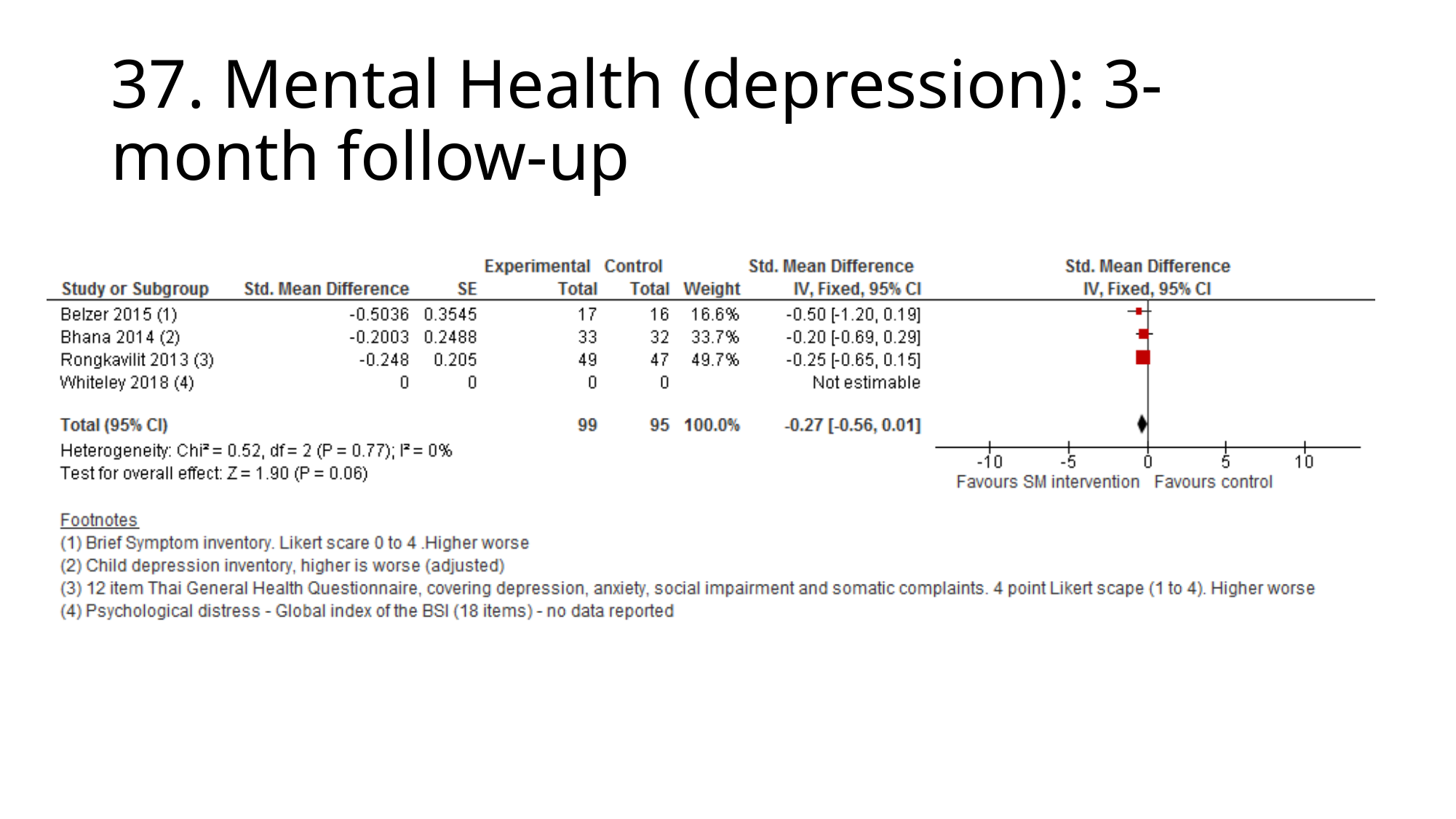

# 37. Mental Health (depression): 3-month follow-up

## Slide 39
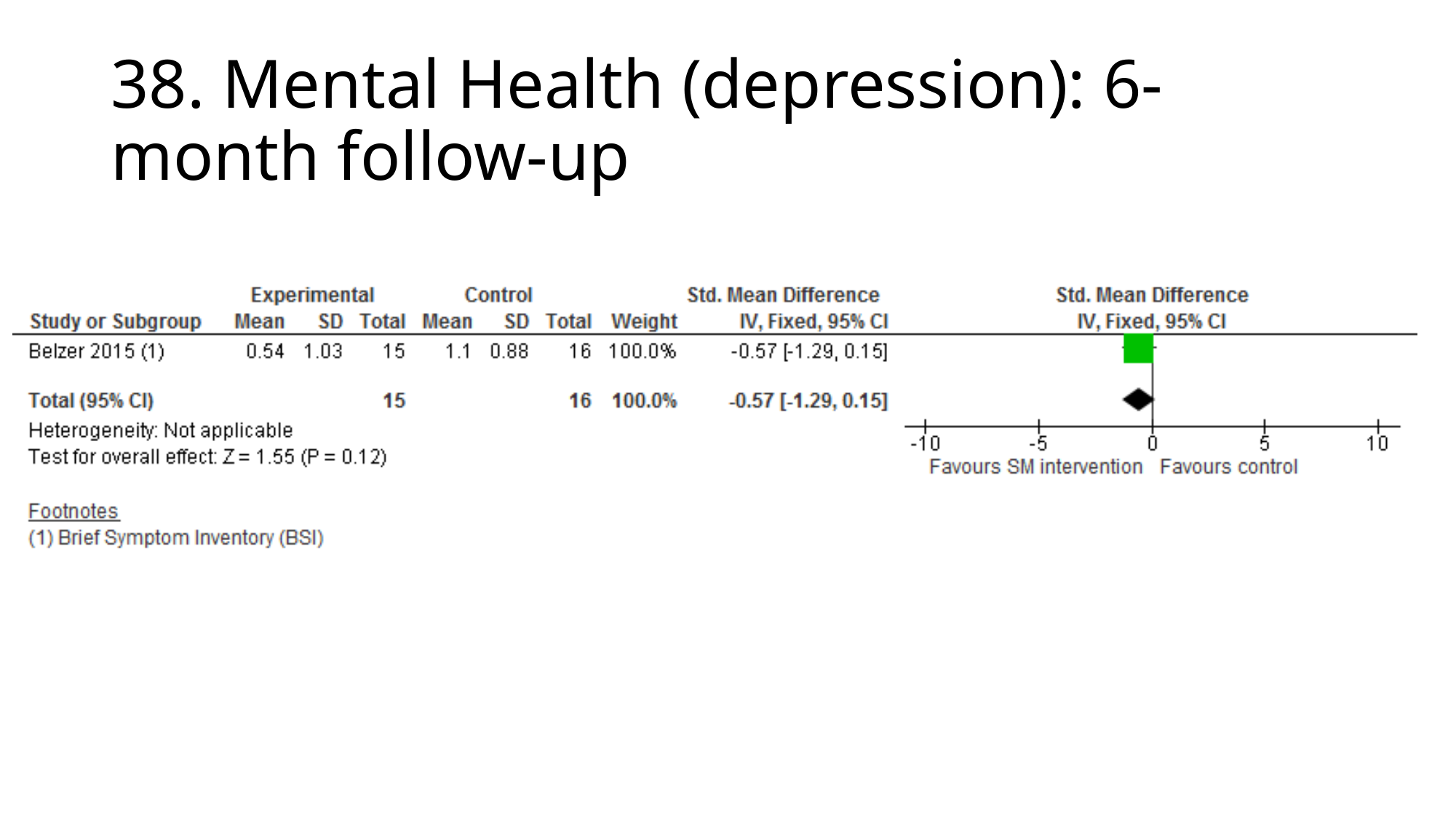

# 38. Mental Health (depression): 6-month follow-up

## Slide 40
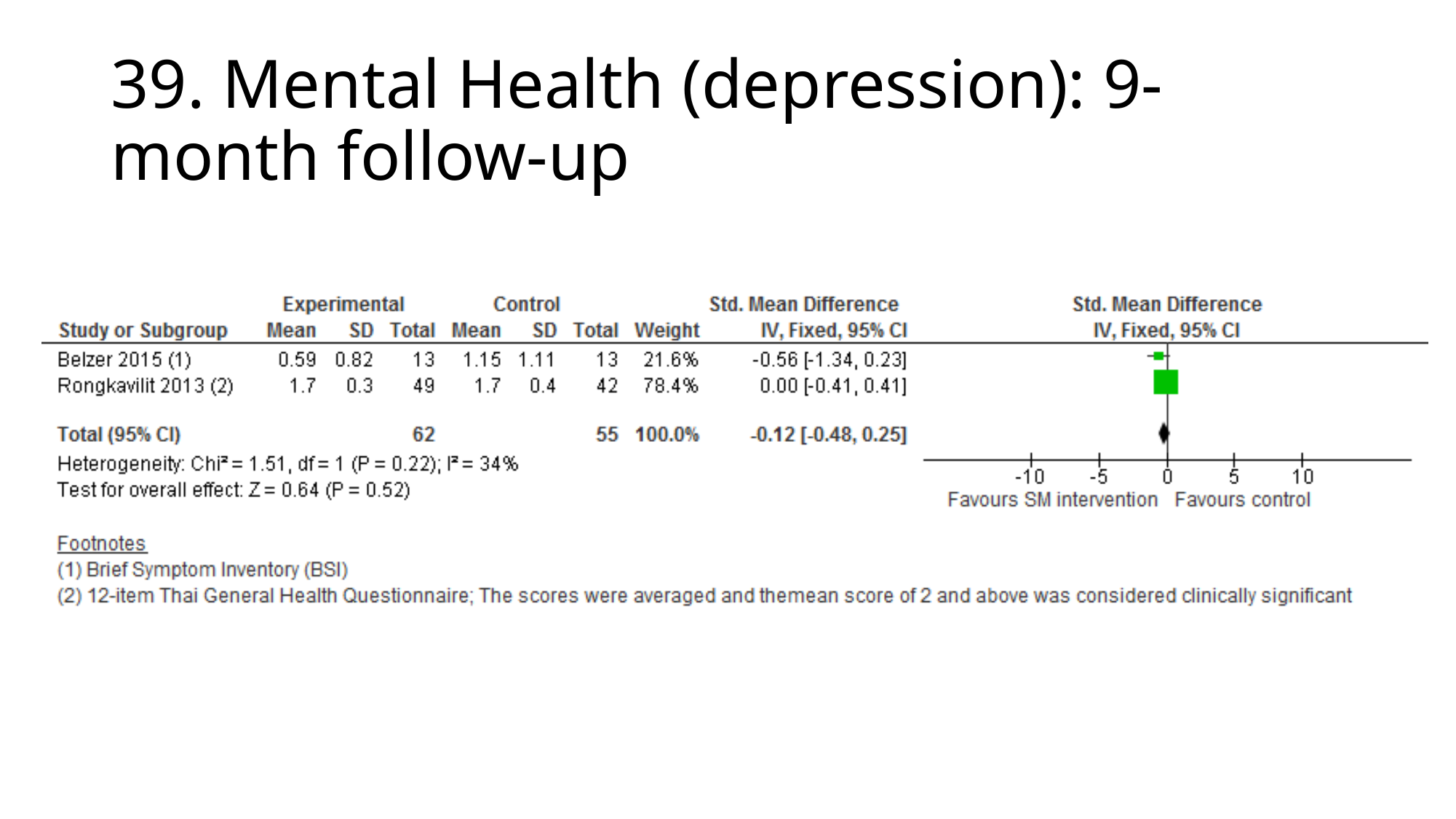

# 39. Mental Health (depression): 9-month follow-up

## Slide 41
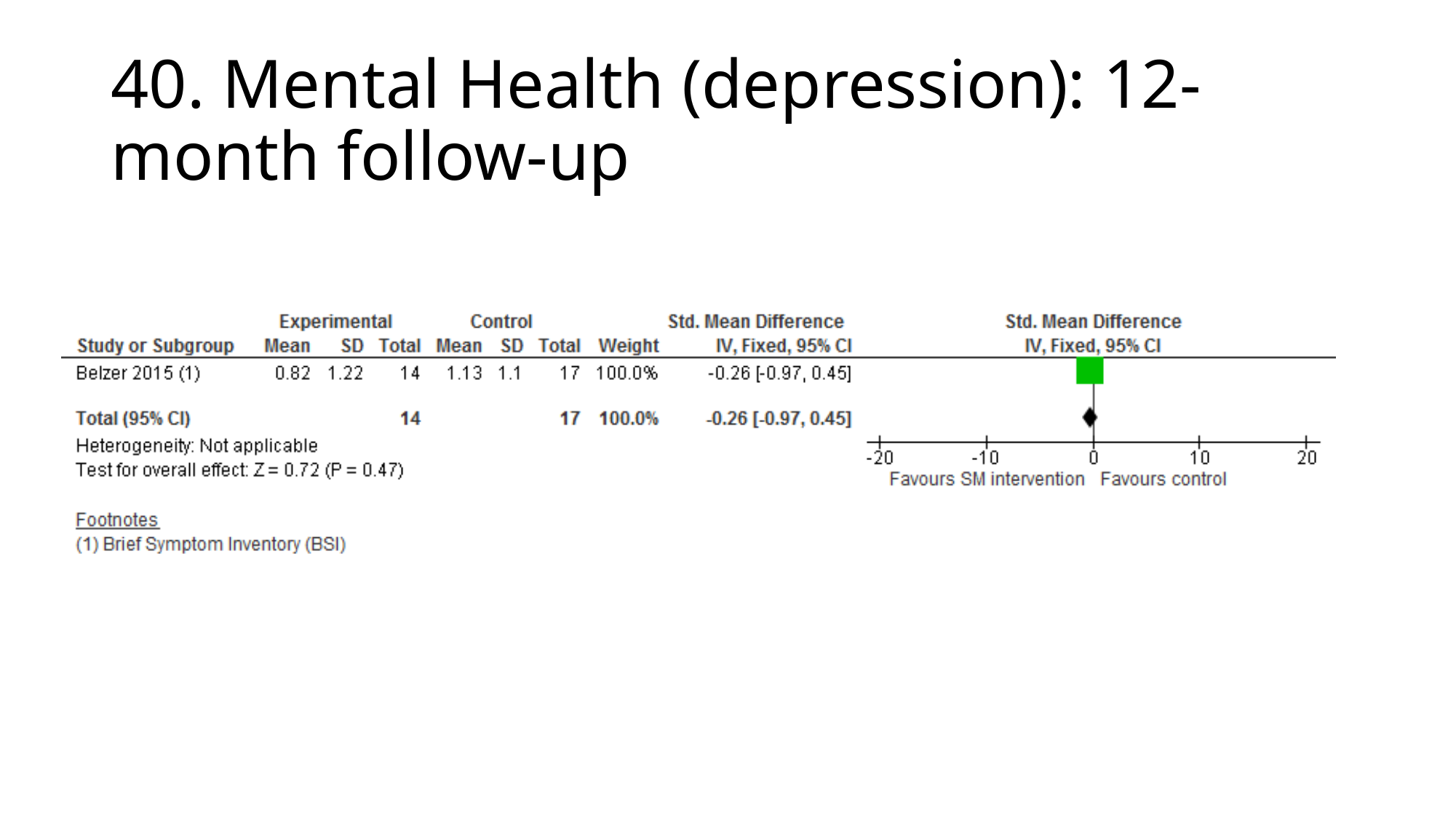

# 40. Mental Health (depression): 12-month follow-up

## Slide 42
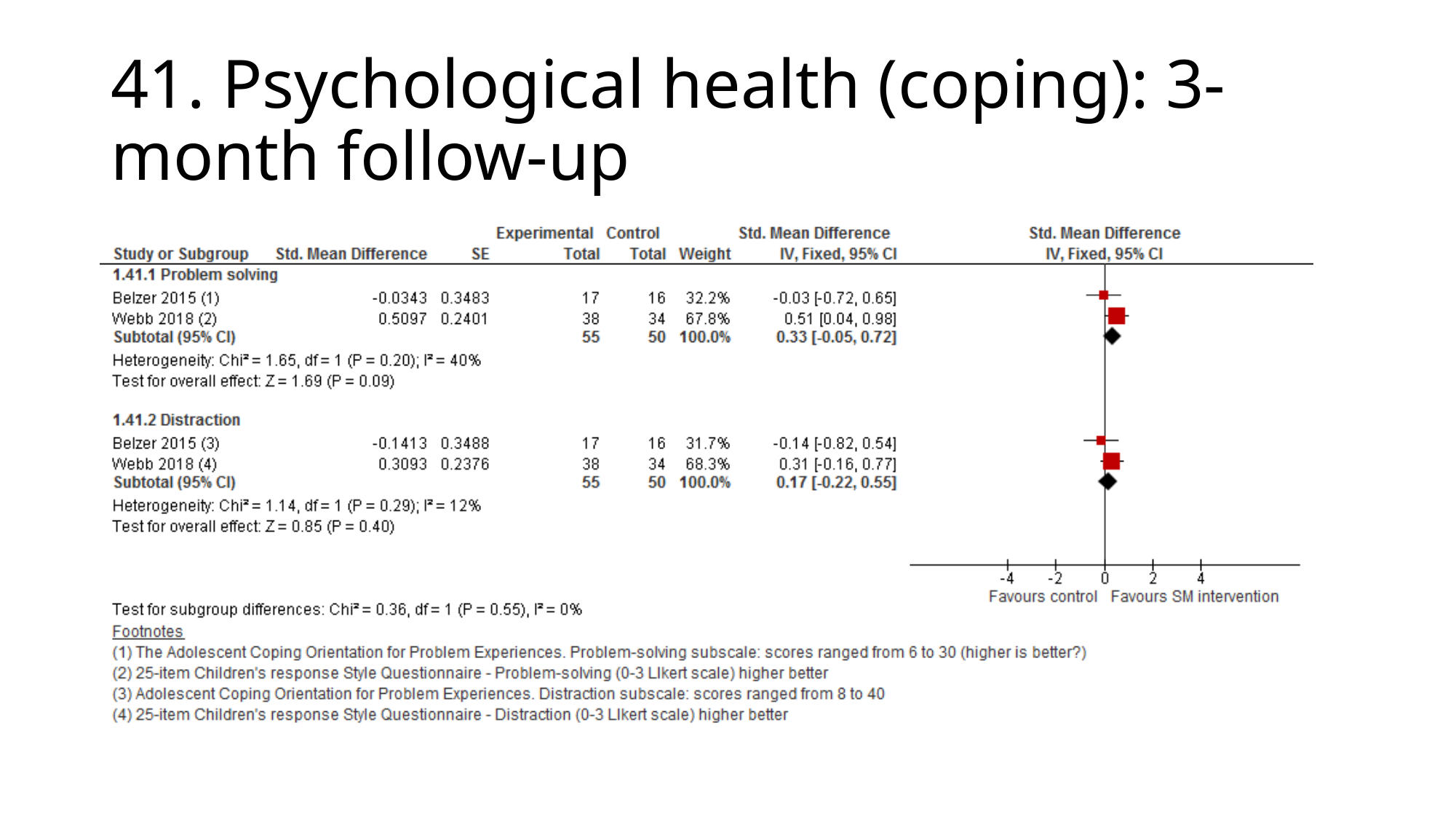

# 41. Psychological health (coping): 3-month follow-up

## Slide 43
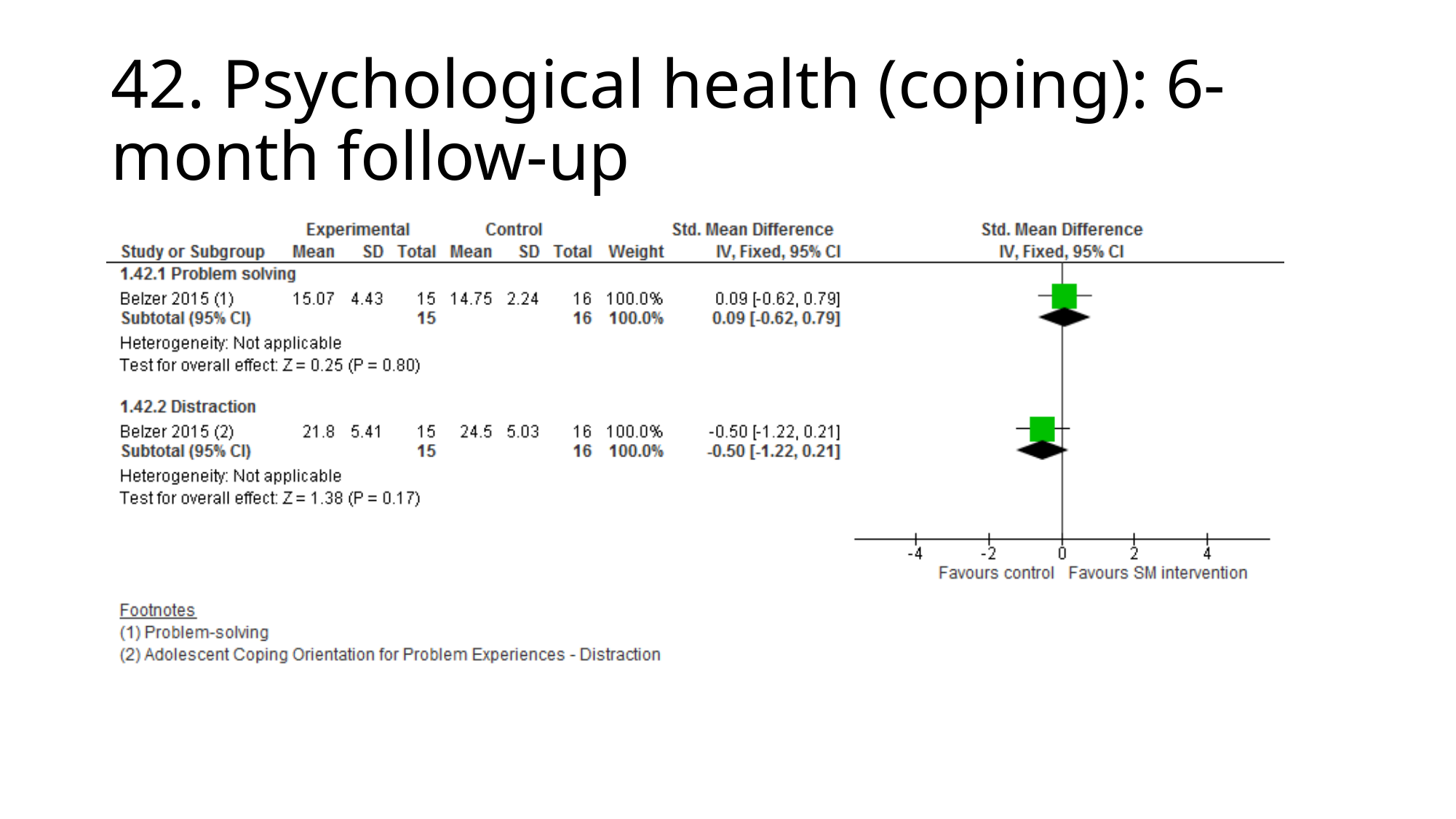

# 42. Psychological health (coping): 6-month follow-up

## Slide 44
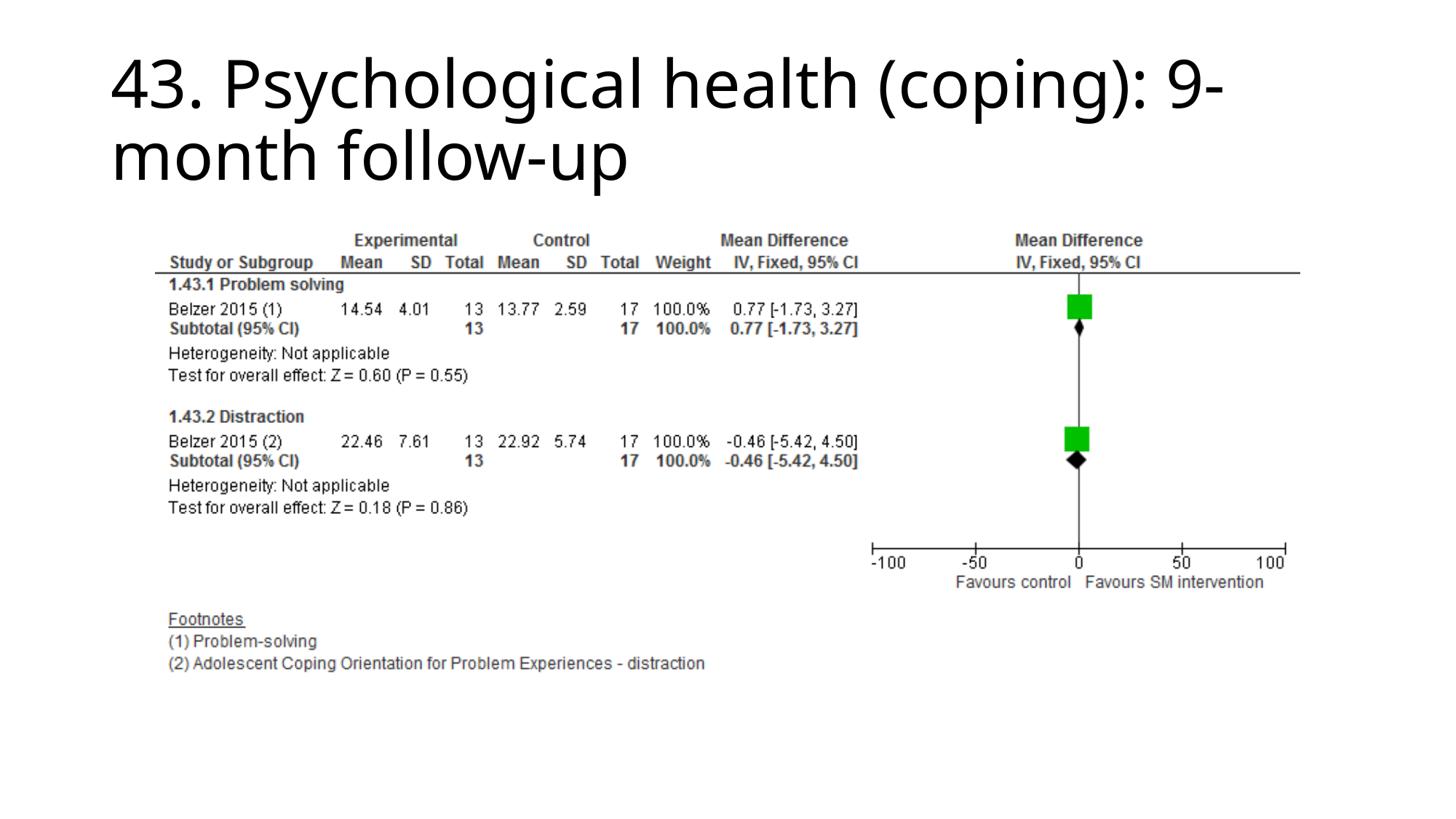

# 43. Psychological health (coping): 9-month follow-up

## Slide 45
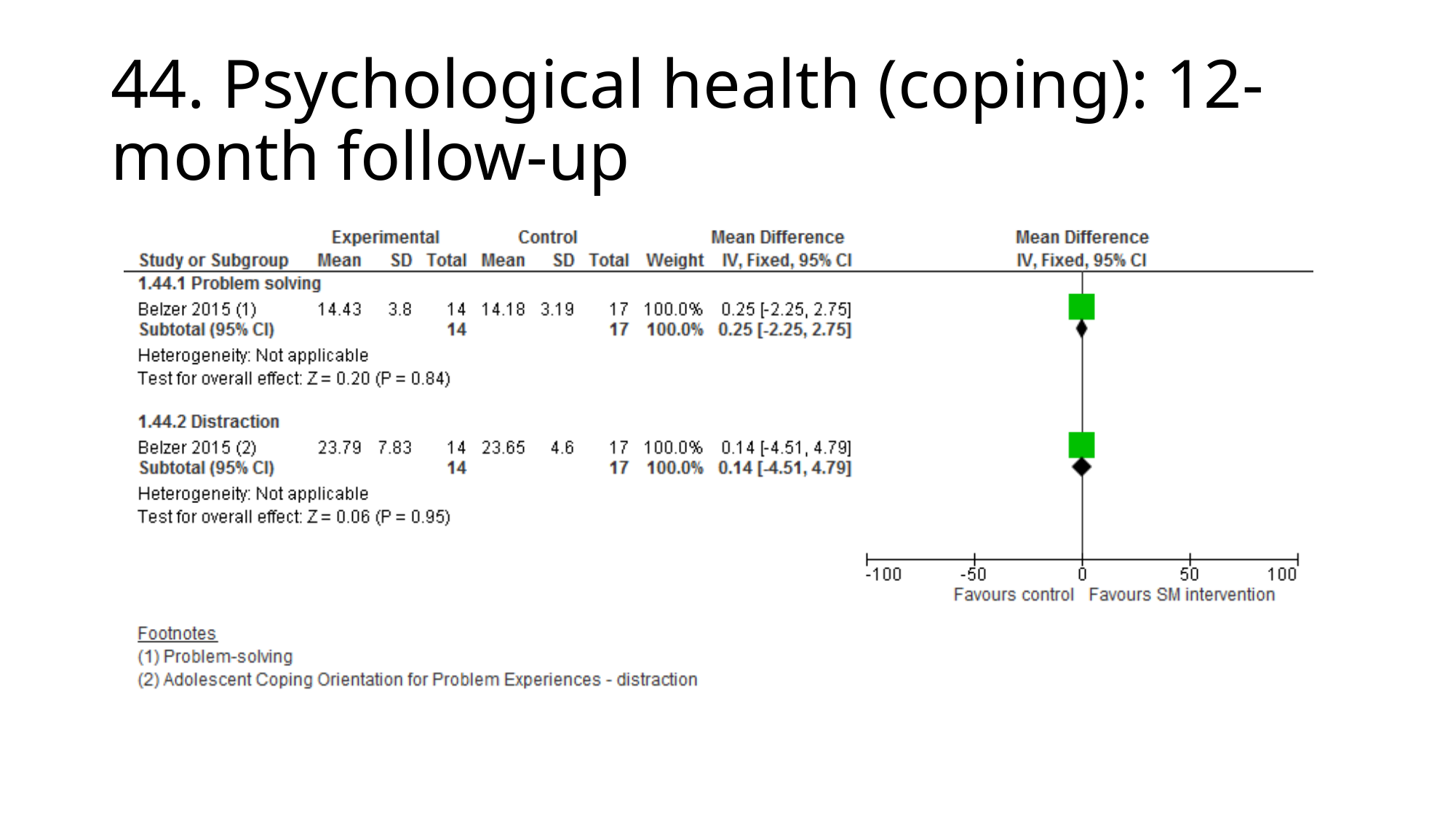

# 44. Psychological health (coping): 12-month follow-up

## Slide 46
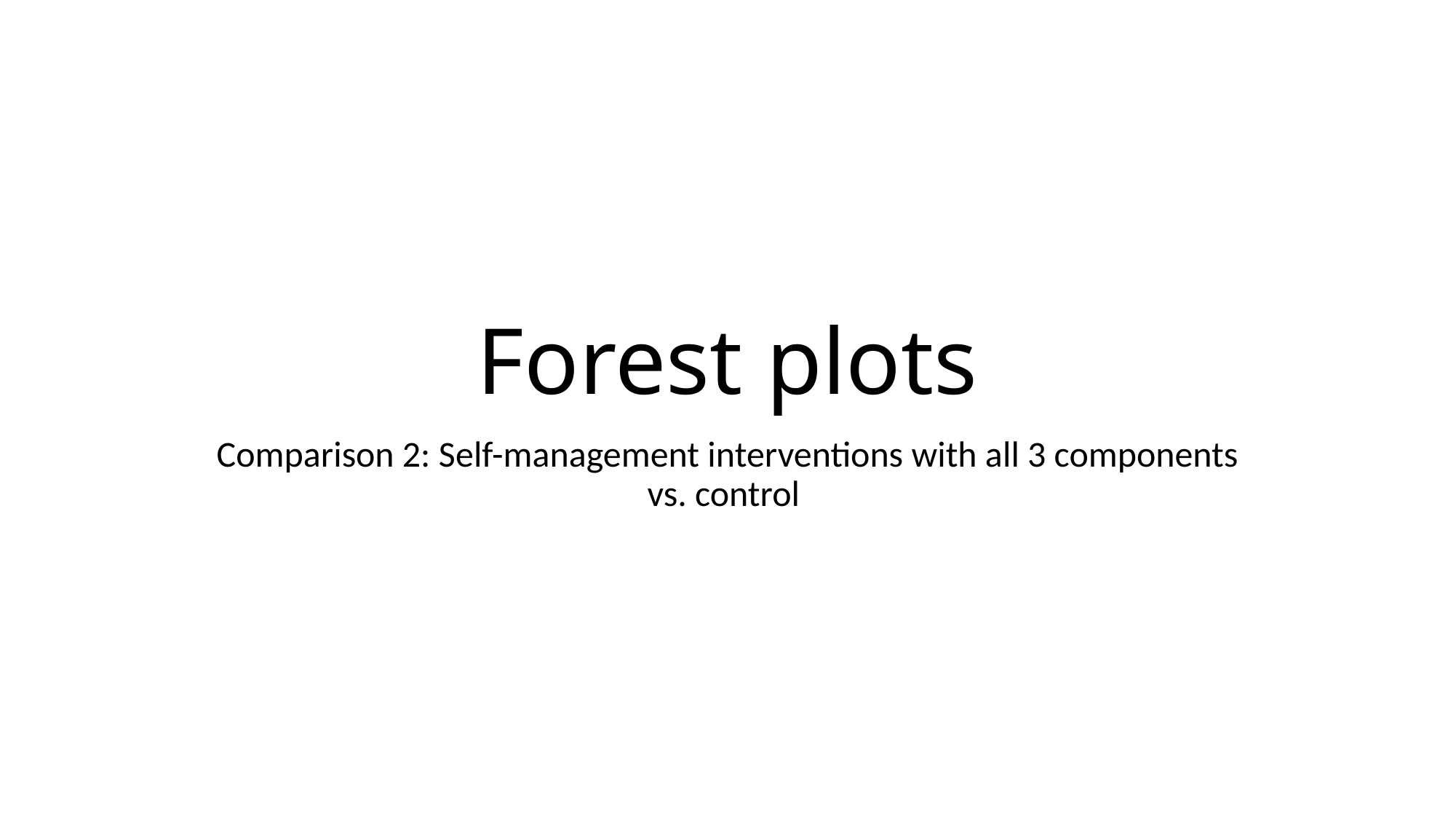

# Forest plots
Comparison 2: Self-management interventions with all 3 components vs. control

## Slide 47
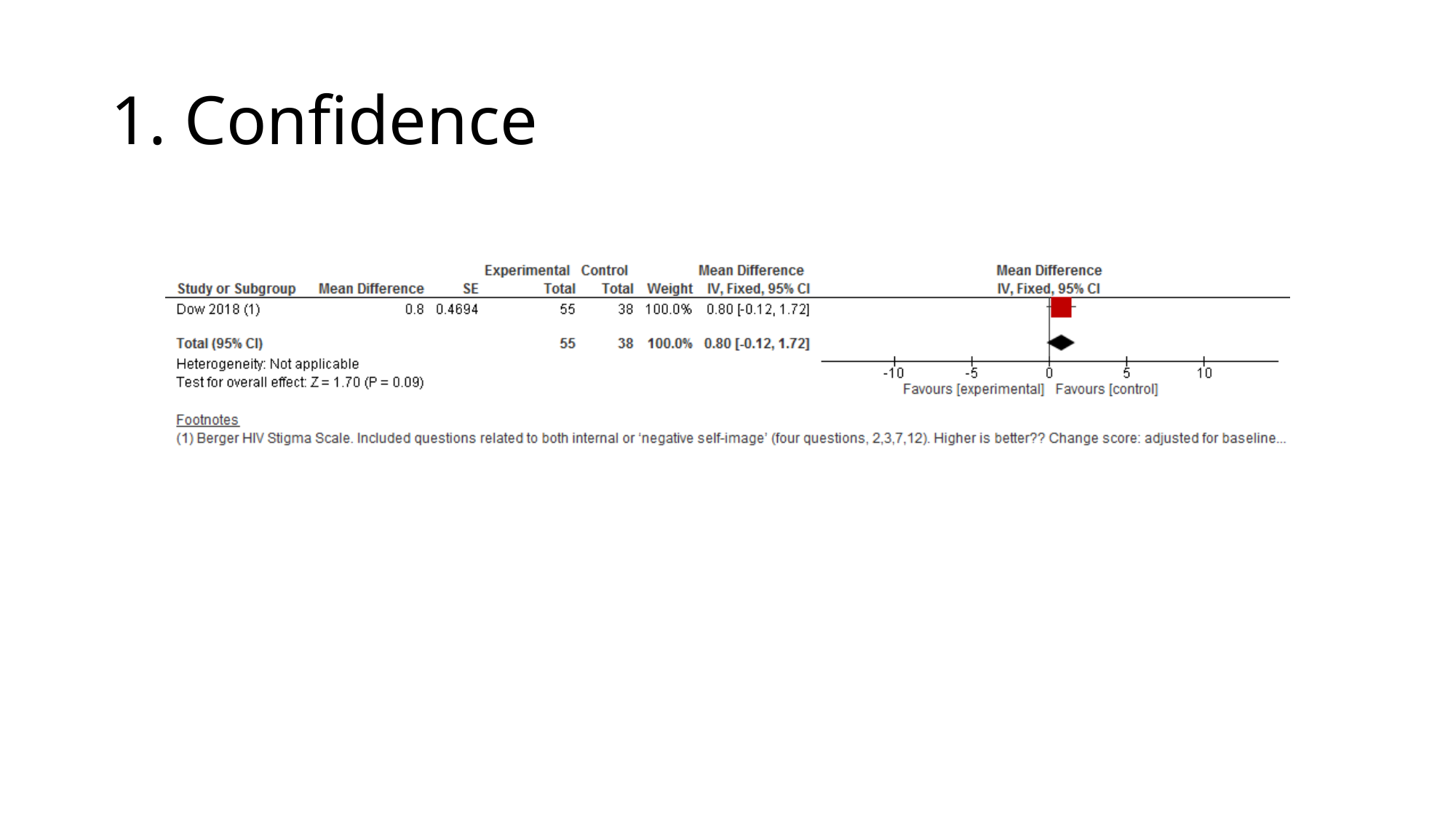

# 1. Confidence

## Slide 48
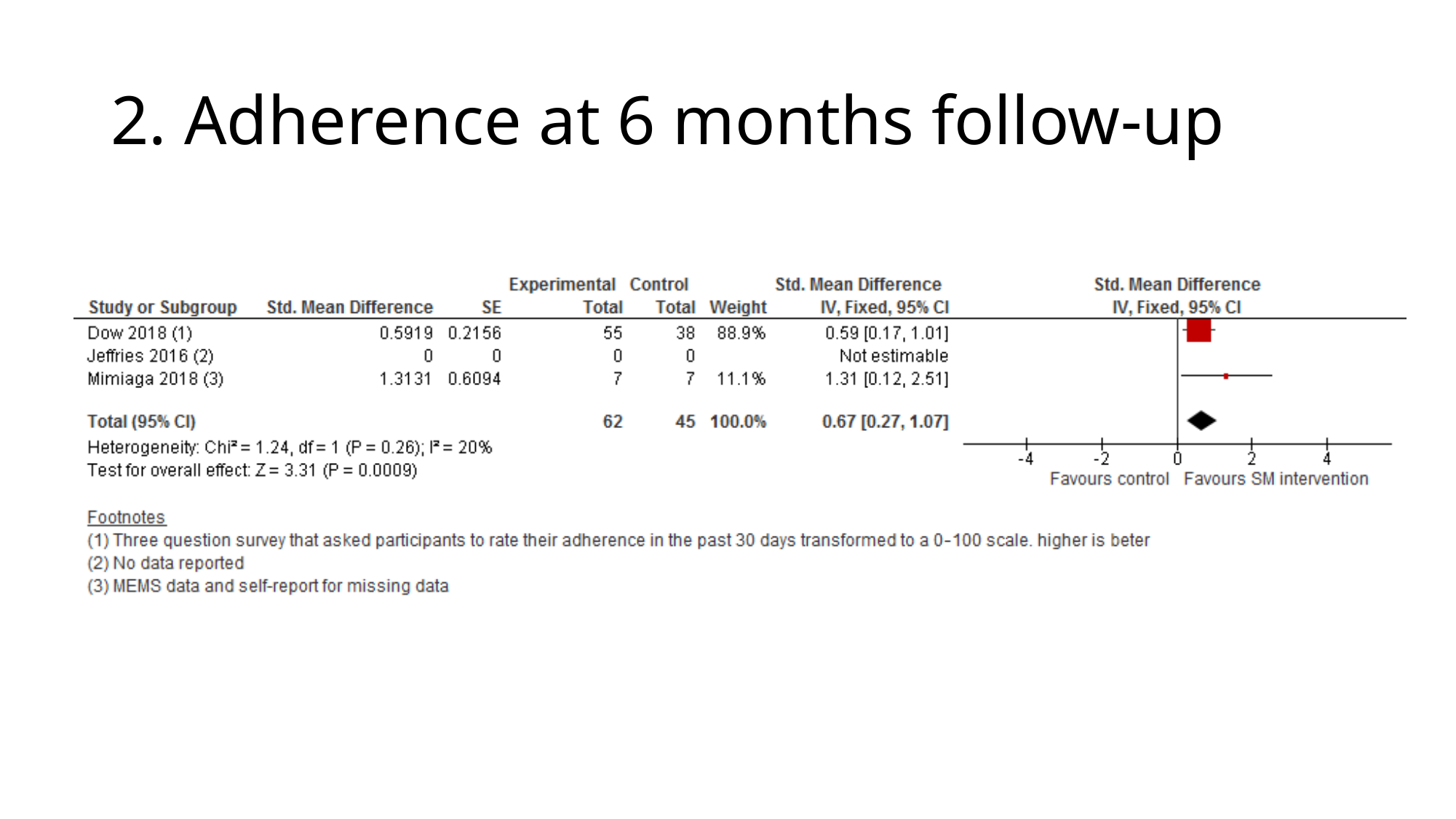

# 2. Adherence at 6 months follow-up

## Slide 49
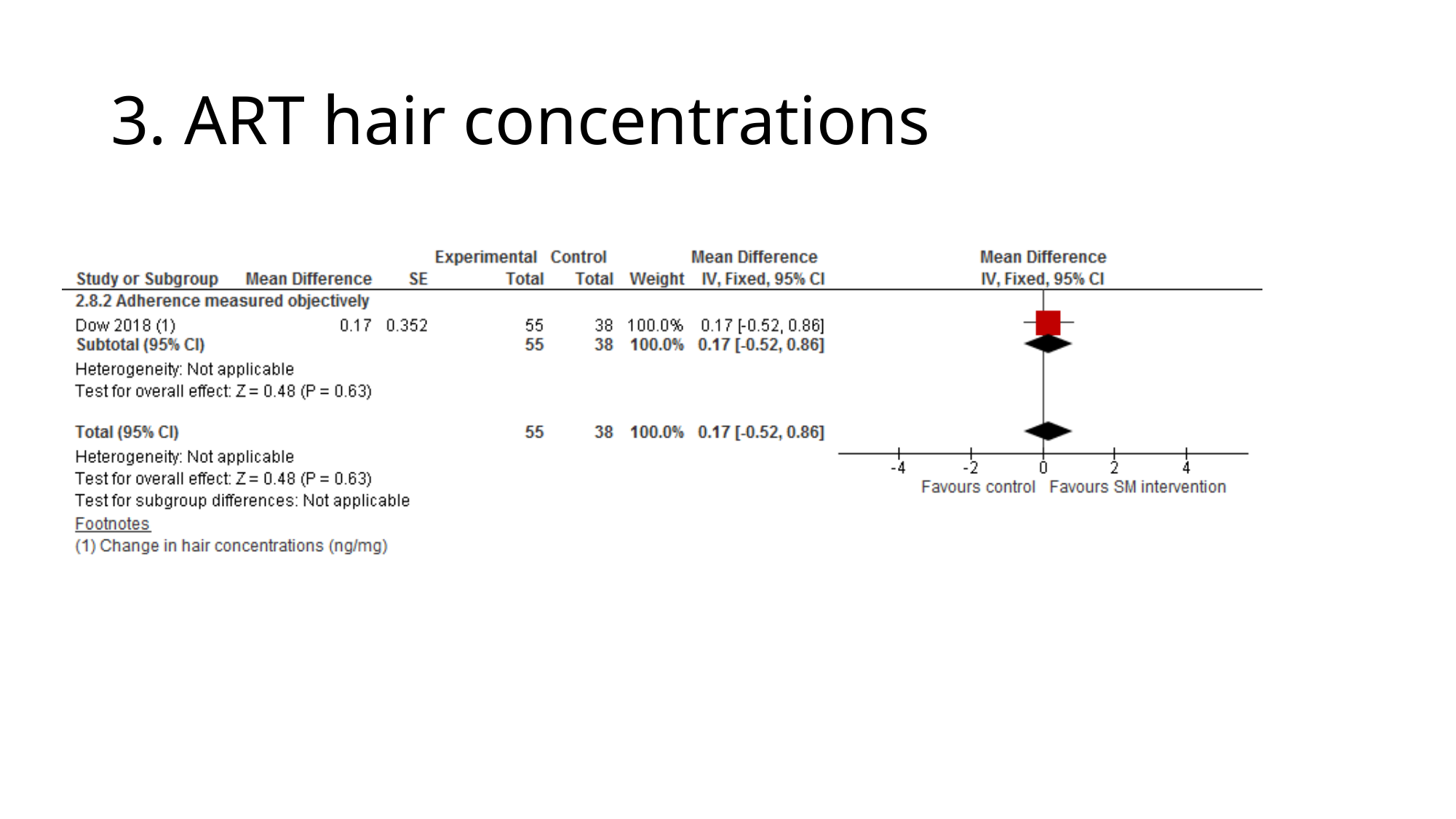

# 3. ART hair concentrations

## Slide 50
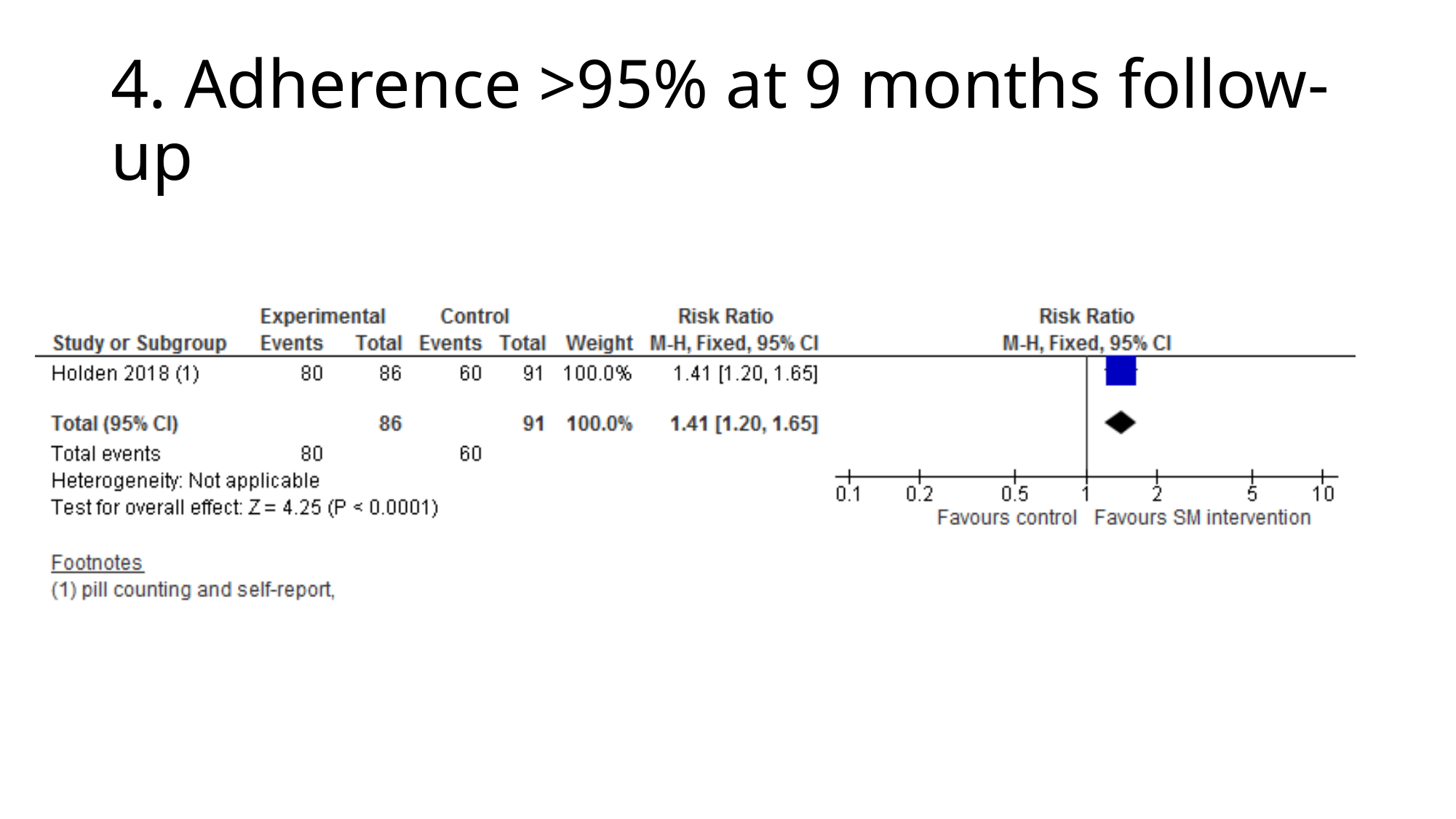

# 4. Adherence >95% at 9 months follow-up

## Slide 51
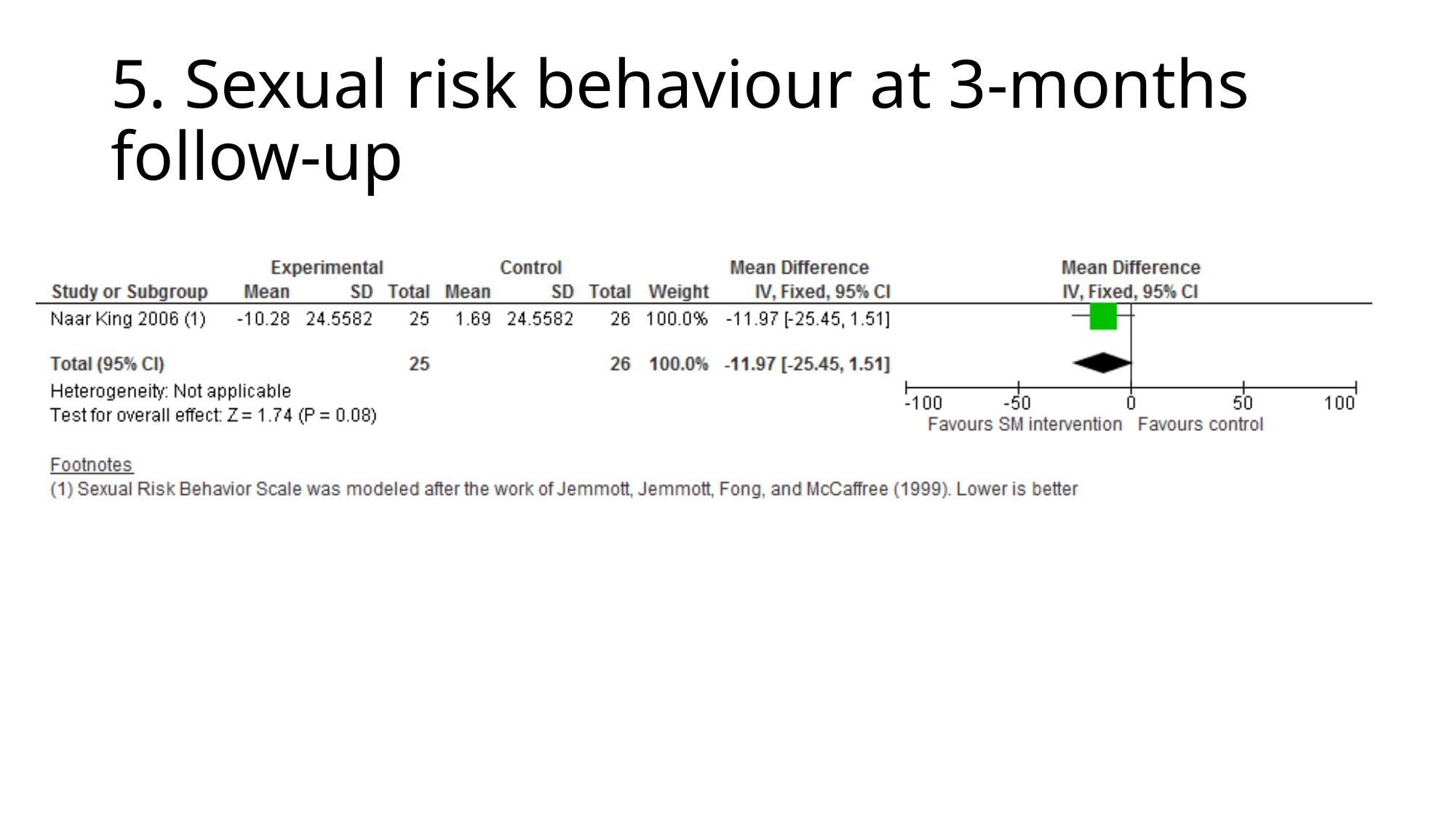

# 5. Sexual risk behaviour at 3-months follow-up

## Slide 52
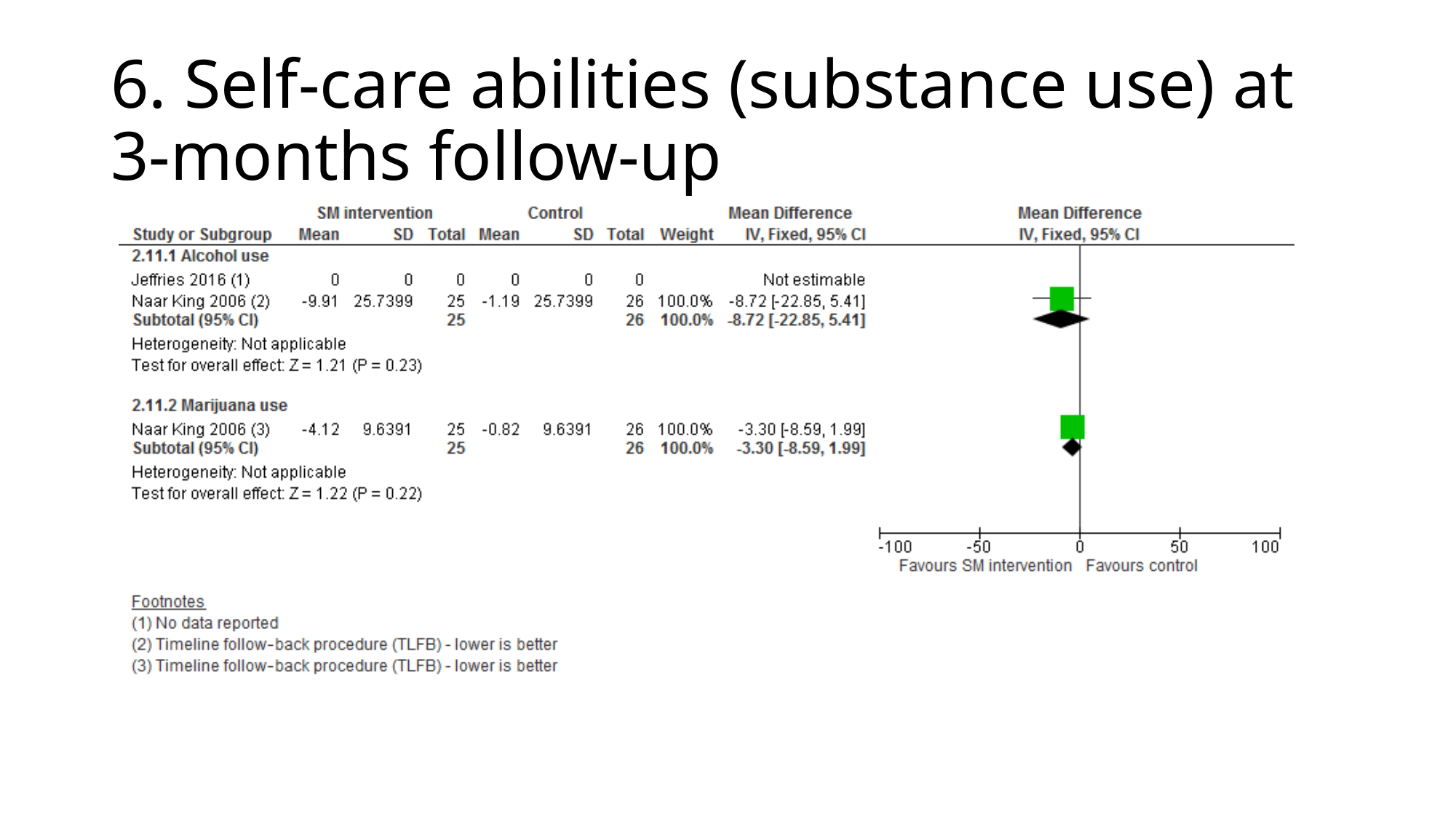

# 6. Self-care abilities (substance use) at 3-months follow-up

## Slide 53
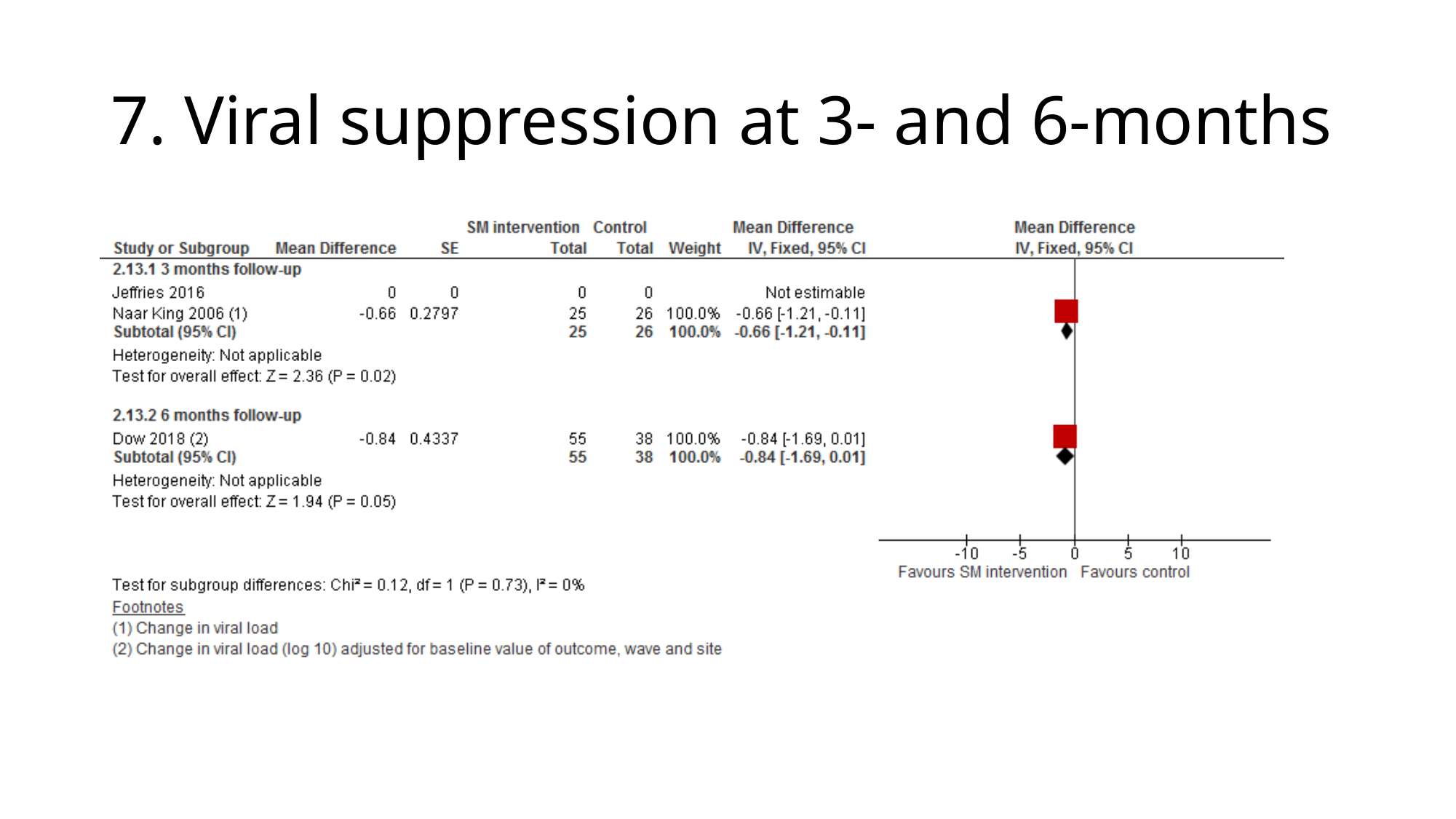

# 7. Viral suppression at 3- and 6-months

## Slide 54
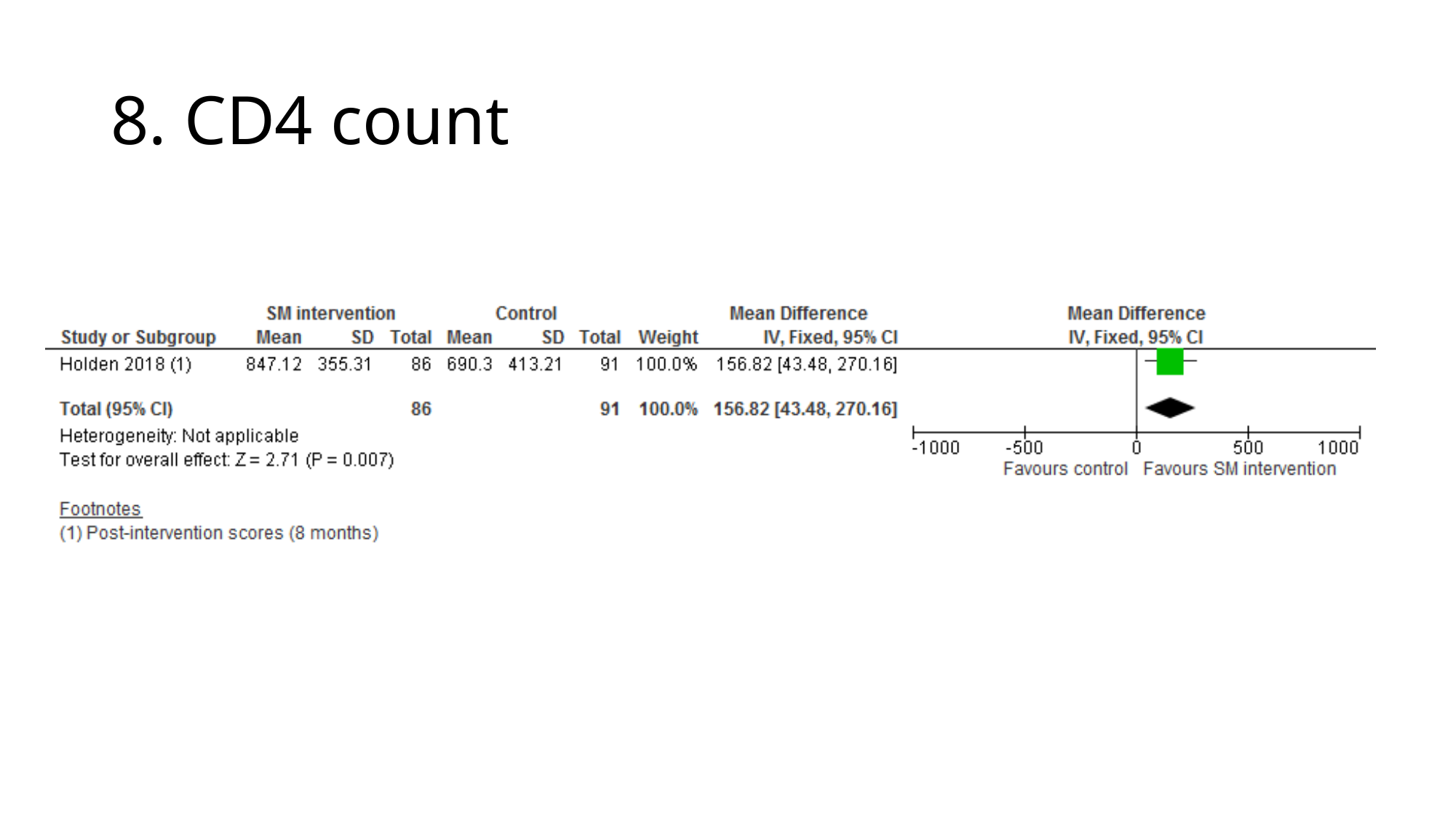

# 8. CD4 count

## Slide 55
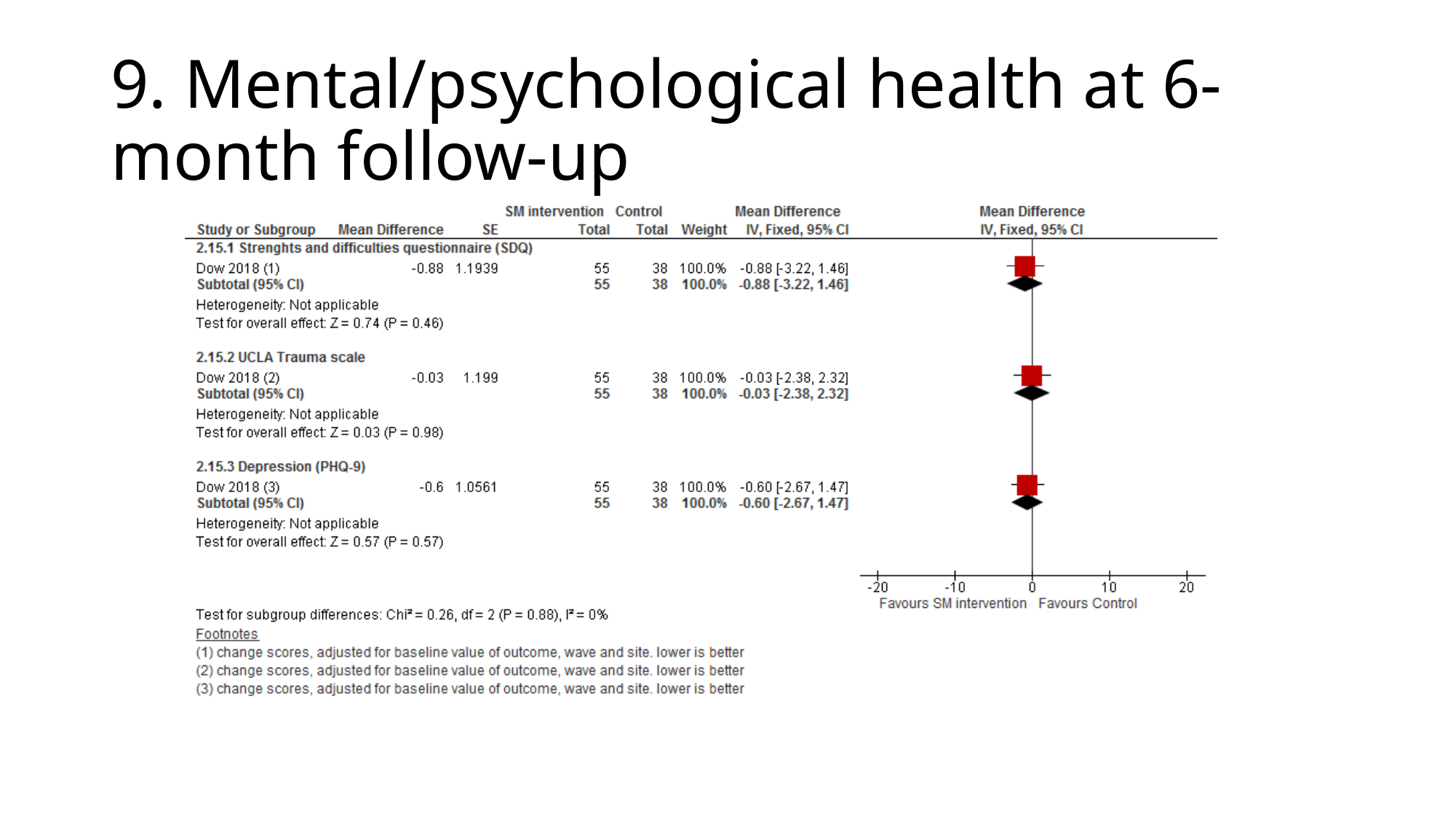

# 9. Mental/psychological health at 6-month follow-up
